# Obstructive Sleep Apnea: Epidemiology, Pathophysiology, Complications, Diagnosis, Management, and Emerging Fibrosis‐Linked Remodeling

**DOI:** 10.1002/mco2.70877

**Published:** 2026-07-30

**Authors:** Nhi Ho Thi Thuy, Thị Hằng Giang Phan, Ali Hussein Eid, Paola Maccioccu, Stefania Camboni, Gavino Casu, Anna Maria Posadino, Roberta Giordo, Gianfranco Pintus

**Affiliations:** ^1^ Department of Biomedical Sciences University of Sassari Sassari Italy; ^2^ Department of Immunology and Pathophysiology University of Medicine and Pharmacy Hue University Hue City Vietnam; ^3^ Department of Basic Medical Sciences College of Medicine QU Health Qatar University Doha Qatar; ^4^ Clinical and Interventional Cardiology Unit Sassari University Hospital Sassari Italy; ^5^ Department of Medicine Surgery and Pharmacy University of Sassari Sassari Italy; ^6^ Department of Human Science For Promotion of Quality of Life University San Raffaele Rome Italy

**Keywords:** fibrosis, hypoxic burden, intermittent hypoxia, obstructive sleep apnea, organ remodeling, personalized management

## Abstract

Obstructive sleep apnea (OSA) is a highly prevalent yet substantially underdiagnosed sleep‐related breathing disorder increasingly recognized as a heterogeneous systemic disease rather than a purely mechanical consequence of recurrent upper‐airway collapse. This review provides an updated and integrated overview of OSA, spanning epidemiology, multifactorial pathophysiology, heterogeneous clinical presentation, major cardiometabolic and end‐organ consequences, diagnostic principles, and evolving personalized management, while also emphasizing the limitations of conventional severity classification, which is predominantly based on the apnea–hypopnea index. We highlight how intermittent hypoxia–reoxygenation, sleep fragmentation, sympathetic activation, intrathoracic pressure stress, endothelial dysfunction, inflammation, oxidative stress, and metabolic dysregulation interact to drive systemic injury, and why physiologically enriched metrics such as hypoxic burden, T90, event duration, sleep‐stage dependency, and endotypic heterogeneity may better capture biological risk than event frequency alone. Within this broader framework, fibrosis‐linked structural remodeling emerges as an important yet under‐supported dimension of OSA pathobiology. Experimental and clinical evidence suggests that OSA may contribute to remodeling of the lungs, liver, kidneys, and heart, although the strength of the evidence and the degree of causal inference vary across organs. By integrating established knowledge with this emerging fibrosis‐oriented perspective, this review defines key mechanistic, diagnostic, and translational priorities for future research and phenotype‐aware clinical stratification.

## Graphical abstract

Obstructive sleep apnea (OSA) is characterized by recurrent upper‐airway collapse, which generates key nocturnal stressors including intermittent hypoxia, sleep fragmentation, intrathoracic pressure stress, and sympathetic activation. These physiological disturbances converge on shared biological mechanisms, including oxidative stress, inflammation, endothelial dysfunction, metabolic dysregulation, and maladaptive repair programs involving TGF‐β and HIF‐1α signaling. Through these interacting pathways, OSA may contribute to tissue injury and structural remodeling across multiple organs, including the lungs, liver, kidneys, and heart. The graphical abstract also highlights that clinically relevant risk extends beyond the apnea–hypopnea index (AHI) alone and may be better captured by multidimensional features such as hypoxic burden, T90, event duration, sleep‐stage dependency, and endotypic heterogeneity. *Abbreviations*: AHI, apnea–hypopnea index; HIF‐1α, hypoxia‐inducible factor‐1 alpha; OSA, obstructive sleep apnea; T90, percentage of sleep time spent with oxygen saturation below 90%; TGF‐β, transforming growth factor beta.

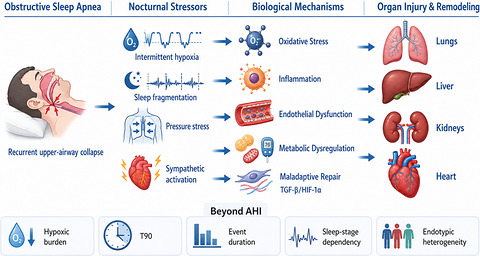

## 1 Introduction

Obstructive sleep apnea (OSA) is among the most prevalent sleep‐related breathing disorders worldwide and remains substantially underdiagnosed despite its large public‐health burden. Recurrent pharyngeal collapse during sleep produces cyclical hypoxemia, reoxygenation injury, sleep fragmentation, and marked intrathoracic pressure swings in a disorder that affects hundreds of millions of adults globally and frequently escapes recognition in routine clinical practice [1, 2, 3, 4].

At the same time, OSA should no longer be considered a biologically uniform exposure [5]. Patients with similar apnea–hypopnea index (AHI) values can carry markedly different physiological loads depending on event duration, desaturation depth, ventilatory deficit, arousal profile, sleep‐stage distribution, and associated autonomic stress [6, 7]. Accordingly, event‐frequency metrics alone do not fully describe disease severity. In contrast, physiologic burden measures, especially hypoxic burden and related composite indices, appear better aligned with downstream cardiovascular risk and broader end‐organ vulnerability [6, 8].

This heterogeneity helps explain why continuous positive airway pressure (CPAP), despite being highly effective in restoring upper‐airway patency and improving symptoms, has not consistently reduced hard cardiovascular outcomes in unselected populations [9]. More recent analyses increasingly suggest that benefit depends on phenotype and physiologic risk enrichment rather than on AHI‐defined severity alone [10, 11], reinforcing the need for mechanism‐informed stratification of OSA patients.

Accordingly, the consequences of OSA extend well beyond disturbed sleep. Intermittent hypoxia (IH)–reoxygenation, arousal‐related sympathetic surges, and large negative intrathoracic pressure oscillations activate oxidative, inflammatory, neurohumoral, metabolic, and endothelial pathways that contribute to cardiovascular, cerebrovascular, and cardiometabolic injury [12, 13, 14].

Within this evolving framework, OSA is increasingly recognized as a heterogeneous systemic disorder whose consequences may extend beyond disturbed sleep and conventional cardiometabolic dysfunction. Recurrent IH and sleep fragmentation activate oxidative stress (OS), immune‐inflammatory pathways, sympathetic signaling, and endothelial dysfunction [15, 16], while also engaging profibrotic molecular programs that can converge on extracellular‐matrix (ECM) remodeling and maladaptive tissue repair [17].

Among these emerging phenotypes, fibrosis‐related structural remodeling deserves particular attention. Experimental and translational studies indicate that OSA‐relevant IH can amplify ROS generation, HIF‐1α signaling, TGF‐β/SMAD activation, inflammasome‐linked inflammation, and stress–response programs that favor fibroblast activation, myofibroblast differentiation, ECM deposition, and progressive tissue stiffening [18, 19, 20, 21].

These mechanisms provide a biologically plausible bridge between OSA and organ‐specific remodeling in the lung, liver, kidney, and heart, and they may also help explain why OSA can worsen the course of established fibrotic disorders such as idiopathic pulmonary fibrosis (IPF) and metabolic dysfunction‐associated steatotic liver disease (MASLD) [21, 22, 23, 24]. However, while these data support the biological plausibility of organ‐specific fibrosis‐linked remodeling, they do not establish that fibrosis is a uniform or general consequence of OSA across organ systems.

The available evidence remains fragmented across organ systems, exposure metrics, and clinical endpoints. It remains unclear which components of nocturnal physiological burden best predict fibrotic signaling, which biomarkers are sufficiently robust for risk stratification, and whether OSA‐directed therapy, particularly CPAP, can modify established remodeling rather than improve respiratory events and symptoms [25, 26, 27].

Accordingly, this review moves from the conventional OSA framework, such as epidemiology, causes, established complications, diagnosis, and management, to an integrated fibrosis‐oriented perspective. After summarizing the contemporary epidemiologic, mechanistic, and phenotype‐aware framework of OSA [5, 11], we examine the major systemic complications and then focus on emerging structural remodeling linked to organ fibrosis, with particular attention to the lungs, liver, kidneys, and heart. This narrative review synthesizes established OSA knowledge and evaluates the emerging hypothesis that OSA‐related physiological burden may amplify organ‐specific fibrotic remodeling, with emphasis on the distinction between mechanistic plausibility, clinical association, and intervention‐level evidence. Literature was identified through PubMed/MEDLINE, Scopus, and Web of Science, with additional manual screening of reference lists and targeted Google Scholar checks where needed. To maintain a contemporary perspective, the review prioritized studies published from 2022 to the time of manuscript preparation, while earlier articles were included selectively when considered seminal or foundational. Search terms combined OSA with epidemiology, pathophysiology, IH, OS, physiological burden metrics, phenotypes/endotypes, diagnosis, management, complications, fibrosis, remodeling, and organ‐specific disease contexts. For established topics, priority was given to recent guidelines, systematic reviews, and meta‐analyses; for emerging fibrosis‐linked remodeling, relevant mechanistic, translational, and organ‐specific clinical studies were also included.

## 2 Epidemiology

OSA epidemiologic estimates depend strongly on three interrelated methodological choices: (i) the diagnostic threshold applied (e.g., AHI ≥5/h, ≥15/h, or ≥30/h); (ii) whether symptoms are required for case definition (OSA syndrome vs. AHI‐defined OSA); and (iii) the hypopnea scoring criteria used (e.g., 3 vs. 4% oxygen desaturation and/or arousal‐based definitions). Within the American Academy of Sleep Medicine (AASM) framework, “recommended” hypopnea scoring relies on ≥3% desaturation and/or arousal, whereas an alternative definition requires ≥4% desaturation—differences that can materially shift AHI distributions and prevalence estimates [28, 29, 30, 31, 32]. In 2023, the AASM released Version 3 of the Scoring Manual, with accredited sleep facilities required to implement the new rules by December 31, 2023, while retaining hypopnea scoring based on a ≥3% desaturation and/or an arousal as the recommended definition and ≥4% desaturation as an optional alternative. These definitional choices have downstream implications for case ascertainment, payer coverage, and comparability across cohorts [33] (AASM, 2023; AASM, 2024).

These definitional effects function as selective filters that preferentially identify or overlook specific OSA phenotypes, rather than serving as simple calibration artifacts. Metrics incorporating event duration, desaturation depth, and cumulative ventilatory or hypoxic stress demonstrate that individuals with identical AHI values may experience markedly different biological exposures and associated risks. Therefore, prevalence estimates based solely on event counts may underestimate clinically significant heterogeneity [6, 7].

Additional variability arises from device and sampling characteristics. Home sleep apnea testing (HSAT) typically cannot score EEG arousals and therefore relies primarily on desaturation‐based criteria, potentially undercounting events that would qualify via arousal on in‐laboratory polysomnography (PSG) [34]. Recent comparisons between PSG‐derived AHI and HSAT‐like respiratory event index (REI) demonstrate systematic REI underestimation, often due to reliance on total recording time and the inability to incorporate arousal‐based hypopneas. This limitation may shift patients into lower severity strata and attenuate prevalence estimates in surveillance systems dominated by home testing [35]. Moreover, night‐to‐night variability can result in meaningful diagnostic misclassification when relying on a single recording; multinight assessments reduce misclassification error and may shift severity categorization [36].

From an epidemiologic perspective, increasing reliance on single‐night HSAT can aggregate two downward biases in the same direction: underdetection of arousal‐related hypopneas and failure to capture night‐to‐night variability. The net effect is a tendency to reclassify intermittently expressed, rapid eye movement (REM)‐predominant, or milder disease into lower‐severity strata when surveillance systems rely heavily on home testing rather than full PSG [35, 37].

Population‐based PSG studies further demonstrate that a substantial proportion of the general population meets objective PSG criteria for sleep‐disordered breathing, including OSA, even in the absence of overt symptoms. Consequently, many affected individuals are not identified in routine clinical practice, leading to systematic underrecognition of the true disease burden [3, 4, 38]. Contemporary evidence highlights a persistent “diagnosed vs. probable” gap. In the French CONSTANCES cohort, the prevalence of treated sleep apnea was markedly lower than the prevalence of individuals at high risk based on validated questionnaires [39]. Administrative health‐record surveillance in England similarly illustrates underestimation: in 2019, diagnosed adult OSA prevalence was 1.40% (≈622,528 individuals), substantially lower than symptom‐based and PSG‐based estimates [40]. In parallel, cross‐national analyses based on self‐reported breathing pauses plus excessive daytime sleepiness (EDS) suggest that “OSA syndrome” may affect approximately 19.5% of working‐age adults in the UK and 22.8% in the USA, with considerable associated productivity losses—underscoring the public‐health relevance of closing the diagnosis and treatment gap [41].

Contemporary PSG cohorts and modeling studies indicate a very large global burden. A landmark literature‐based analysis estimated that among adults aged 30–69 years, approximately 936 million individuals have OSA (AHI ≥5/h) and 425 million have at least moderate OSA (AHI ≥15/h) [1]. Subsequent reviews confirm that prevalence estimates vary substantially by region, diagnostic standards, and ascertainment strategy, and likely remain conservative in settings with limited access to sleep medicine [2, 42]. Country‐specific syntheses highlight marked heterogeneity and large absolute burdens in populous regions. For example, a 2023 systematic review and meta‐analysis estimated that approximately 104 million working‐age adults in India have OSA, including about 47 million with moderate‐to‐severe disease [43]. In China, a multilevel meta‐analysis reported an increase in pooled OSA prevalence from roughly 8% (2000–2005) to approximately 27% (2021–2024) [44]. In the United States, a meta‐analytic estimate suggested that 83.7 million adults (32.4%) had OSA in 2024, based on studies that used AHI4 definitions [45].

Importantly, diagnostic definitions materially influence these estimates. Application of AHI3% criteria (3% desaturation and/or arousal), rather than AHI4%, can substantially increase diagnostic yield, with the largest proportional effects often observed in women, particularly within milder disease categories [33]. Looking forward, modeling studies project further growth in OSA prevalence among US adults aged 30–69 years by 2050, driven primarily by rising obesity prevalence and population aging [46]. Collectively, these data underscore that OSA prevalence is not only high but also methodologically contingent, dynamically evolving, and likely underestimated in routine clinical statistics.

Therefore, the prevalence of OSA should be considered a dynamic measure influenced by the interplay of biological, technological, and policy factors. Greater reliance on restrictive hypopnea definitions, single‐night testing, and claims‐based ascertainment within healthcare systems is likely to increase the discrepancy between diagnosed cases and the actual physiologic burden.

### 2.1 Age

Across population‐based adult cohorts, the epidemiology of OSA supports a broadly age‐associated increase in prevalence and objective severity [47, 48]. However, this relationship should not be interpreted as a simple linear accumulation of respiratory events across the lifespan. Rather, the apparent effect of age is strongly conditioned by methodological architecture, particularly the hypopnea scoring rule adopted, including whether events are scored using arousal criteria and/or 3 versus 4% oxygen desaturation thresholds [47, 49]. Prevalence estimates also vary according to the diagnostic platform used, such as full PSG versus more limited‐channel approaches, and according to the anthropometric and demographic structure of the sampled cohort [49, 50]. Accordingly, comparisons across studies are often not directly interchangeable, because more permissive or contemporary scoring conventions systematically yield higher prevalence estimates [47, 48].

Importantly, aging appears to remodel the clinical phenotype of OSA at least as much as it increases event frequency. Older adults may exhibit higher AHI values while reporting less prominent “classic” EDS [50], suggesting a progressive decoupling between physiological burden and symptom perception [51]. This clinical dissociation is plausibly reinforced by age‐related changes in sleep architecture, multimorbidity, polypharmacy, insomnia symptoms, and overlapping cognitive complaints, all of which can blur syndromic recognition and reduce the sensitivity of symptom‐based screening strategies [51]. Thus, questionnaire‐based triage may underperform precisely in the age strata with the highest objectively measured respiratory event burden [50, 51].

Recent age‐stratified evidence further clarifies that clinically relevant OSA is not confined to older populations. In young adults aged 18–30 years, a recent systematic review estimated an overall prevalence of approximately 16% [52]. However, substantial between‐study heterogeneity indicates that this figure is highly sensitive to AHI thresholds, hypopnea definitions, and recording methodology [52]. By contrast, a meta‐analysis focused on older adults reported a pooled prevalence of approximately 35.9% across 39 studies, including 33,353 participants [53]. However, the very high heterogeneity in that analysis indicates that age‐related increases in OSA burden, although biologically and epidemiologically credible, are still estimated through a methodologically inconsistent evidence base [53]. Age, therefore, should be understood not merely as a driver of higher event counts, but as a modifier of phenotypic expression, symptom salience, and ascertainment probability [50, 51].

In pediatric populations, epidemiologic interpretation requires even greater caution because prevalence estimates are exceptionally sensitive to age window, ascertainment strategy, and diagnostic definition. Landmark population‐based PSG data showed that moderate pediatric sleep‐disordered breathing, such as OSA defined by AHI ≥5 events/h, is relatively uncommon, with a prevalence of approximately 1–2% [54]. In contrast, habitual snoring and milder respiratory disturbance are considerably more frequent [54]. More recent syntheses in preschool‐aged children suggest higher prevalence estimates [55]. However, they also highlight major methodological limitations, including heavy reliance on questionnaire‐based screening, limited use of full PSG confirmation, and, in some studies, older or nonuniform diagnostic thresholds [55]. Thus, apparent temporal increases in pediatric OSA prevalence may partly reflect improved detection intensity and definitional drift rather than a pure increase in biological disease burden [55].

Emerging community‐based data using AHI3% definitions further reinforce the importance of scoring methodology in pediatric prevalence studies [56]. These data suggest that contemporary analytic frameworks can identify substantially higher proportions of previously unrecognized OSA [56], and that disease burden is further enriched in children with overweight or obesity [56]. Taken together, the pediatric literature does not support a single universally applicable prevalence value; rather, it supports a method‐dependent prevalence range whose boundaries are shaped by the scoring rule used, the diagnostic modality applied, and the clinical characteristics of the underlying population [54, 55, 56].

### 2.2 Sex

OSA is consistently more prevalent in men than in women in population‐based cohorts, but this sex difference progressively attenuates with aging and becomes less marked after the menopausal transition [38, 50]. Rather than supporting a static binary model of prevalence, this pattern favors a life‐course framework in which OSA risk in women is progressively unmasked by reproductive aging [57, 58].

A central clinical implication of this sex dimorphism is that OSA in women is often under‐recognized. Compared with men, women more frequently present with less “classic” symptom profiles, in which insomnia, fatigue, poor sleep quality, nonrestorative sleep, mood disturbance, and sleep fragmentation are more prominent than overt EDS or witnessed apneas [59, 60, 61]. This phenotype likely contributes to delayed recognition and diagnosis relative to objective respiratory event [59, 60]. Contemporary analyses further suggest that screening approaches historically shaped around male‐pattern symptom expression may continue to miss clinically relevant disease in women [60, 62].

Beyond symptom presentation, women also appear more likely to experience clinically meaningful disease at lower AHI values [60]. In parallel, REM‐predominant OSA is more frequent in women than in men [63], and women are also more likely to exhibit prolonged inspiratory flow limitation and symptomatically relevant sleep‐disordered breathing despite a lower total AHI [63, 64]. Together, these observations indicate that reliance on global AHI thresholds alone may compress the true clinical burden of OSA in women, particularly when respiratory events cluster during REM sleep or coexist with insomnia/comorbid insomnia and sleep apnea (COMISA) phenotypes [60, 65]. This underdetection may be further amplified when more restrictive hypopnea scoring rules are applied, whereas broader arousal‐inclusive criteria appear to increase diagnostic yield in women [33].

The menopausal transition is increasingly recognized as a biologically important inflection point in female OSA risk [57, 58]. Data from the Wisconsin Sleep Cohort showed that menopausal status is independently associated with increased sleep‐disordered breathing even after adjustment for age and adiposity, with a possible attenuation of risk among women receiving hormone therapy [58]. These findings support the view that sex hormones influence upper‐airway stability and ventilatory control rather than merely tracking chronological age [58, 66]. More recent NHANES‐based analyses further suggest that visceral adiposity may partially mediate the postmenopausal increase in OSA symptom burden independently of body mass index (BMI) [57]. Although earlier observational work suggested that hormone therapy might attenuate sleep‐disordered breathing [67], a subsequent reappraisal indicated that this association may be explained, at least in part, by healthy‐user bias rather than by unequivocal causal protection [68].

Pregnancy represents another sex‐specific physiological condition in which OSA may newly emerge or worsen across gestation [69, 70]. In a prospective cohort with polysomnographic assessment in the first and third trimesters, OSA prevalence increased from 10.5% early in pregnancy to 26.7% in late gestation [70]. Similarly, in a high‐risk cohort, incident sleep‐disordered breathing developed in approximately 20% of women over the course of pregnancy [69]. Prospective data further support an independent association between sleep‐disordered breathing and hypertensive disorders of pregnancy, preeclampsia, and gestational diabetes [69]. At the same time, this literature should be interpreted cautiously, as recent reviews emphasize substantial heterogeneity in study design, cohort enrichment, and diagnostic methodology across pregnancy‐related OSA studies [71]. Large longitudinal PSG‐linked datasets spanning pregnancy and the postpartum period likewise indicate that sleep disorders, including sleep‐disordered breathing, are common and dynamically distributed across the perinatal timeline rather than temporally fixed [71, 72].

### 2.3 BMI and Obesity

Excess adiposity is a major modifiable epidemiologic risk factor for adult OSA. However, BMI should be interpreted as a population‐level risk‐enrichment marker rather than as a disease‐defining biological variable. Community‐based data show that BMI and central adiposity measures are positively associated with OSA burden, and confirm that clinically relevant OSA is not confined to people with obesity [50, 73]. More recent pooled evidence has sharpened this concept by showing that a substantial proportion of adults with OSA fall outside the conventional obesity category, indicating that BMI captures only one dimension of susceptibility [73].

Mechanistically, BMI is best viewed as a coarse surrogate for the trait that matters more directly during sleep: upper‐airway collapsibility. That phenotype is commonly indexed by pharyngeal critical closing pressure (Pcrit), and a recent systematic review/meta‐analysis identified hyoid position, tongue volume, pharyngeal length, and waist circumference among the anatomical variables most strongly associated with collapsibility [74]. This aspect supports the view that airway mechanics are shaped more directly by regional structure than by body mass alone.

Magnetic resonance imaging (MRI)‐based anatomical studies provide the substrate for this relationship. Volumetric imaging demonstrated that patients with OSA exhibit enlarged lateral pharyngeal walls, increased tongue volume, and greater total peripharyngeal soft tissue [75]. Later work refined this phenotype by showing increased fat deposition within the tongue, particularly at the tongue base, in obese patients with OSA compared with obese controls without OSA [76]. These findings indicate that obesity promotes OSA not simply through higher total body weight, but through selective soft‐tissue loading of the collapsible pharyngeal segment.

Regional fat distribution is therefore more informative than BMI alone. Cervical, submental, and visceral adiposity promote OSA through both local tissue loading around the airway and remote effects on respiratory mechanics. Excess central adiposity lowers functional residual capacity and end‐expiratory lung volume, thereby reducing caudal tracheal traction and pharyngeal wall tension [77, 78]. As a result, the upper airway becomes more collapsible under the negative inspiratory pressures generated during sleep.

The obesity–OSA relationship is not purely anatomical. Contemporary syntheses indicate that reduced lung volume can interact with ventilatory‐control instability and impaired neuromuscular compensation [78]. In parallel, landmark experimental work showed that leptin deficiency impairs active upper‐airway neuromuscular responses and that leptin replacement can improve compensatory airway function without materially changing passive mechanical loads [79]. Thus, obesity may destabilize the airway both by increasing passive collapsibility and by weakening the active compensatory responses needed to maintain patency during sleep.

Longitudinal data strongly support a dose–response relationship between body weight change and OSA severity. In the Wisconsin Sleep Cohort, a 10% weight gain predicted an approximately 32% increase in AHI, whereas a 10% weight loss predicted an approximately 26% reduction in AHI [80]. The same study also showed that a 10% increase in weight was associated with a substantially greater risk of developing moderate‐to‐severe sleep‐disordered breathing [80]. These findings remain among the clearest demonstrations that adiposity dynamically modulates OSA severity over time.

Interventional evidence reaches a similar conclusion while also exposing the limits of a BMI‐centric model. In the 10‐year Sleep AHEAD follow‐up, OSA remission was more frequent after intensive lifestyle intervention than after diabetes support and education [81]. However, complete remission remained far from universal [81], implying that excess adiposity often amplifies pre‐existing airway vulnerability rather than fully explaining disease expression.

Imaging‐based interventional studies further show that weight loss remodels the airway itself. Reduction in body weight is associated with decreases in tongue fat and upper‐airway soft tissue volume, along with enlargement of the velopharyngeal airway [82, 83]. Mediation analyses suggest that a reduction in tongue‐fat volume is an important intermediary linking weight loss to AHI improvement [83]. Biologically, this means that weight loss matters in OSA not merely because the patient becomes lighter, but because specific airway structures change in size and composition.

Recent Phase 3 evidence extends this causal framework beyond lifestyle intervention. In adults with obesity and moderate‐to‐severe OSA, tirzepatide reduced AHI, body weight, hypoxic burden, high‐sensitivity C‐reactive protein (CRP), and systolic blood pressure (BP) [84]. These findings reinforce the clinical importance of weight‐targeted therapy in obesity‐associated OSA, while also underscoring that such benefit will vary with the degree to which a given patient's disease is adiposity driven.

Conversely, nonobese OSA should not be dismissed as epidemiologic residue. Trait‐based models indicate that once airway anatomy is only moderately vulnerable rather than frankly normal or severely compromised, nonanatomical traits such as low arousal threshold, impaired upper‐airway muscle responsiveness, and high loop gain can become decisive determinants of disease expression [85]. Recent commentary on OSA in people without obesity similarly emphasizes that relatively mild anatomical compromise may interact with low arousal threshold and other nonanatomical endotypes, helping explain why BMI‐only screening frameworks miss a substantial disease burden [73, 86].

Taken together, these findings indicate that obesity is a dominant but nonexclusive amplifier of OSA pathogenesis. BMI remains clinically useful, but the biologically operative links run through regional fat distribution, upper‐airway soft‐tissue remodeling, lung‐volume dependence, and interaction with nonanatomical endotypes [73, 78]. Framed this way, the obesity–OSA relationship becomes mechanistically more precise and better able to explain both why obesity is such a strong risk factor and why OSA may persist after weight loss or arise in individuals without obesity.

### 2.4 Race/Ethnicity, Social Determinants, and Measurement Bias

Racial/ethnic and socioeconomic disparities shape OSA epidemiology at multiple levels, including the distribution of risk factors, access to diagnostic testing, and treatment uptake. Contemporary evidence suggests that historically marginalized groups may carry a greater disease burden and present later in the clinical course, while also facing structural barriers to diagnosis and long‐term adherence to therapy [87, 88]. Importantly, these inequities should not be interpreted as simple reflections of ancestry. More recent conceptual frameworks place upstream structural determinants, including housing conditions, shift work, environmental stressors, neighborhood disadvantage, language barriers, insurance design, and culturally mismatched screening pathways, between biologic susceptibility and observed diagnosis rates, indicating that administrative “prevalence” may partly reflect the reach of the health system as much as the true frequency of disease [23, 89]. Policy structures may further widen these gaps: an American Thoracic Society policy statement argued that rigid positive airway pressure (PAP) adherence thresholds linked to insurance coverage may disproportionately penalize patients facing social and structural barriers, and therefore advocated for more patient‐centered metrics and policy reform [90].

Disparities are also evident after diagnosis. In a national EHR‐linked cohort from the NIH *All of Us* Research Program, including 8518 adults with diagnosed OSA, Hispanic adults had lower adjusted odds of receiving CPAP therapy than non‐Hispanic White adults, even after accounting for socioeconomic factors and healthcare utilization [91]. These findings indicate that inequity in OSA extends beyond disease recognition to include access to treatment and sustained therapeutic implementation.

Measurement choice can also influence how disparities appear. Objective at‐home testing in diverse cohorts suggests that race/ethnicity‐ and sex‐specific differences may become more visible when REM‐specific indices are examined rather than global respiratory event indices. In a cohort of older adults studied with an United States Food and Drug Administration (US FDA)‐cleared peripheral arterial tonometry (PAT)‐based home sleep test, Black participants, particularly Black women, showed higher REM‐stage REI, whereas non‐REM (NREM) indices were similar across groups [92]. This observation is methodologically important because averaging respiratory burden across all sleep stages may dilute signals of disparity concentrated in REM sleep [92]. Stage‐specific metrics may therefore be necessary to uncover clinically relevant inequities that remain obscured when only overall REI3% or similar global thresholds are used [92].

Potential bias may also result from the choice of diagnostic modality. Observed prevalence patterns can vary depending on whether OSA is assessed using full PSG, respiratory polygraphy, or HSAT, as well as the algorithms implemented in specific devices. In this regard, recent guidance documents recommend that the selection of test modality should correspond to the clinical question, and that HSAT‐derived metrics must be interpreted within their physiological and technical constraints [93, 94]. In comparative epidemiology, this means that part of the observed between‐group variation may reflect measurement architecture rather than purely biological disease differences.

Lifestyle exposures add a further layer of complexity. Alcohol can increase upper‐airway collapsibility and has been associated with greater OSA risk in several cohorts, with some studies suggesting sex‐specific patterns [50]. In nationally representative Korean data including approximately 11,859 individuals, alcohol use disorder was associated with higher odds of OSA risk, with dose–response relationships observed for both drinking frequency and binge drinking [95]. A recent meta‐analysis across substance exposures likewise found higher AHI among alcohol users. In contrast, tobacco‐related differences were smaller and less consistently significant, highlighting the importance of careful confounding control and accurate exposure quantification [96]. Smoking remains biologically plausible as a contributor through upper‐airway inflammation, but the magnitude of association varies and is often confounded by adiposity and coexposures [50]. In NHANES‐linked analyses using the STOP‐Bang risk index, current and former smokers had higher OSA risk, particularly men, and longer smoking cessation appeared to attenuate this risk [97].

OSA is also highly prevalent in chronic disease populations, particularly in cardiometabolic disease and chronic kidney disease (CKD). A recent meta‐analysis reported substantial penetrance of sleep apnea, including OSA, across CKD stages, and in end‐stage kidney disease [98]. At the same time, clinical studies suggest that OSA risk increases with advancing CKD severity [99]. A 2024 systematic review and meta‐analysis of 47 studies, including approximately 223,967 participants, estimated a global OSA prevalence of 39% in CKD populations and identified an association between OSA and increased all‐cause mortality risk [100]. These data support routine evaluation for sleep apnea within nephrology care pathways [100]. Mechanistically, the association is likely bidirectional: fluid retention, uremia‐related neuromuscular dysfunction, and altered chemoreflex control may increase upper‐airway instability, whereas recurrent nocturnal hypoxemia and sympathetic activation may further worsen cardiorenal risk [100]. Thus, in CKD, OSA may be more than a clustered comorbidity; it may act as a clinically relevant amplifier of progression and mortality.

A similarly strong epidemiologic signal is emerging in fibrotic lung disease, especially IPF. Early PSG‐based studies already reported high OSA prevalence in IPF, even in patients without marked daytime sleepiness [101], and later observational work suggested associations between OSA and poorer outcomes [102]. A systematic review and meta‐analysis concluded that OSA is prevalent in IPF [103], while, among patients with non‐IPF fibrotic interstitial lung disease (ILD), restrictive physiological features, such as reduced total lung capacity (TLC), have been identified as predictors of OSA risk [104]. A more recent PRISMA‐guided meta‐analysis of IPF cohorts, including seven studies with a pooled sample of 411 patients, reported an OSA prevalence of approximately 70%, albeit with substantial heterogeneity [22]. These findings strengthen the rationale for systematic screening in fibrotic ILD populations [22]. Pathophysiologically, reduced lung volumes may diminish caudal traction on the pharynx and increase upper‐airway collapsibility, while baseline parenchymal gas‐exchange impairment amplifies the oximetric impact of each obstructive event [22, 104]. As a result, nocturnal desaturation metrics in IPF/ILD may reflect both airway obstruction and fibrotic lung physiology, complicating direct comparisons with community OSA cohorts [22, 104].

OSA is increasingly recognized as a contributor to the pathways leading to hepatic steatosis and fibrosis. Systematic reviews demonstrate associations between OSA and nonalcoholic fatty liver disease (NAFLD), nonalcoholic steatohepatitis (NASH), and liver fibrosis, although many datasets are substantially confounded by or mediated through adiposity [23, 105, 106]. Following the adoption of updated nomenclature, these associations are now increasingly examined within the framework of MASLD. The 2024 EASL–EASD–EASO clinical practice guideline identifies OSA as a relevant comorbidity in MASLD care pathways and emphasizes the importance of integrated cardiometabolic risk management [107]. However, evidence supporting the impact of OSA treatment on hepatic outcomes remains limited. A 2025 systematic review concluded that CPAP therapy may modestly improve liver enzymes and selected metabolic parameters. Nevertheless, evidence for significant improvement in steatosis or fibrosis remains heterogeneous and of low certainty [26]. The epidemiologic association is therefore mechanistically plausible but remains causally difficult to disentangle, as visceral adiposity and insulin resistance serve both as shared confounders and intermediary pathways [23, 26].

Beyond lifestyle and comorbid disease, OSA susceptibility is also shaped by craniofacial anatomy, family history, genetic risk, environmental exposures, and broader social determinants of health. Racial/ethnic differences are visible not only in prevalence, but also in presentation, diagnosis, and treatment. For example, Asian craniofacial risk profiles may predispose to OSA at lower BMI, whereas in other populations, a greater contribution may come from obesity burden, airway anatomy, healthcare access, and delayed recognition [108]. Interethnic cephalometric comparisons support this phenotype‐specific interpretation: in Chinese versus Caucasian patients matched for BMI or AHI severity, craniofacial bony restriction was more prominent among Chinese participants, whereas Caucasian participants showed a greater obesity burden at similar OSA severity [109]. Large‐scale genomic studies also support a heritable component to sleep apnea risk and point to potential gene–environment interactions that may vary across populations [110]. These observations argue against treating race/ethnicity as a single biological category. In some groups, craniofacial restriction may shift disease expression toward lower BMI thresholds, whereas in others, obesity, social deprivation, under‐recognition, or treatment barriers may be more influential [109, 110]. Pooling these pathways under a single ethnic label risks biologic oversimplification and may obscure the most modifiable determinants of underdiagnosis.

Overall, contemporary epidemiologic evidence indicates that OSA is common and increasing worldwide, but diagnostic definitions, persistent underdiagnosis, measurement strategy, and structural inequities strongly shape its apparent distribution. Aging, obesity, and sex‐specific physiological states influence risk trajectories, while marked disparities remain across populations. The especially high prevalence of OSA in fibrotic diseases—particularly IPF and other fibrotic ILD—supports targeted screening in these groups and provides a clinically relevant substrate for the OSA–fibrosis interactions developed in subsequent sections [22, 103]. More broadly, the available evidence supports context‐sensitive diagnostic strategies, including careful modality selection, phenotype‐specific indices, and equity‐oriented care pathways that directly address structural barriers to testing and long‐term treatment adherence [90, 93].

In summary, contemporary OSA epidemiology is more accurately characterized as phenotype‐weighted rather than purely count based. Populations with a higher prevalence of REM‐predominant disease, increased hypoxic or ventilatory burden, women‐specific symptom profiles, or significant fibrotic and metabolic comorbidity may experience disproportionate downstream risk, even when conventional AHI‐based prevalence rates appear similar [60, 111].

Table 1 summarizes the principal epidemiologic modifiers, measurement‐related determinants, and comorbidity‐enriched contexts that shape OSA burden and diagnostic interpretation.

**TABLE 1:** Epidemiologic architecture of obstructive sleep apnea: burden, modifiers, endotypes, and comorbidity‐enriched contexts.

Domain	Key synthesis	Representative quantitative/phenotypic signal	Interpretive implication
Global burden and diagnostic architecture	OSA burden is strongly shaped by the scoring rule and recording platform: respiratory event index can underestimate severity relative to the apnea–hypopnea index, and modified scoring criteria can materially alter HSAT‐based classification [34, 35]. Multinight assessment further shows that single‐night sampling can miss intermittently expressed disease [37].	A landmark modeling analysis estimated that about 936 million adults aged 30–69 years have OSA (AHI ≥5/h) and about 425 million have at least moderate disease [1]. Use of AHI3% rather than AHI4 substantially increases the diagnostic yield [33].	Prevalence should therefore be interpreted as a moving target generated by biology, technology, and policy, with additional growth projected as populations age and obesity increases [46].
Age	Objective burden generally rises with age, but age effects are method dependent and should not be reduced to a simple linear accumulation of events [47, 48, 49].	Clinically relevant OSA is not restricted to older adults: pooled prevalence is about 16% in adults aged 18–30 years [52], and about 35.9% in older adults [53]. In children, moderate OSA is often around 1–2% [54], with estimates increasing when contemporary diagnostic frameworks are used [56].	In older adults, higher AHI may coexist with less prominent classic sleepiness, indicating a partial decoupling between physiological burden and symptom perception and limiting symptom‐based case finding [50, 51].
Sex and female‐specific physiology	OSA remains more prevalent in men, but the sex gap narrows with aging and after menopause [38, 50, 59, 60, 61]. In women, insomnia, fatigue, poor sleep quality, and fragmented sleep often dominate over classic daytime sleepiness or witnessed apneas [38, 50, 59, 60, 61].	Women are more likely to exhibit REM‐predominant disease despite a lower total AHI [63]. Women may also present with flow‐limited breathing phenotypes [64]. Pregnancy can unmask or worsen OSA, with prevalence rising from 10.5% in early pregnancy to 26.7% in late gestation [70].	Sex‐neutral, AHI‐centric screening frameworks are therefore prone to underrecognizing female OSA, especially around menopause and in REM‐enriched phenotypes [57, 58, 60, 65].
Adiposity, airway anatomy and endotypes	Obesity is a dominant but nonexclusive driver of OSA [50, 73, 74, 76, 77, 78]. BMI is only a crude surrogate for anatomically relevant traits, including tongue volume, pharyngeal length, regional soft‐tissue loading, reduced lung volume, and impaired neuromuscular compensation [50, 73, 74, 76, 77, 78].	Body‐weight change shows a clear dose–response: a 10% weight gain predicts about a 32% increase in AHI, whereas a 10% weight loss predicts about a 26% reduction [80]. Weight loss also reduces tongue fat [83]. Tirzepatide lowers AHI and hypoxic burden in obesity‐associated OSA [84].	The obesity–OSA relationship should therefore be interpreted through airway anatomy plus endotypes rather than BMI alone, because clinically important nonobese OSA remains common [81, 85].
Race/ethnicity, social determinants and measurement bias	Disparities in OSA reflect structural determinants spanning access to testing, treatment uptake, and the measurement architecture used to define disease [87, 88, 93, 94]. Biological and genetic influences also contribute [108].	After diagnosis, inequity persists: Hispanic adults with OSA had lower adjusted odds of receiving CPAP in the All of Us cohort [91]. In diverse home‐testing cohorts, Black participants, especially Black women, showed higher REM‐stage REI despite similar NREM indices [92].	Between‐group prevalence comparisons should therefore be interpreted cautiously, because apparent differences may reflect hidden REM‐stage burden, device algorithms, delayed recognition, or barriers to long‐term treatment implementation [23, 89, 92].
Comorbidity‐enriched populations	OSA is highly prevalent in several chronic disease populations relevant to downstream fibrosis and metabolic injury, including CKD [98], IPF [103], and MASLD [23, 26].	In CKD, the pooled prevalence is about 39% [100]. OSA is common in IPF and also present in non‐IPF fibrotic ILD [22, 104]. MASLD‐focused literature also explicitly recognizes OSA as a relevant comorbidity [26].	These settings deserve priority for case finding and for phenotype‐weighted interpretation of risk, because conventional event counts may underestimate clinically important burden in patients enriched for fibrotic or metabolic comorbidity [60, 111].

This table summarizes the principal domains that shape the epidemiology and clinical interpretation of obstructive sleep apnea (OSA), including diagnostic architecture, age, sex‐specific physiology, adiposity and airway endotypes, race/ethnicity and social determinants, and comorbidity‐enriched populations. For each domain, it integrates the key conceptual synthesis with representative quantitative or phenotypic signals and the corresponding interpretive implications. Collectively, the table highlights that OSA prevalence, recognition, and apparent severity are not fixed constructs, but emerge from the interaction between biological susceptibility, demographic and sex‐related modifiers, measurement frameworks, and clinical contexts enriched for downstream metabolic or fibrotic risk.

*Abbreviations*: AHI, apnea–hypopnea index; BMI, body mass index; CKD, chronic kidney disease; CPAP, continuous positive airway pressure; HSAT, home sleep apnea testing; ILD, interstitial lung disease; IPF, idiopathic pulmonary fibrosis; MASLD, metabolic dysfunction‐associated steatotic liver disease; NREM, non‐rapid eye movement; OSA, obstructive sleep apnea; REI, respiratory event index; REM, rapid eye movement.

## 3 Pathophysiological Mechanisms

This mechanistic section aims not only to catalog the causes of upper‐airway collapse but also to clarify why the frequency of respiratory events alone does not fully capture downstream organ risk in OSA. Patients with similar AHI values may experience markedly different combinations of intermittent hypoxemia, event duration, sleep fragmentation, intrathoracic pressure stress, autonomic activation, and endotypic vulnerability, and these differences help determine whether nocturnal breathing disturbance translates into predominantly symptomatic disease or into broader cardiovascular, metabolic, neurocognitive, and fibro‐inflammatory injury. The following mechanistic framework offers a biological rationale for advancing from an event‐count model to an interpretation of OSA severity based on phenotype and physiological burden.

**FIGURE 1:**
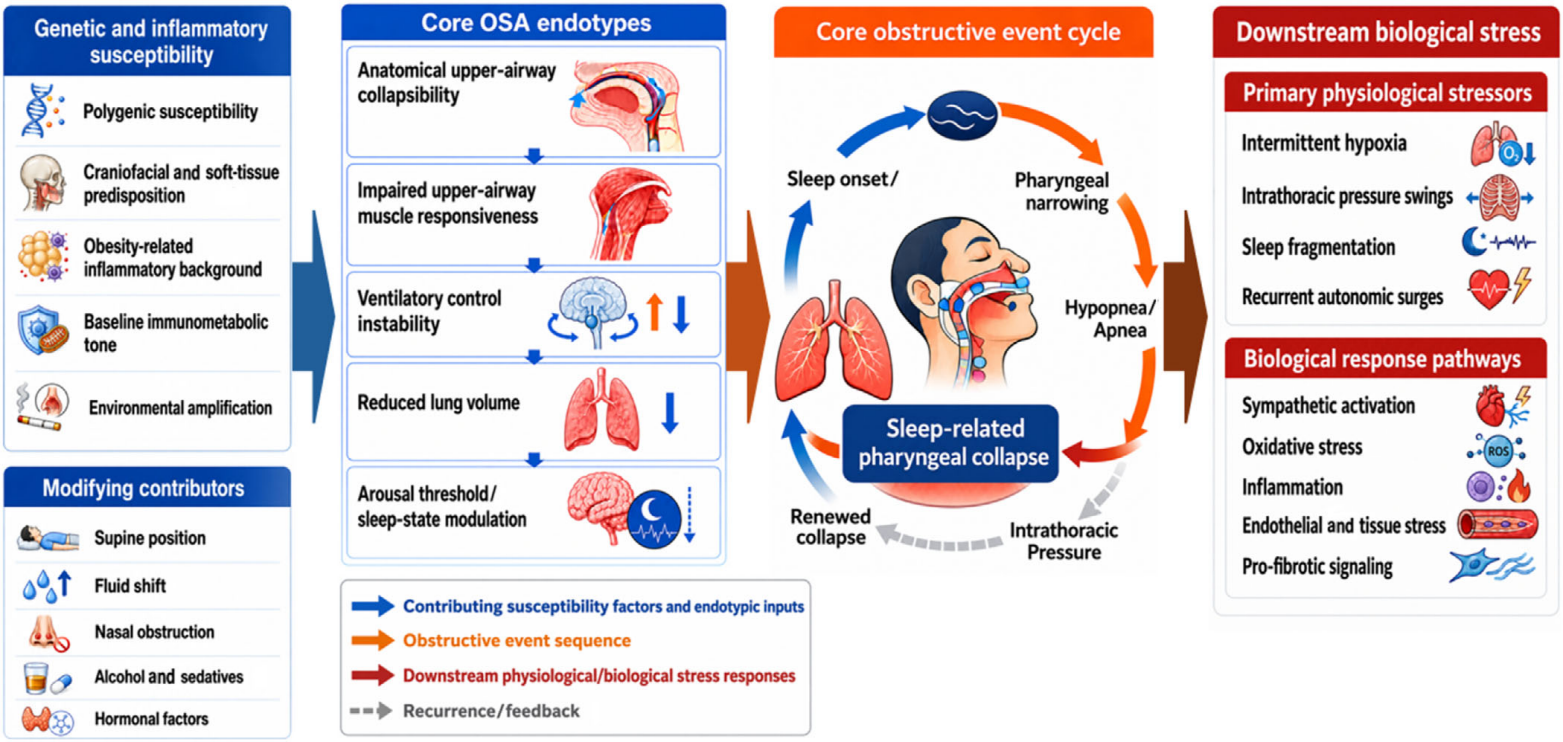
Endotype‐based pathophysiological architecture of obstructive sleep apnea. This figure summarizes the contemporary mechanistic framework by which OSA arises from interactions among baseline susceptibility factors, modifying contributors, core pathophysiological endotypes, recurrent obstructive event cycling, and downstream biological stress. Polygenic susceptibility, craniofacial and soft‐tissue predisposition, obesity‐related inflammatory background, baseline immunometabolic tone, environmental amplification, sleep position, fluid shift, nasal obstruction, alcohol/sedative exposure, and hormonal factors shape individual vulnerability to OSA. These contributors converge on the principal endotypes that govern disease expression, including anatomical upper‐airway collapsibility, impaired upper‐airway muscle responsiveness, ventilatory control instability, reduced lung volume with loss of caudal traction, and arousal‐threshold/sleep‐state modulation. Their interaction promotes a recurrent obstructive event cycle in which sleep‐related reductions in airway tone favor pharyngeal narrowing, hypopnea/apnea, intrathoracic pressure stress, and renewed collapse. Repetitive obstructive events generate key physiological stressors, including intermittent hypoxia, intrathoracic pressure swings, sleep fragmentation, and recurrent autonomic surges, which activate sympathetic, oxidative, inflammatory, endothelial/tissue‐stress, and profibrotic signaling pathways. Together, these interacting domains help explain the heterogeneity of OSA expression and provide a mechanistic bridge linking endotype composition to systemic and organ‐specific complications. Blue arrows indicate contributing susceptibility factors and endotypic inputs; orange arrows indicate the obstructive event sequence; red arrows indicate downstream physiological and biological stress responses; dashed gray arrows indicate recurrence and feedback. *Abbreviations*: AHI, apnea–hypopnea index; IH, intermittent hypoxia; OSA, obstructive sleep apnea; REM, rapid eye movement.

Within this framework, OSA should be conceptualized as a heterogeneous disorder generated by the interaction between anatomical upper‐airway vulnerability and nonanatomical endotypes, chiefly upper‐airway dilator muscle responsiveness, ventilatory control stability (loop gain), arousal threshold, and lung‐volume‐related mechanics. This multidimensional framework explains why patients with apparently similar AHI values can exhibit very different combinations of desaturation depth, event duration, autonomic activation, and downstream cardiometabolic or fibro‐inflammatory stress [112, 113]. Recent translational reviews further emphasize that OSA expression is additionally modulated by sex, age, obesity phenotype, sleep stage, body position, and coexisting inflammatory or cardiometabolic states, supporting the shift from a purely event‐count model toward mechanism‐based phenotyping [60, 114]. The increasing practical value of this framework is that all four core endotypes can now be estimated from standard PSG with acceptable reproducibility in moderate‐to‐severe disease, making mechanism‐based phenotyping increasingly feasible outside specialist physiology laboratories. Large contemporary datasets further suggest that age‐ and sex‐related worsening does not occur uniformly across traits, but rather through different combinations of collapsibility, loop gain, arousability, and muscle compensation [115, 116]. This integrated pathophysiological framework is summarized in Figure 1.

An additional point emerging from repeat PSG work is that OSA endotypes are measurable but not perfectly fixed at the individual level. In a 2025 repeated‐PSG analysis, AHI, flow‐limitation frequency, and several endotypic traits—particularly Vactive, Vpassive, Vcomp, and arousal threshold in highly variable patients—showed meaningful night‐to‐night variability. In contrast, earlier reproducibility studies still support the acceptable reliability of PSG‐derived endotyping in moderate‐to‐severe OSA. Accordingly, a single‐night estimate is clinically useful. However, mechanism‐based phenotyping should increasingly be interpreted as a probabilistic characterization that may benefit from repeat assessment when disease expression appears context‐sensitive or when treatment decisions are finely balanced [116, 117].

### 3.1 The Obstructive Event as a Dynamic Cardiorespiratory Cycle

The core pathophysiological event in OSA is recurrent sleep‐state‐dependent pharyngeal narrowing or collapse that occurs when inspiratory collapsing forces exceed the stabilizing effects of airway anatomy, neuromuscular activation, and caudal traction. In contemporary models, this event is seen as a dynamic cycle rather than a static obstruction: sleep onset or sleep deepening reduces wakefulness‐related airway support; airflow limitation develops; respiratory effort rises; intermittent hypoxemia and hypercapnia accumulate; autonomic activation intensifies; and the airway finally reopens, often with a ventilatory overshoot that predisposes to the next event [14, 114]. The major endotypic inputs and the recurrent obstructive event cycle are schematically illustrated in Figure 1. This repeated obstruction–effort–reopening sequence has consequences that extend far beyond airflow cessation. Negative intrathoracic pressure swings and recurrent sympathetic surges acutely increase cardiovascular load, while event‐related hypoxia/reoxygenation and sleep fragmentation promote oxidative, inflammatory, endothelial, and neurohumoral stress. Accordingly, modern reviews argue that the biological burden of OSA depends not only on how often events occur, but also on the physiologic intensity of each event [14, 85]. Consequently, AHI alone is increasingly regarded as an incomplete descriptor of disease severity. Recent work supports the complementary use of metrics such as hypoxic burden, ventilatory burden, and sleep apnea‐specific heart‐rate response, which more closely capture event depth, duration, ventilatory deficit, and autonomic reactivity. In various contexts, these measures demonstrate a closer pathophysiological association with cardiovascular risk than simple event frequency [8, 118]. Ventilatory burden has also been associated with cardiovascular and all‐cause mortality, while pulse‐oximetry‐derived event‐related heart‐rate response is emerging as a scalable marker of cardiovascular susceptibility and potential PAP benefit [7, 119]. Recent human cohort data also suggest that different physiological‐burden metrics may map onto different downstream vulnerabilities rather than reflecting a single interchangeable construct. In the multiethnic study of atherosclerosis (MESA), hypoxic burden—but not ventilatory burden, arousal burden, or conventional event‐frequency metrics—was associated with greater white matter hyperintensity volume, supporting the view that the biological meaning of an obstructive event depends on which component of the event cycle dominates tissue stress [118, 120]. Recent neuroimaging‐based cohort studies indicate that both the timing and stage distribution of hypoxemia have biological significance. In addition to overall hypoxic burden, REM‐related hypoxemia has been associated with increased frontal and parietal white‐matter hyperintensity, reduced entorhinal cortex thickness, and impaired sleep‐dependent memory discrimination in older adults. These findings support the hypothesis that nocturnal injury in OSA may be exacerbated when deeper oxygen desaturations occur during physiological states characterized by reduced upper‐airway muscle tone [121]. This complements broader cohort evidence that hypoxic burden, more than ventilatory or arousal burden, tracks white‐matter injury on MRI [120]. A further implication is that respiratory event morphology itself likely contains mechanistic information beyond raw event counts. In a 2026 retrospective study, greater mean apnea–hypopnea duration was associated with lower minimum nocturnal oxygen saturation and longer time spent below 88% saturation despite limited correlation with AHI, indicating that event duration may encode how anatomy, arousability, and ventilatory control interact during each obstructive cycle and should be considered in phenotyping rather than dismissed as a secondary PSG descriptor [122].

Human experimental work using the Mueller maneuver further supports the hemodynamic and arrhythmogenic relevance of event‐related negative intrathoracic pressure swings. These studies show acute reductions in left‐atrial volume and left‐ventricular systolic performance, worsening of functional mitral regurgitation in susceptible hearts, and promotion of atrial ectopy and repolarization abnormalities under simulated obstructive loading [123, 124, 125].

A complementary mechanistic lens is the Starling‐resistor model of a collapsible pharyngeal segment, in which progressive reductions in intraluminal pressure relative to surrounding tissue pressure move the airway from inspiratory flow limitation to complete occlusion. In this framework, Pcrit remains the canonical index of intrinsic mechanical vulnerability and helps unify snoring, hypopnea, and frank apnea within a single pressure‐flow continuum [126, 127].

Recent repeat‐measurement data further suggest that physiological‐burden descriptors and event morphology should not be viewed as fully interchangeable surrogates of the same latent process. Patients with large night‐to‐night AHI shifts also exhibit variability in flow‐limited breathing frequency and in multiple endotypic measures, implying that the biological meaning of a given event count partly depends on how collapsibility, compensation, and arousability are expressed on that particular night rather than on event frequency alone [117, 118].

### 3.2 Anatomical Susceptibility: From Structural Crowding to Site‐Specific Collapse

Anatomical susceptibility remains the most intuitive substrate for OSA, but it should be interpreted as a spectrum of mechanical vulnerability rather than a single structural defect. A highly collapsible airway reflects the combined effects of a relatively small bony enclosure, excess soft‐tissue load, increased lateral wall compliance, upper‐airway length, and reduced structural stiffness. Contemporary endotype‐focused reviews continue to frame this mechanical vulnerability through the concept of Pcrit, while also stressing that clinic‐scalable surrogates of collapsibility are becoming increasingly feasible [85, 114]. Obesity contributes importantly to this anatomical load, but recent imaging work indicates that obese and nonobese OSA do not simply differ by “more versus less tissue.” In moderate‐to‐severe OSA, higher BMI is associated with larger soft‐tissue volumes throughout the pharynx, including tongue, tongue fat, soft palate, lateral walls, fat pads, and epiglottic structures. On the other hand, lower‐BMI OSA is more often characterized by craniofacial restriction and retrognathic traits. Thus, different anatomic pathways can converge on a similarly crowded airway [128, 129]. A 2025 systematic review/meta‐analysis further supports tongue volume and tongue adiposity as clinically relevant anatomical correlates of OSA [130]. The strong interest in tongue fat is mechanistically justified because weight loss does not merely reduce global adiposity; it can remodel the upper airway itself. A landmark MRI study showed that reductions in tongue fat significantly mediated improvements in OSA severity after weight loss, establishing tongue fat as a modifiable anatomical target rather than just an incidental correlate of obesity [83]. Anatomical vulnerability is also site specific. Contemporary drug‐induced sleep endoscopy (DISE) and manometry studies confirm that collapse may predominate at the velum, lateral pharyngeal walls, tongue base, or epiglottis, and that multilevel collapse is common in more severe disease. Complete concentric palatal collapse remains particularly relevant because it influences candidacy for HNS and other non‐PAP approaches. Nevertheless, recent large‐series data show that BMI alone predicts this pattern only imperfectly [131, 132]. Epiglottic collapse is increasingly recognized as a clinically important phenotype, and recent data suggest that nasal airflow patterns from home sleep testing may help infer this trait beyond specialized endoscopy [133]. Recent anatomical series also suggest that site‐specific collapse is prognostically meaningful rather than merely descriptive. In patients selected for soft palatal webbing flap pharyngoplasty, stronger palatal and lateral‐wall disease favored response, whereas preoperative epiglottic collapsibility signaled less favorable outcomes, indicating that different anatomical drivers can require fundamentally different interventions [132, 134]. Additional DISE work indicates that positional anatomy is especially relevant at the tongue base: shifting from supine to lateral positioning can markedly reduce tongue‐base obstruction in many patients, highlighting that an apparently fixed anatomic phenotype may actually be strongly gravity and mechanics dependent [135, 136]. At the same time, current DISE classifications show only fair‐to‐moderate interobserver agreement. Hence, anatomical phenotyping is most robust when endoscopy is interpreted alongside imaging, manometry, and PSG‐derived physiology rather than as a stand‐alone arbiter of mechanism [131, 137]. Biomechanical work also suggests that upper‐airway anatomy should not be considered only in terms of where collapse occurs, but also in terms of how the segment fails under changing pressure. Quantitative DISE‐pressure studies identify distinct velopharyngeal tube‐law phenotypes, including inspiratory collapse, expiratory palatal prolapse, and relatively stable airways that collapse only when support pressure falls, indicating that superficially similar palatal obstruction can reflect different mechanical behaviors [138]. In parallel, recent DISE work indicates that lateral epiglottic collapse is strongly associated with laterally directed pharyngeal collapse and complete concentric palatal collapse, supporting the view that site‐specific obstruction patterns may be biomechanically linked rather than independent findings [139]. The convergence between structural and functional phenotyping is also becoming clearer. In an observational DISE‐PSG study, complete concentric palatal collapse was independently linked to greater collapsibility and lower arousal threshold, lateral wall collapse to poorer muscle compensation, and epiglottic collapse to greater compensation, indicating that the site of collapse increasingly maps onto distinct physiological liabilities rather than serving only as a descriptive anatomic label [140]. State‐dependent imaging strengthens this interpretation, as wake‐to‐sleep MRI demonstrates that, in contrast to controls, apneic subjects develop retroglossal narrowing, posterior displacement of the soft palate, and medial–lateral wall motion in all tongue octants, as well as the mandible. These findings indicate that sleep‐related airway collapse results from coordinated soft‐tissue and craniofacial shifts rather than from fixed anatomical crowding alone [141].

An additional mechanistic distinction that remains useful in contemporary phenotyping is the separation between passive and active collapsibility: passive Pcrit approximates anatomy‐dominated vulnerability, whereas the active response incorporates compensatory dilator‐muscle recruitment as ventilatory drive rises. Recent translational studies indicate that wake‐based negative expiratory pressure testing may serve as a scalable surrogate for assessing pharyngeal collapsibility when formal sleep Pcrit measurements are not feasible. Nevertheless, further validation is necessary prior to widespread clinical implementation [142].

A further implication is that anatomy‐targeted interventions do not behave as purely “structural” therapies. In a 2025 PSG‐endotyping study of multilevel upper‐airway surgery, successful intervention increased Vpassive, reduced loop gain, and shifted arousal threshold, whereas baseline lower loop gain independently favored response. These findings suggest that structural remodeling can secondarily reshape nonanatomical traits, helping explain why anatomically similar patients may diverge in outcomes after surgery or device‐based therapies [140, 143].

Prospective DISE under mandibular advancement further indicates that anatomy‐targeted therapy does not modify collapse uniformly across all levels: with the device set at 75% of maximal protrusion, collapse degree decreased at the palate, oropharynx, tongue base, and hypopharynx, but not at the epiglottis, reinforcing the concept that apparently “anatomical” OSA often reflects a multilevel but mechanically asymmetric phenotype rather than a single obstructing segment [144].

### 3.3 Neuromuscular Responsiveness and Dynamic Airway Compensation

Anatomical load alone does not determine whether the airway actually obstructs during sleep. Upper‐airway patency depends critically on the ability of dilator muscles, especially the genioglossus and other tongue/palatal stabilizers, to respond to rising negative pressure and ventilatory drive. The transition from wakefulness to sleep is a particularly vulnerable interval because wakefulness‐related excitatory input to these muscles falls abruptly, exposing the underlying anatomical load before compensatory recruitment has fully engaged [14, 114]. In endotype terms, “muscle responsiveness” or “airway compensation” describes how effectively the upper airway motor output increases as obstruction progresses. Patients with preserved compensation may partially offset marked anatomical vulnerability, whereas those with poor compensation develop persistent obstruction despite strong inspiratory effort. This principle also helps explain why REM sleep is often a period of heightened susceptibility, as reduced tonic motor output and unstable phasic recruitment may further impair airway defense [60, 112]. The translational relevance of this trait is now clear. Hypoglossal nerve stimulation (HNS) directly augments upper‐airway dilator activation and has consolidated its role as a mechanism‐based therapy for selected adults intolerant of PAP. An updated 2024 meta‐analysis supports HNS as an effective intervention with favorable adherence and symptom outcomes, while also underscoring the importance of refined phenotypic selection [145]. Pharmacologic strategies targeting neuromuscular control are also advancing. Noradrenergic–antimuscarinic combinations such as aroxybutynin/atomoxetine (AD109) and multimechanism regimens combining atomoxetine–oxybutynin with acetazolamide have produced clinically meaningful reductions in OSA severity in randomized trials, reinforcing the principle that pharmacotherapy can improve airway stability when matched to underlying physiology rather than prescribed empirically [146, 147]. Another underappreciated layer of neuromuscular vulnerability is pharyngeal sensory‐motor injury. A systematic review of palate and uvular neurophysiology reported worsening sensory dysfunction, neural abnormalities, and histological features of neuropathy with increasing snoring and OSA severity, consistent with the idea that repetitive vibration and tissue trauma may progressively blunt reflex airway defense and dilator effectiveness over time [148, 149]. Mechanistic drug studies further show that neuromuscular and non‐neuromuscular traits are tightly coupled rather than isolated. In a 1‐week randomized crossover trial, pimavanserin plus atomoxetine reduced AHI while improving pharyngeal collapsibility and lowering loop gain without materially changing arousal threshold, indicating that successful neuromodulation may operate through simultaneous effects on airway mechanics and respiratory control rather than by a single muscle‐only pathway [150]. This interpretation is consistent with earlier detailed endotyping work showing that atomoxetine–oxybutynin increased Vpassive and muscle compensation while also lowering loop gain, thereby highlighting the interdependence between dilator recruitment and the broader instability of the obstructive cycle [151]. Emerging stimulation and behavioral data further indicate that neuromuscular vulnerability extends beyond genioglossal activation alone. Structure‐based analyses of ansa cervicalis stimulation showed meaningful reductions in both pharyngeal closing and opening pressures across all major flow‐limiting structures, with particularly strong effects on palatal, lateral‐wall, and epiglottic collapse, underscoring the importance of caudal stabilizing forces generated by infrahyoid musculature. In parallel, contemporary exercise‐endotype work suggests that regular exercise may preferentially benefit patients with poor upper‐airway muscle function and higher arousal threshold, reinforcing the concept that neuromuscular endotypes are at least partly modifiable rather than fixed traits [152, 153].

Noninvasive imaging data further support the hypothesis that neuromuscular vulnerability can be characterized both biomechanically and functionally. Shear‐wave elastography has identified higher genioglossus and geniohyoid stiffness in individuals with OSA than in controls, despite similar muscle thickness. Notably, geniohyoid stiffness increased with disease severity, suggesting that altered material properties of extrinsic tongue muscles may contribute to impaired dynamic airway stabilization even when gross morphology appears unchanged [154]. Computational fluid dynamics‐MRI modeling offers additional mechanistic insight. Pressure‐acceleration analysis at the soft palate, tongue, and epiglottis indicates that airway collapse may initiate prior to reaching peak negative intraluminal pressure. Substantial neuromuscular‐driven motion persists during exhalation, supporting the hypothesis that neuromuscular failure in OSA results from abnormal timing and force balance throughout the respiratory cycle, rather than solely from inspiratory hypotonia [155].

A particularly vulnerable interval is sleep onset, when withdrawal of wakefulness‐related excitatory input abruptly unmasks underlying anatomical load before compensatory recruitment has fully engaged. Over longer time scales, repetitive snoring‐related vibration and tissue trauma may contribute to sensory‐motor injury of the soft palate and pharyngeal dilators, thereby weakening reflex airway defense and making neuromuscular failure partly acquired rather than purely constitutional [126, 149].

Importantly, the functional phenotype observed during drug‐induced sleep is not physiologically neutral. In a 2025 randomized crossover trial, both dexmedetomidine and propofol worsened pharyngeal collapsibility. However, propofol additionally reduced upper‐airway gain, raised loop gain, increased NREM AHI, and lowered nadir SpO_2_, whereas dexmedetomidine exerted a smaller endotypic perturbation. These data are directly relevant when interpreting DISE‐based or sedative‐assisted phenotyping, because the sedative itself may amplify the very instability being measured [131, 156].

### 3.4 Ventilatory Control Instability and CO_2_ Reserve

Ventilatory control instability—commonly described as high loop gain—is a major nonanatomical contributor to OSA in a substantial subset of patients. In this context, the respiratory control system overreacts to relatively small disturbances in ventilation, causing oscillations in drive that amplify rather than dampen the consequences of obstruction. High loop gain can therefore convert modest anatomical compromise into repetitive cycles of obstruction, hyperventilation, hypocapnia, and renewed vulnerability to collapse [112, 157]. A closely linked concept is the CO_2_ reserve, namely, the distance between eupneic PaCO_2_ and the apneic threshold during sleep. When this reserve is narrow, even a brief postevent ventilatory overshoot can lower PaCO_2_ sufficiently to suppress central drive, reduce upper‐airway muscle activation, and facilitate recurrent obstruction or mixed event patterns. Contemporary reviews stress that loop gain and CO_2_ reserve are not static traits: they vary with sleep stage, arousal pattern, background chemosensitivity, obesity‐related mechanics, and comorbid cardiopulmonary disease [114, 157]. Loop gain has become increasingly relevant in clinical practice because it can now be estimated, although imperfectly, from standard PSG. This advancement enables large‐scale endotyping, which was previously less practical. These developments have significant therapeutic implications. Specifically, interventions that reduce chemosensitivity or stabilize blood‐gas control are most appropriate for patients whose OSA is driven by ventilatory overshoot and undershoot rather than by anatomical factors alone [85, 112]. In this regard, the current pharmacologic literature supports this logic. In randomized studies, combination therapy with acetazolamide plus atomoxetine–oxybutynin improved OSA severity by targeting complementary mechanisms, while the 2025 FLOW Phase‐2 trial showed dose‐dependent efficacy of sulthiame, a carbonic‐anhydrase inhibitor that appears to improve ventilatory control and upper‐airway stability simultaneously [146, 158]. High loop gain is also not fixed across the night or circadian cycle. Controlled physiology studies demonstrated higher loop gain and arousal threshold in the morning than in the evening or afternoon, suggesting that chemoreflex instability can be rhythmically modulated and may partly explain temporal variability in event expression even within the same individual [159]. Consistent with the concept that loop gain interacts with anatomy rather than acting alone, a 2026 randomized trial showed that combining supplemental oxygen with a mandibular advancement device (MAD) reduced AHI more than the oral appliance alone, with the largest effects in patients with both higher loop gain and greater collapsibility [160].

Recent pharmacologic endotyping work further refines this concept by showing that multitarget regimens may act through different dominant mechanisms depending on dose and baseline physiology. In a 2025 randomized analysis of acetazolamide‐dronabinol, medium and high doses reduced loop gain in a dose‐dependent manner, whereas the low‐dose condition mainly improved collapsibility; moreover, higher baseline loop gain and greater collapsibility predicted treatment response. These findings support the increasingly important principle that “pharmacotherapy for OSA” is not a unitary category, but a physiology‐matched strategy whose dominant mechanistic target can shift across regimens and doses [146, 161].

Another important implication is that the loop gain should be decomposed into controller and plant gains rather than treated as a single, undifferentiated quantity. In a 2025 randomized flupirtine trial designed to attenuate controller gain via KCNQ‐channel activation, no overall reduction in loop gain or OSA severity was observed, underscoring that controller‐gain targeting may be more difficult to translate clinically than plant‐gain modulation and that apparent “high loop gain” may conceal mechanistically different unstable‐control phenotypes [162].

This physiology‐treatment linkage is echoed by recent oral‐appliance data: clinically available markers of severe collapsibility, high loop gain, and low arousal threshold clustered in nonresponders after titration, indicating that mandibular advancement is less likely to succeed when ventilatory instability and unfavorable arousal dynamics remain substantial alongside anatomical load [163].

### 3.5 Arousal Threshold, Sleep Stage, and Event Termination

The respiratory arousal threshold determines the amount of respiratory effort or chemical drive that must accumulate before sleep is interrupted during an obstructive event. This trait is physiologically ambivalent. On one hand, arousal can restore airway patency and ventilation; on the other, it fragments sleep, triggers sympathetic activation, and may provoke the ventilatory overshoot that destabilizes subsequent breathing. Thus, arousal is not merely a rescue mechanism—it can also be a driver of cyclical instability [85, 112]. A low arousal threshold tends to produce short, frequent events with prominent sleep fragmentation, because patients awaken before chemical drive and upper‐airway motor recruitment have had time to stabilize the airway. Conversely, a high threshold may facilitate increased dilator muscle recruitment and event termination without cortical arousal in certain individuals, whereas in others it can prolong airway obstruction, exacerbate oxygen desaturation, and increase intrathoracic pressure stress. The clinical significance of this balance helps explain why event duration and physiological intensity may carry prognostic information not captured by the AHI alone [114, 118]. Arousability is also stage and sex dependent. REM‐predominant OSA, lower non‐REM AHI with greater REM susceptibility, and lower arousal thresholds in women are increasingly recognized features of sex‐specific pathophysiology, especially after menopause, when OSA prevalence approaches that observed in men [60]. Therapeutically, hypnotic manipulation of arousal threshold remains promising but clearly phenotype dependent. A 2024 systematic review and meta‐analysis showed that hypnotics reliably raise arousal threshold. Nevertheless, their average impact on OSA severity is small and heterogeneous, supporting their use only in carefully selected endotypes and not as indiscriminate stand‐alone therapy [164]. This phenotype dependence is clinically important because manipulating sleep continuity does not automatically improve the specific arousal‐pathophysiology that sustains OSA. In a 2025 crossover trial, short‐term zolpidem did not improve CPAP adherence during acclimatization, underscoring that raising arousal threshold or facilitating sleep initiation is not equivalent to correcting the obstructive mechanism itself [164, 165]. Noninvasive phenotyping studies indicate that REM‐predominant OSA is primarily characterized by increased pharyngeal collapsibility during REM sleep. In contrast, non‐REM‐predominant OSA is more closely associated with elevated loop gain during NREM sleep, demonstrating that sleep stage is not merely a contextual modifier but can identify the dominant endotype in a given patient [166]. Furthermore, direct physiological measurements indicate that REM vulnerability cannot be attributed solely to a general increase in pharyngeal collapsibility. In a mechanistic study using calibrated diaphragm EMG and genioglossus recordings, REM‐related worsening was driven predominantly by withdrawal of ventilatory drive, particularly during phasic REM, rather than by a uniform further fall in pharyngeal responsiveness; this finding refines interpretation of REM‐predominant OSA and highlights stage‐specific control of both pump and upper‐airway motor output [167].

Recent clinical data also strengthen the argument that REM‐predominant OSA should not be dismissed when the overall AHI is only mild to moderate. In a 2025 cohort, patients with mild REM‐OSA were about twice as likely to report EDS as those with comparable NREM‐OSA, despite limited differences in broader mood or fatigue scales. These observations reinforce the idea that stage‐specific vulnerability can carry disproportionate symptomatic relevance and may justify lower treatment thresholds in selected symptomatic REM‐predominant phenotypes [167, 168].

Recent work also indicates that the arousal phenotype cannot be captured adequately by arousal frequency alone. In a 2025 ORP‐based analysis of untreated OSA, arousal intensity increased with the duration of the preceding event, was greater after apneas than after hypopneas, and was attenuated in deeper sleep and in lateral posture; baseline arousal intensity was also independently associated with subjective daytime sleepiness. These data support the idea that how forcefully sleep is disrupted may matter biologically alongside how often, and they strengthen the rationale for incorporating continuous EEG‐derived sleep‐depth metrics into OSA phenotyping when available [169]. Recent translational work also suggests that arousal‐threshold phenotyping has prognostic relevance beyond event termination itself. In a secondary analysis of a sham‐controlled CPAP trial, a higher baseline arousal threshold was associated with greater improvement in executive function after therapy. In contrast, large‐dataset validation studies support ORP as a clinically scalable continuous sleep‐depth metric. Together, these data argue that sleep‐depth/arousability measures may help predict not only event morphology but also which patients derive downstream neurocognitive benefit from treatment [170, 171].

### 3.6 Lung Volume, Caudal Traction, and Respiratory Mechanics

Upper‐airway patency is mechanically coupled to lung volume. As end‐expiratory lung volume falls during sleep, in the supine posture, or with obesity‐related restriction, caudal traction on the trachea and surrounding soft tissues declines, reducing longitudinal tension and making the pharynx more collapsible. Contemporary pathophysiological syntheses continue to emphasize this “tracheal tug” mechanism as an important bridge between thoraco‐pulmonary mechanics and upper‐airway stability [14, 114]. Recent work suggests that this relationship may be especially relevant in obesity, where reduced lung volume and altered chest wall mechanics coexist with soft‐tissue airway loading. In an MRI/endotype study from the SLIM‐OSA program, upper‐airway length was strongly associated not only with AHI but also with hypoxic and ventilatory burden, highlighting that respiratory mechanics and anatomy interact to influence both event generation and event severity [128]. These interactions help explain why the same patient may worsen in supine sleep or during REM, and why interventions that modestly improve respiratory mechanics can reduce OSA severity in selected phenotypes. A 2025 multicenter observational study found that head‐of‐bed elevation significantly reduced AHI and ODI relative to flat supine posture, supporting the view that improved lung volume, reduced rostral fluid shift, and altered gravitational loading can all shift the airway toward greater stability [135].

Direct human evidence also supports the caudal‐traction concept. Dynamic MRI demonstrates that inspiratory caudal tracheal displacement is greater and peaks earlier in individuals with more severe OSA. At the same time, recent mechanistic reviews reinforce that end‐expiratory lung volume is a genuine contributor to upper‐airway stability rather than merely a contextual correlate of anatomy [172, 173].

From a translational standpoint, lung‐volume mechanics should be viewed as a modifiable amplifier of upper‐airway stability rather than a passive background variable. Contemporary mechanical syntheses indicate that end‐expiratory lung volume is continuously influenced by body position, sleep stage, obesity, and chronic lung disease, thereby altering caudal traction across the night; this framework helps explain why relatively small mechanical interventions, such as posture optimization or head‐of‐bed elevation, can yield clinically relevant reductions in event severity in selected phenotypes [135, 172].

Mechanics also help explain why event counts and hypoxemic burden may dissociate in chronic lung disease. In the 2025 SNOOzzzE cohort, patients with COPD‐OSA overlap had lower upper‐airway collapsibility and lower AHI/hypoxic burden than BMI‐matched OSA‐only patients, yet markedly higher T90; hyperinflation and air trapping were inversely associated with AHI and hypoxic burden. These data suggest that increased lung volume can partially stabilize the pharynx even as impaired gas exchange worsens nocturnal desaturation, meaning that respiratory mechanics may simultaneously blunt the frequency of obstructive events and amplify their physiological consequences [172, 174].

### 3.7 Contextual Modifiers of OSA Expression

Several context‐dependent factors can acutely worsen or mitigate OSA expression by shifting the balance among collapsibility, neuromuscular compensation, arousal threshold, and ventilatory stability. Body position is the clearest example. Positional OSA remains common, especially in milder and moderate disease, because supine sleep promotes posterior displacement of the tongue and soft palate, reduces end‐expiratory lung volume, and increases the mechanical disadvantage of the upper airway. In a recent prospective crossover trial, positional therapy effectively reduced supine sleep and improved OSA metrics, although CPAP remained superior at reducing AHI [135, 175]. Importantly, positional vulnerability is not purely anatomical. In a prospective PSG‐derived endotyping study, supine‐predominant OSA was associated with relatively lower passive collapsibility. However, clearly reduced upper‐airway compensation compared with nonpositional OSA, and the transition from lateral to supine posture produced a larger fall in compensation in the supine‐predominant group. These observations suggest that posture can unmask failure of dynamic airway compensation rather than simply shifting tissue backward [176]. Rostral fluid shift is another clinically important modifier, particularly in patients with heart failure, resistant hypertension, CKD, or marked dependent edema. Nocturnal redistribution of fluid from the legs to the neck can increase peripharyngeal tissue pressure and narrow the airway, thereby aggravating collapsibility even when obesity is not the dominant structural driver. A 2024 systematic review reinforces rostral fluid shift as a meaningful pathomechanism in the interaction between OSA and heart failure, rather than a purely theoretical construct [14, 177]. Nasal obstruction is usually a modifying rather than a primary cause of adult OSA, but it can increase upstream resistance, encourage oral breathing, worsen subjective symptoms, and impair PAP tolerance. Recent evidence remains sobering; however, an updated 2025 meta‐analysis found no clear short‐term improvement in CPAP compliance or global nasal symptoms with topical nasal steroids in unselected OSA populations, indicating that treatment of nasal disease should be individualized rather than assumed to improve OSA automatically [178]. Finally, medications and substances can materially alter OSA severity. Alcohol appears to worsen OSA on average by increasing collapsibility and blunting protective responses. On the other hand, opioid exposure is particularly relevant because it increases the prevalence of central sleep apnea and can compound obstructive sleep‐related breathing instability. A 2026 meta‐analysis confirms higher AHI among alcohol users and higher central sleep apnea prevalence among opioid users, reinforcing the importance of targeted medication and substance review when OSA severity seems disproportionate or unstable over time [96]. Contextual modifiers may also operate over broader time scales than a single posture or a single night. In the Shanghai Sleep Health Study, positional OSA was associated with lower loop gain, lower arousal threshold, reduced collapsibility, and better compensation than nonpositional OSA. Unexpectedly, loop gain emerged as a particularly important determinant of positional‐OSA severity, reinforcing that posture‐related disease cannot be reduced to gravity alone. At the population level, OSA severity varies across seasons and is partially attributable to ambient temperature and changes in sleep duration. These findings indicate that environmental context can influence disease expression even when the underlying endotype substrate remains stable [179, 180].

Environmental context may also act as a short‐horizon modifier of OSA expression. In a large 2025 cross‐sectional analysis, relative humidity, ambient temperature, and PM2.5 exposure were associated with shifts in REM/NREM distribution and with higher arousal indices, with PM2.5 mediating several of these relationships. Although these data are not specific to mechanistic endotyping, they strengthen the concept that nightly OSA severity can be modulated by external environmental stressors that act through sleep architecture and arousability rather than through anatomy alone [179, 181].

The concept of a positional‐mechanical modifier is reinforced by intervention studies using small, clinically feasible posture changes. In a 2025 randomized pilot trial, mild head‐of‐bed elevation implemented with an adjustable bed reduced RDI and AHI without worsening sleep satisfaction, complementing the larger observational HOBE literature and suggesting that simple nocturnal geometry changes can meaningfully shift airway mechanics outside the laboratory. These findings are mechanistically coherent with fluid‐redistribution models, because even modest trunk elevation may simultaneously increase lung volume, lessen rostral fluid displacement, and reduce posterior gravitational loading of pharyngeal structures [177, 182].

### 3.8 Genetic, Inflammatory, and Multiomic Susceptibility

OSA susceptibility is heritable, but not in a simple monogenic sense. Current evidence supports a polygenic architecture in which inherited risk is distributed across pathways linked to obesity, craniofacial structure, upper‐airway control, ventilatory regulation, and cardiometabolic biology. Large‐scale GWAS in the Million Veteran Program showed that the OSA genetic architecture is heterogeneous by sex, while more recent polygenic‐score analyses indicate that OSA‐related genetic liability captures pathways relevant to downstream cardiovascular disease rather than obesity alone [183, 184].

Recent bidirectional Mendelian‐randomization data further suggest that the obesity–OSA relationship is not simply unidirectional: genetic liability to OSA was associated with higher BMI and greater trunk and whole‐body fat mass, whereas BMI and regional fat‐mass traits showed strong causal effects on OSA risk, supporting a feed‐forward interaction between sleep‐disordered breathing and adiposity distribution rather than a one‐way obesity‐to‐OSA model [185].

Inflammatory susceptibility is intertwined with this genetic background. OSA does not simply generate inflammation after disease onset; rather, pre‐existing immunometabolic context may shape who develops disease, how severe it becomes, and which downstream complications dominate. Recent reviews describe OSA as an immune‐modulating disorder in which IH and sleep fragmentation activate HIF‐1α, nuclear factor‐κB (NF‐κB), and NLRP3‐inflammasome signaling, thereby promoting chronic low‐grade inflammation, immune‐cell dysfunction, endothelial injury, and tissue‐remodeling programs [186, 187].

Mendelian randomization and biomarker studies add further support to this framework. Novel analyses of inflammatory proteins and genetic susceptibility markers have identified immune‐related signals that may contribute to OSA risk and heterogeneity, suggesting that inflammatory pathways should be viewed not only as downstream consequences but also as part of the disease‐susceptibility architecture itself [184, 188].

The inflammatory‐protein signal is also becoming more specific at the biomarker level. A 2025 Mendelian‐randomization study identified IL‐18R1, SLAMF1, IL‐10RA, and IL‐17C among the most robust inflammatory candidates associated with OSA‐related phenotypes after multiple‐testing correction, moving the field beyond generic cytokine language toward tractable protein‐level susceptibility markers [188]. Complementing this, a 2024 bidirectional Mendelian‐randomization/mediation analysis linked OSA to selected inflammatory proteins and plasma metabolites, while a 2025 epigenetic study showed that a methylation risk score for CRP—but not CRP polygenic scores—associated with OSA traits, hypertension, and diabetes. Together, these findings suggest that inflammatory susceptibility may be encoded not only in static genotype but also in longer‐horizon molecular exposure signatures that help explain heterogeneous cardiometabolic vulnerability across apparently similar OSA phenotypes [189, 190].

Taken together, modern evidence supports a model in which OSA emerges from the convergence of inherited predisposition, anatomical load, physiological endotypes, environmental modifiers, and immune‐metabolic context. This integrated view is essential for precision medicine because it explains why similar respiratory event counts can arise from different mechanisms and can lead to different patterns of symptoms, organ vulnerability, and treatment response [112, 113].

A further implication of recent genetic and multiomics work is that inherited risk likely extends beyond body habitus alone. Contemporary syntheses indicate that OSA‐associated loci and omics signatures also intersect circadian, inflammatory, obesity‐related, and neuronal pathways, supporting a model in which genetic liability may influence not only who develops OSA but also which downstream biological programs are preferentially engaged once IH and sleep fragmentation begin [183, 191].

Emerging omics work also suggests that susceptibility may be encoded at the pathway level even when single‐gene signals differ across cohorts. A 2025 pooled methylome analysis identified 720 differentially methylated genes in human OSA and found stronger concordance between human and intermittent‐hypoxia animal models at the pathway rather than individual‐gene level, arguing that epigenetic remodeling may converge on reproducible biological programs despite heterogeneous gene lists [192]. In parallel, context‐specific genetic analyses of CD4+ T‐cell regulation linked OSA risk to genes involved in mitochondrial function, cell migration, and transcriptional control, further supporting the concept that immune‐cell state is part of the disease‐susceptibility architecture rather than a purely downstream epiphenomenon [193]. More granular human molecular data are beginning to align this concept with specific regulatory networks. In peripheral‐blood expression studies, OSA was associated with coordinated correlations among BMAL1/CLOCK/CRY1/PER1, HIF‐related signals, NF‐κB, and BDNF, supporting circadian‐neuroimmune cross‐talk as a plausible susceptibility layer; separately, integrated transcriptomic‐PSG analyses identified NF‐κB‐centered programs with candidate hub genes such as CEBPB and SPI1, suggesting that molecular vulnerability may map onto reproducible signaling modules even when individual biomarkers vary across cohorts [194, 195].

The susceptibility landscape may also include host–microbiome interfaces. Recent evidence synthesis indicates reduced microbial diversity and consistent shifts in gut and oral community structure in OSA. Concurrently, contemporary reviews argue that dysbiosis could interact with IH, sympathetic signaling, and mucosal immune activation to shape systemic inflammatory tone. Although causal direction remains unsettled, these findings broaden the concept of inflammatory susceptibility from cytokine signaling alone to a more distributed host–microbiome network [196, 197].

Beyond broad dysbiosis patterns, recent multiomics work integrating Mendelian randomization, proteomics, methylation, expression‐QTL analysis, and PheWAS did not support a simple direct gut‐microbiome→sleep‐apnea causal pathway; instead, it identified bidirectional relationships with inflammatory proteins, including CCL28 and TIMP4, arguing that host inflammatory signaling may be a more proximal component of susceptibility architecture than taxonomic shifts alone [198].

Primary mechanistic work is also beginning to connect IH to specific immune‐remodeling circuits rather than to generic “inflammation.” In a 2025 chronic‐OSA/hypoxia–reoxygenation study, atrial susceptibility to fibrillation was linked to macrophage M1 polarization driven by an HIF1α–MIF–CD74–NF‐kB axis, providing direct experimental support for the notion that hypoxia‐responsive immune signaling can translate sleep‐disordered breathing into organ‐specific remodeling programs. Such data do not prove that the same pathway operates uniformly across all OSA complications. However, they materially strengthen the mechanistic plausibility of inflammation as part of the susceptibility architecture rather than merely a downstream biomarker [21, 186]. Table 2 provides an integrated overview of the principal mechanistic domains and contextual modifiers that govern OSA expression, linking the underlying pathophysiology to the PSG phenotype and potential therapeutic implications.

**TABLE 2:** Integrated pathophysiological domains, endotypic modifiers, and translational implications in obstructive sleep apnea.

Pathophysiological domain	Core mechanistic summary	How it shapes clinical/PSG expression	Translational or therapeutic implications
Dynamic obstructive event cycle	OSA events are dynamic cycles in which sleep‐related loss of upper‐airway support, progressive flow limitation, rising inspiratory effort, intermittent hypoxemia/hypercapnia, autonomic activation, and ventilatory overshoot recurrently reset the system toward renewed collapse [14, 114].	Event burden is not adequately captured by AHI alone 7; hypoxic, ventilatory, and heart‐rate burden, stage distribution, and event duration better reflect biological stress and downstream vulnerability [7, 8, 118, 119].	Supports phenotyping beyond event counts, with physiological‐burden metrics and event morphology incorporated into risk stratification and treatment selection [117, 118]
Anatomical susceptibility	Structural crowding, craniofacial geometry, soft‐tissue loading, and site‐specific pharyngeal vulnerability determine passive collapsibility [85, 114]; active compensation may only partially offset this mechanical load [142].	Anatomical risk is heterogeneous across palatal, lateral‐wall, tongue‐base, and epiglottic patterns, so that the same AHI can arise from different collapse configurations and response profiles [131, 132, 134].	Explains why mandibular advancement, positional strategies, HNS and surgery behave as phenotype‐dependent rather than purely “structural” therapies [140, 143, 144]
Neuromuscular responsiveness	Whether anatomy translates into frank obstruction depends on upper‐airway dilator recruitment and dynamic airway compensation [60, 112, 148, 149]; over time, vibration‐ or trauma‐related sensory‐motor injury may further blunt reflex protection [126, 149].	This vulnerability is accentuated at sleep onset [14, 114] and can be captured not only functionally but also biomechanically, including altered tongue‐muscle stiffness [154] and abnormal timing/force balance across the respiratory cycle [155].	Provides a rationale for HNS [145], noradrenergic–antimuscarinic pharmacotherapy and combination regimens [146, 147], as well as targeted stimulation and exercise‐based approaches [152, 153], while also cautioning that sedative‐assisted phenotyping can distort the endotype being measured [131, 156]
Ventilatory control instability	High loop gain amplifies the consequences of obstruction by coupling postevent hyperventilation, hypocapnia, and reduced drive to renewed airway vulnerability [112, 157]; a narrow CO_2_ reserve further facilitates this reset toward recurrent collapse [114, 157].	Loop gain varies across sleep stage, circadian timing, background chemosensitivity and comorbidity, and should therefore be decomposed mechanistically rather than treated as a single monolithic trait [159, 162].	Favors phenotype‐matched use of ventilatory‐control stabilizers such as carbonic‐anhydrase inhibitors, oxygen, or combination regimens [146, 158], with response depending on the balance between collapsibility and unstable control [146, 160, 161]
Arousal threshold and sleep stage	A low arousal threshold promotes short, frequent, fragmented events [85, 112], whereas a higher threshold may either allow compensatory recruitment or prolong obstruction and deepen desaturation, depending on the accompanying endotype [114, 118].	Stage‐specific physiology matters: REM‐predominant OSA often reflects worsening collapsibility or withdrawal of ventilatory drive [166, 167], and sex‐related differences in arousability further modify presentation and symptom burden [60].	Supports selective—not indiscriminate—use of hypnotic strategies [164] and argues for integrating REM burden [167, 168], arousal intensity and continuous sleep‐depth metrics [169, 170, 171] when estimating prognosis and treatment benefit
Lung volume and respiratory mechanics	Reduced end‐expiratory lung volume lowers caudal traction on the trachea and adjacent tissues, thereby increasing pharyngeal collapsibility—particularly with obesity, supine posture and sleep‐related mechanical unloading [14, 114, 172, 173].	Mechanical coupling helps explain why respiratory event frequency may dissociate from hypoxic severity and why identical anatomy can behave differently across posture, REM sleep and chronic lung disease contexts [128, 172, 174].	Highlights modifiable leverage points, such as head‐of‐bed elevation and mechanics‐aware positional interventions, and frames lung volume as an amplifier of airway stability rather than a passive background variable [135, 172]
Contextual modifiers	OSA expression can acutely shift with posture and rostral fluid redistribution [14, 135, 175, 177], as well as with nasal obstruction [178] and alcohol/sedative exposure [96], because these modifiers alter collapsibility, neuromuscular compensation, arousability, and loop gain in real time [14, 177].	Environmental conditions such as humidity or temperature and small positional changes can measurably reshape event expression across nights, contributing to short‐horizon variability within the same patient [177, 179, 181, 182].	Reinforces the value of context‐sensitive phenotyping and low‐burden interventions—especially positional and exposure‐related strategies—before assuming a fixed, immutable disease pattern [177, 182]
Genetic, inflammatory, and multiomic susceptibility	Baseline vulnerability appears polygenic and biologically layered, with inflammatory signaling, immune remodeling, proteomic/epigenetic pathways, and microbiome‐associated mechanisms interacting with obesity and cardiometabolic load [183, 184, 186, 187, 196, 197].	These upstream susceptibility programs help explain why OSA burden, comorbidity coupling and downstream tissue injury differ markedly across individuals, even at similar conventional PSG severity [112, 113, 184, 188].	Supports a precision‐medicine framework in which inherited and pathway‐level biomarkers may eventually complement endotyping for risk stratification, patient selection, and mechanistically targeted interventions [21, 183, 186, 191, 192, 198]

Integrated pathophysiological domains, endotypic modifiers, and translational implications in obstructive sleep apnea. The table integrates the major domains that shape OSA expression, including the dynamic obstructive event cycle, anatomical susceptibility, neuromuscular responsiveness, ventilatory control instability, arousal threshold and sleep‐stage effects, lung volume and respiratory mechanics, contextual modifiers, and genetic/inflammatory multiomic susceptibility. For each domain, the table links core mechanisms to their expected polysomnographic and clinical expression and highlights the corresponding translational or therapeutic implications. Collectively, the framework emphasizes that OSA severity and consequences are not determined solely by the apnea–hypopnea index (AHI), but emerge from interacting anatomical, physiological, behavioral, and biological factors that influence disease heterogeneity, prognostic burden, and treatment responsiveness.

*Abbreviations*: AHI, apnea–hypopnea index; CO_2_, carbon dioxide; HNS, hypoglossal nerve stimulation; OSA, obstructive sleep apnea; PSG, polysomnography; REM, rapid eye movement.

### 3.9 Mechanistic Pathways Linking OSA to Fibrosis

#### 3.9.1 Intermittent Hypoxia

IH, a hallmark feature of OSA, is defined by recurring cycles of oxygen deprivation followed by reoxygenation during sleep [199, 200, 201]. Beyond event frequency, the biological “dose” of IH depends on desaturation depth and duration, cycle frequency, and reoxygenation kinetics; accordingly, physiologically informed metrics, such as the sleep apnea‐specific hypoxic burden, have been proposed to better capture exposure heterogeneity and downstream risk [6, 202, 203]. These cyclical fluctuations in oxygen levels underpin OSA pathophysiology and contribute to its systemic complications. IH serves as a potent inducer of OS, systemic inflammation, and sympathetic activation. During hypoxia, the imbalance between ROS production and the body's antioxidant defenses leads to oxidative damage at the cellular and molecular levels. Mechanistically, repeated hypoxia–reoxygenation promotes bursts of ROS (notably during reoxygenation) from mitochondria and oxidant enzymes (e.g., NADPH oxidases [NOX] and xanthine oxidase), leading to endothelial dysfunction and vascular remodeling in experimental models [202, 204, 205]. These oxidative bursts activate inflammatory pathways, leading to the recruitment and activation of immune cells and the release of proinflammatory cytokines. Recent integrative reviews link IH to activation of TLR4/MyD88/NF‐κB/MAPK signaling and NLRP3 inflammasome priming/activation (with interleukin [IL]‐1β and IL‐18 release), providing a mechanistic bridge from oxidant stress to sustained tissue inflammation [16, 187, 206]. Over time, chronic inflammation sustained by IH persistently engages fibrotic pathways, promoting the excessive deposition of ECM components, primarily collagen. This pathological process results in fibrosis, characterized by abnormal thickening and scarring of affected tissues [207, 208]. Fibrosis not only impairs the structural and functional integrity of organs but also contributes to disease progression and complications in OSA.

**FIGURE 2:**
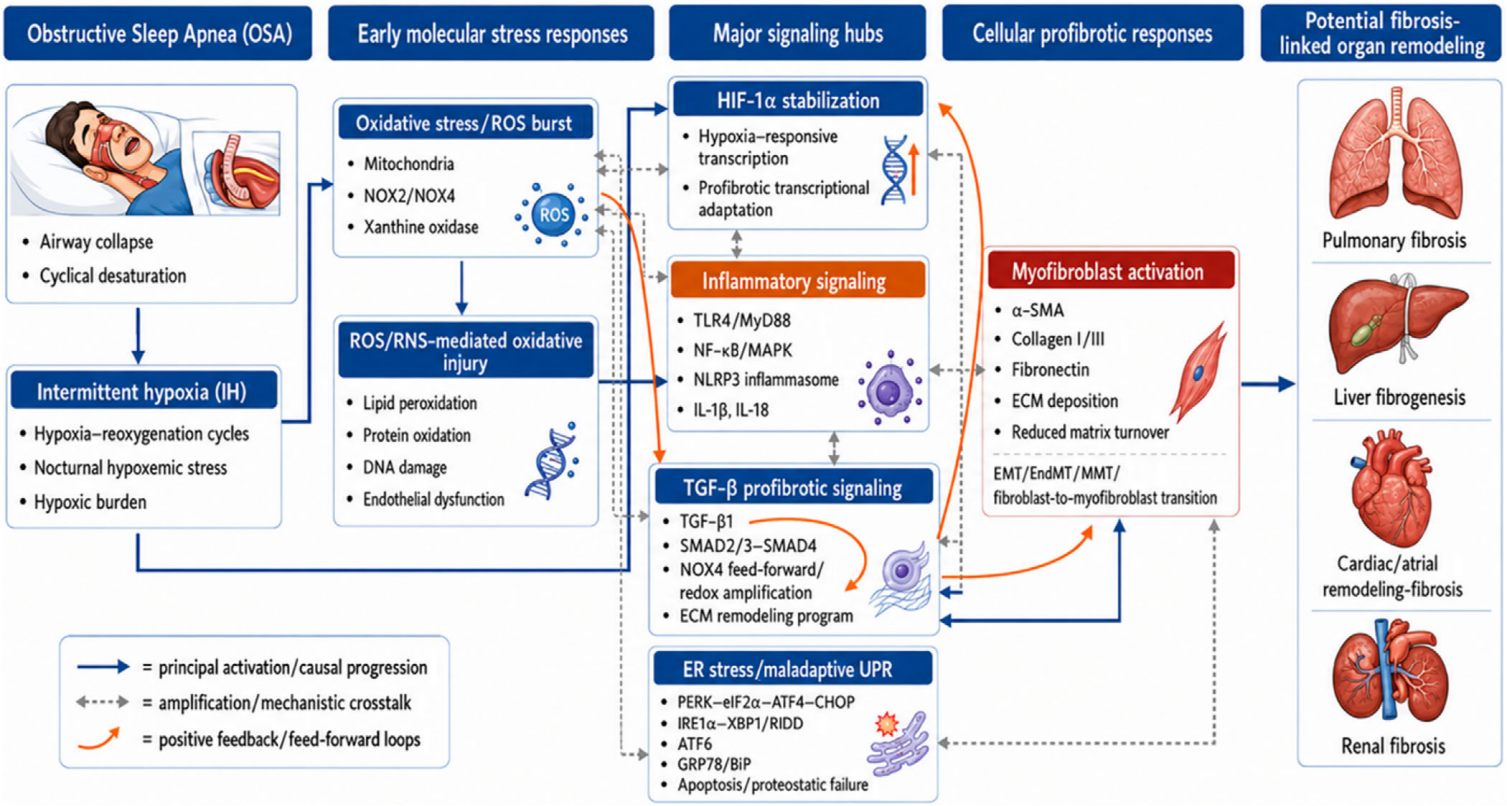
Mechanistic framework linking obstructive sleep apnea to fibrosis‐linked multiorgan remodeling. The figure summarizes molecular pathways through which OSA‐related IH may promote fibrosis‐linked remodeling in susceptible organs. Recurrent upper‐airway collapse causes cyclical desaturation–reoxygenation and nocturnal hypoxemic stress, triggering mitochondrial, NADPH oxidase, and xanthine oxidase‐dependent ROS/RNS production. These redox events drive oxidative injury and endothelial dysfunction and converge with inflammatory and profibrotic signaling. Key hubs include HIF‐1α stabilization, TLR4/MyD88, NF‐κB/MAPK and NLRP3 inflammasome activation, TGF‐β‐centered profibrotic signaling, and maladaptive ER stress/UPR responses. TGF‐β is represented as a central profibrotic convergence node, whereas ER stress amplifies redox imbalance, inflammatory activation, proteostatic failure, apoptosis, and aberrant repair. Together, these mechanisms promote myofibroblast activation, mesenchymal transition programs, ECM deposition, reduced matrix turnover, and progressive tissue stiffening, potentially contributing to pulmonary fibrosis, liver fibrogenesis, cardiac/atrial remodeling and fibrosis, and renal fibrosis. The strength of evidence and causal inference varies across organs. Solid arrows indicate principal activation or pathway progression; dashed arrows indicate amplification or mechanistic crosstalk; curved arrows indicate positive feedback or feed‐forward reinforcement. *Abbreviations*: α‐SMA, alpha‐smooth muscle actin; ATF4, activating transcription factor 4; ATF6, activating transcription factor 6; BiP/GRP78, binding immunoglobulin protein/glucose‐regulated protein 78; CHOP, C/EBP homologous protein; ECM, extracellular matrix; eIF2α, eukaryotic initiation factor 2 alpha; EMT, epithelial‐to‐mesenchymal transition; EndMT, endothelial‐to‐mesenchymal transition; ER, endoplasmic reticulum; HIF‐1α, hypoxia‐inducible factor‐1 alpha; IH, intermittent hypoxia; IRE1α, inositol‐requiring enzyme 1 alpha; MAPK, mitogen‐activated protein kinase; MMT, macrophage‐to‐myofibroblast transition; MyD88, myeloid differentiation primary response 88; NF‐κB, nuclear factor kappa B; NLRP3, NOD‐like receptor family pyrin domain‐containing protein 3; NOX2/NOX4, NADPH oxidase isoforms 2 and 4; OSA, obstructive sleep apnea; PERK, protein kinase R‐like endoplasmic reticulum kinase; RIDD, regulated IRE1‐dependent decay; ROS/RNS, reactive oxygen/nitrogen species; SMAD, small mothers against decapentaplegic; TGF‐β, transforming growth factor beta; TLR4, toll‐like receptor 4; UPR, unfolded protein response; XBP1, X‐box binding protein 1.

The interplay between OS, inflammation, and fibrosis plays a central role in IH‐induced tissue remodeling. IH‐related OS enhances the activation of profibrotic mediators, such as transforming growth factor‐beta (TGF‐β) and hypoxia‐inducible factor 1‐alpha (HIF‐1α), which regulate key processes, such as epithelial‐to‐mesenchymal transition (EMT), in which epithelial cells acquire mesenchymal characteristics, ultimately contributing to increased ECM production, fibrosis, and endothelial dysfunction. IH triggers a rise in HIF‐1α protein levels, which is seen as an adaptive response to OSA [209]. Recent mechanistic evidence demonstrates that IH can directly induce a myofibroblast phenotype and ECM production. In human lung fibroblasts, IH increases α‐SMA and Collagen I expression through activation of HIF‐1α‐TGF‐β/Smad signaling, while pharmacologic or genetic inhibition of HIF‐1α partially reverses these profibrotic responses [18]. Across multiple organs, the cumulative IH (CIH) dose and exposure duration determine whether responses remain adaptive or progress to maladaptive remodeling. Long‐term models indicate cumulative structural injury and accelerated cardiovascular aging, accompanied by increased mortality [202, 206, 210]. Within a multiorgan fibrosis framework, CIH has been associated with myocardial fibrotic remodeling, mediated in part by interorgan signaling from senescent visceral adipose tissue, which underscores systemic cross‐talk beyond local hypoxic stressors [211]. Furthermore, even low‐frequency, mild‐gradient IH can promote liver fibrogenesis in fatty liver models, suggesting that fibrotic risk is not confined to severe IH patterns when metabolic vulnerability exists [212]. Collectively, these mechanisms result in widespread fibrotic changes across various organs, including the lungs, liver, heart, and kidneys [213, 214, 215, 216]. An integrated overview of these interconnected mechanisms is provided in Figure 2.

By driving OS and inflammation, IH may amplify fibrosis‐related remodeling and compound the systemic burden of OSA. These pathways provide potential targets for interventions aimed at reducing hypoxia‐linked tissue injury, although clinical evidence for the reversal of established fibrosis remains limited. At the cellular level, IH differs from sustained hypoxia by generating repeated hypoxia–reoxygenation cycles that favor ROS bursts and redox‐sensitive signaling. HIF‐1α is periodically stabilized during hypoxic nadirs, and in CIH models, this HIF signaling is coupled to inflammatory activation and ECM remodeling programs [216, 217]. Mechanistically, IH can directly promote fibroblast activation and ECM production. In human lung fibroblasts, IH induces Collagen I and α‐SMA expression and activates the HIF‐1α–TGF‐β/Smad axis, while pharmacologic HIF‐1α inhibition partially reverses these profibrotic responses [18]. In parallel, IH can drive endothelial‐to‐mesenchymal transition (EndMT), providing an additional cellular source of myofibroblast‐like cells; for example, IH‐exposed endothelial cells show EndMT features linked to HIF‐1α‐dependent regulatory RNA circuits [218].

Experimental and translational studies further support a causal role of ongoing IH exposure in fibrotic progression. In a bleomycin‐induced pulmonary fibrosis model, interruption of CIH reduces fibrosis severity and macrophage infiltration, consistent with IH as an amplifier of profibrotic lung remodeling [219]. Beyond the lung, long‐term IH exposure in lean mice induces hepatic inflammatory pathways and a steatohepatitis‐like transcriptional signature, with parallel signals detected in a cohort of lean OSA patients [220]. Cardiovascular remodeling shows similar vulnerability: OSA/CIH is associated with atrial inflammation and collagen deposition in combined clinical and mouse data [221], and CIH rat models demonstrate myocardial injury accompanied by myocardial fibrosis that is modifiable through HIF‐1α‐centered pathways [222]. Collectively, these data argue that the “dose” of nocturnal hypoxemia (rather than event counts alone) is a biologically meaningful driver of downstream remodeling. Consistent with this concept, hypoxic burden and related physiological burden metrics capture clinically relevant hypoxemic stress and may better stratify patients at risk for hypoxia‐linked end‐organ injury [7, 8, 203].

A further mechanistic refinement is that IH should not be conceptualized as a uniform HIF‐activating stimulus across all cellular compartments. Instead, the biological response to IH is determined by multiple parameters, including oscillation frequency, desaturation depth, reoxygenation kinetics, concurrent hypercapnia, and the intrinsic oxygen‐sensing architecture of the exposed tissue. These variables determine whether IH preferentially stabilizes HIF‐1α, activates NF‐κB‐dominated inflammatory programs, or rewires proliferative and cell‐cycle regulatory networks. In endothelial cells, sustained hypoxia typically induces a canonical HIF‐1α‐driven transcriptional program, whilst IH preferentially engages inflammatory and cell‐cycle signaling pathways, highlighting that oscillatory hypoxia can remain strongly remodeling‐prone even when a classical sustained‐hypoxia gene signature is incomplete or compartment specific [202, 223]. This distinction is biologically important because fibrosis‐relevant signaling in OSA likely arises not simply from “more hypoxia = more HIF,” but from repeated cycles of redox and mechanotranscriptional stress that recurrently reset endothelial, epithelial, immune, and stromal cell states toward maladaptive repair programs. In this framework, IH becomes fibrogenically relevant not merely because hypoxia is present, but because recurrent desaturation–reoxygenation cycles progressively convert an initially adaptive response into a cumulative injury signal, whose biological impact depends on exposure duration, oscillatory characteristics, and reversibility of the hypoxic stimulus. Consistent with this dynamic model, interruption of chronic CIH has been shown to attenuate ongoing pulmonary fibrosis, supporting the concept that hypoxia‐driven fibrotic remodeling follows a dose‐dependent, potentially reversible trajectory rather than reflecting a purely binary response to hypoxic exposure [18, 219].

#### 3.9.2 OS and Reactive Oxygen Species

OS refers to an imbalance between the production of ROS and reactive nitrogen species (RNS) and the capacity of antioxidant systems to neutralize or repair their effects. In OSA, repetitive hypoxia–reoxygenation during IH acts as a chronic ischemia–reperfusion‐like stimulus, producing recurrent oxidant bursts and a systemic redox imbalance that may persist beyond sleep [224, 225, 226, 227]. Clinical cohorts consistently report higher total oxidant status and OS indices with increasing OSA severity and nocturnal hypoxemia metrics (e.g., T90 and oxygen desaturation measures), together with reductions in antioxidant capacity [224]. Notably, metrics that capture nocturnal hypoxic load (e.g., hypoxic burden) may track oxido‐inflammatory activation and vascular injury pathways better than AHI alone, supporting a dose–response view of IH‐driven ROS injury [205]. Beyond global indices, OSA is associated with increases in lipid peroxidation products (malondialdehyde [MDA]/TBARS, F2‐isoprostanes), oxidative DNA damage (8‐OHdG), and protein oxidation markers such as advanced oxidation protein products (AOPPs), alongside alterations in antioxidant defenses (SOD, catalase, glutathione peroxidase, thioredoxin system) and reduced total antioxidant capacity [201, 225, 226]. A recent systematic review/meta‐analysis reported markedly higher circulating AOPPs in OSAHS versus controls, supporting protein oxidation as a robust readout of chronic OS in this context [228]. In parallel, persistent hyperoxidation of erythrocytic peroxiredoxin‐2 (Prx2‐SO2/3) has been proposed as a feasible blood‐based biomarker of cumulative OS in untreated OSA, remaining elevated into the evening and scaling with AHI severity [227].

Mechanistically, excessive ROS/RNS in OSA arise from multiple interacting sources associated with IH [226]. Recurrent cycles of hypoxia–reoxygenation promote mitochondrial electron transport chain dysfunction with increased leakage of electrons and enhanced ROS generation from Complexes I and III [229, 230]. Parallel activation of membrane‐bound NOX under IH contributes significantly to superoxide production in leukocytes and vascular cells, linking oxidative burst to endothelial and systemic dysfunction [231, 232]. Additional enzymatic pathways, such as xanthine oxidoreductase, also generate ROS during hypoxia/reoxygenation, contributing to vascular OS in both experimental models and clinical OSA contexts [232, 233]. Furthermore, uncoupled endothelial nitric oxide synthase (eNOS) has been implicated as a source of ROS/RNS and as a mediator of impaired nitric oxide (NO) bioavailability and endothelial dysfunction in OSA [231, 232]. Translational IH models provide a direct mechanistic bridge between redox imbalance and remodeling phenotypes, demonstrating increased ROS and MDA, disruption of the BH4/BH2 balance with eNOS uncoupling, and collagen deposition/vascular remodeling [234]. Rodent studies further show that IH increases oxidative and inflammatory markers, leukocyte infiltration, apoptosis, and reduces eNOS expression/activity in arteries, with effects scaling with exposure duration and hypoxic dose [205]. Consistent with a causal role for hypoxia signaling, HIF‐1α silencing attenuates lipid peroxidation, inflammation, and collagen‐rich vascular remodeling in CIH models [235].

At the signaling level, IH activates redox‐sensitive transcriptional programs. Stabilization of HIF‐1α during hypoxic phases and its crosstalk with NF‐κB during reoxygenation promote the expression of inflammatory mediators, adhesion molecules, and pro‐oxidant enzymes, thereby amplifying ROS generation and sustaining immune activation [186, 225]. ROS overproduction drives molecular injury (lipid peroxidation, protein carbonylation/oxidation, and DNA damage) while acting as a signaling input that interfaces with inflammation and fibrosis [225]. In lung fibroblasts, IH can engage HIF‐1α and downstream TGF‐β/Smad pathways, promoting α‐SMA induction and excessive Collagen I/ECM production [20]. In parallel, OSA‐relevant IH exacerbates experimental pulmonary fibrosis and myofibroblast activation while amplifying NOX4 expression and OS, implicating a NOX4–ROS axis as a key profibrotic effector [20]. Human biomarker work supports convergence between OS and fibrotic signaling, with higher OSA severity associating with increased total oxidative status and higher TGF‐β1 mRNA expression in peripheral blood mononuclear cells [236]. ROS/RNS should therefore be viewed not only as damaging byproducts but also as mediators that can prime profibrotic pathways (e.g., TGF‐β/Smad, YAP/TAZ, and NLRP3 inflammasome activation), facilitating myofibroblast differentiation and ECM accumulation [186, 237].

Across organs, CIH models reinforce the concept that OS is a driver—not merely a byproduct—of fibrotic remodeling: (i) in lung “dual‐hit” settings, IH worsens bleomycin‐induced fibrosis and OS within a NOX4‐dependent framework [19]; (ii) in metabolic liver disease, even low‐frequency/mild‐gradient IH increases OS and liver fibrogenesis [212]; (iii) in kidney, CIH induces renal fibrosis with ROS accumulation and lipid peroxidation, with attenuation via activation of antioxidant defenses (Nrf2/GPX4) and suppression of ferroptosis [238]; and (iv) in heart, CIH induces myocardial ROS accumulation, mitochondrial dysfunction, and inflammasome‐linked injury programs that can converge on profibrotic remodeling over time [239]. Therapeutically, intervention data support clinical relevance of this axis: a meta‐analysis of clinical studies found that CPAP therapy increases total antioxidant capacity, with effect estimates influenced by baseline disease severity and patient characteristics [240]. Collectively, these data position OS as a central mechanistic node linking IH to fibro‐inflammatory remodeling in OSA, acting upstream and in feed‐forward loops with HIF/TGF‐β and immune pathways, and providing a mechanistic bridge to the TGF‐β‐centered [186, 225].

From a mechanistic perspective, the profibrotic relevance of ROS in OSA depends not only on the quantity of oxidants but also on their localization and persistence. Mitochondrial ROS, NOX‐derived superoxide, and radicals generated from eNOS uncoupling target partially overlapping yet distinct signaling pathways, thereby differentially influencing HIF stability, TGF‐β activation, inflammasome priming, and matrix‐remodeling enzymes. This spatial compartmentalization of redox signaling may explain why biomarkers of cumulative oxidation, such as hyperoxidized erythrocytic peroxiredoxin‐2, remain abnormal beyond the nocturnal recording window, whereas more labile markers more accurately reflect acute reoxygenation injury [205, 227]. These observations support a transition model in which repeated ROS pulses initially function as second messengers but, following the failure of antioxidant buffering and NO/BH4 coupling, become structurally injurious, promoting endothelial rarefaction, fibroblast activation, and matrix cross‐linking. Therefore, OS in OSA should not be regarded as a uniform “ROS excess” phenomenon. The critical issue is to determine which redox compartments remain persistently dysregulated despite reoxygenation (mitochondria, NOX systems, uncoupled eNOS, or extracellular protein oxidation) as this persistence likely determines whether injury resolves or progresses to stable matrix remodeling [205, 238].

#### 3.9.3 TGF‐β Pathway

TGF‐β is the dominant profibrotic cytokine and a core regulator of tissue remodeling. Its signaling is mediated primarily through the canonical TGFβR2/TGFβR1 (ALK5)–SMAD2/3–SMAD4 axis, negatively controlled by SMAD7, but is also reinforced by noncanonical pathways such as MAPK, PI3K/AKT, and Rho/ROCK. These interconnected signaling branches converge on myofibroblast activation, excessive ECM deposition, and impaired matrix turnover, thereby driving progressive fibrotic remodeling [241].

A major feature of TGF‐β biology is its bidirectional interplay with OS. On the one hand, TGF‐β promotes ROS generation while attenuating antioxidant defenses, thereby shifting the intracellular milieu toward a pro‐oxidant, profibrotic state [242]. On the other hand, TGF‐β is secreted as an inactive LAP/LTBP‐bound latent complex that requires extracellular activation. OS, together with protease‐ and integrin‐dependent mechanisms, can liberate bioactive TGF‐β from this latent reservoir, establishing a self‐amplifying ROS–TGF‐β feed‐forward loop that stabilizes and perpetuates fibrotic signaling [242, 243]. Although the TGF‐β family comprises three isoforms, TGF‐β1 is the primary isoform implicated in fibrotic remodeling in OSA and is widely considered the most informative mechanistic marker of pathway activation in this context. Methodologically, however, interpreting circulating or plasma TGF‐β requires caution, as preanalytical handling may artifactually activate latent pools. By contrast, airway‐proximal matrices, such as exhaled breath condensate, may more accurately reflect local remodeling activity [244]. Consistent with this framework, untreated OSA has been associated with increased levels of soluble and/or active TGF‐β1, as well as downstream indicators of pathway activation, and these signals appear to scale with hypoxic burden. Nevertheless, this relationship is unlikely to be uniform across patients, as obesity‐related factors, including leptin signaling, may substantially shape interindividual variability in TGF‐β‐driven responses [236, 245, 246].

ROS and HIF‐1α have been identified as critical drivers of hypoxia‐induced TGF‐β1 upregulation. In OSA‐relevant IH models, repeated hypoxia–reoxygenation cycles stabilize HIF‐1α and increase TGF‐β/Smad signaling; in human lung fibroblasts, IH induces myofibroblast differentiation and excessive ECM production through activation of the HIF‐1α–TGF‐β/Smad axis, and this profibrotic shift is attenuated by pharmacologic HIF‐1α inhibition [18]. These findings align with landmark hypoxia models showing that mitochondrial ROS and HIF activity are required for hypoxia‐induced endogenous TGF‐β1 signaling and EMT [247]. Recent mechanistic work has further clarified redox‐to‐hypoxia crosstalk: TGF‐β1 can induce NOX2/NOX4‐derived ROS that inactivate PHD2 and hyper‐stabilize HIF‐1α (“oxidative hypoxia”), strengthening the HIF‐1α–TGF‐β feed‐forward loop [242, 243]. Consistent with pathway activation in human disease, SMAD4 is overexpressed in circulating monocytes from OSA patients, and IH can promote SMAD4 upregulation and extracellular release via inflammasome‐linked mechanisms, supporting a TGF‐β pathway signature in hypoxemic phenotypes [248]. Therapeutic reversal of IH exposure can partially normalize TGF‐β‐related signals in airway‐proximal samples and in some circulating biomarker panels, although serum responses are variable across cohorts and sampling matrices [244, 249].

Beyond biomarkers, organ‐level models support causality: (i) IH exacerbates bleomycin‐ and TGF‐β1‐driven pulmonary fibrosis via KDM6B‐dependent, NOX4‐amplified OS; and (ii) chronic IH promotes atrial structural remodeling and fibrosis with increased atrial TGFβ1 and collagen transcripts in experimental models, mirrored by clinical associations in OSA cohorts [19, 221]. The activation of TGF‐β1 pathways in OSA patients leads to widespread cellular and molecular effects, including EMT, myofibroblast differentiation—the transformation of fibroblasts into myofibroblasts, which are key producers of ECM proteins—and upregulation of collagen synthesis. Downstream, canonical SMAD signaling cooperates with noncanonical pathways to induce profibrotic transcriptional programs (e.g., ACTA2/α‐SMA, COL1A1/COL3A1, FN1), promote EMT/EndMT‐like transitions, and suppress ECM degradation, thereby locking in a high‐ECM, high‐stiffness microenvironment [241, 247]. Collectively, recent OSA/IH data position TGF‐β signaling as a mechanistic bridge between IH‐driven redox stress and multiorgan fibrotic remodeling, motivating strategies that target upstream IH (CPAP and weight‐loss interventions) and downstream nodes (e.g., HIF‐1α, NOX4, epigenetic regulators such as KDM6B) as antifibrotic levers [18, 19, 244].

An additional mechanistic layer concerns latent TGF‐β activation in the extracellular compartment. In OSA‐relevant tissues, OS, integrin‐mediated traction, protease activity, and matrix stiffening are likely to cooperate in converting latent TGF‐β into its active form, thereby transforming transient hypoxic injury into self‐sustaining profibrotic signaling. Once activated, TGF‐β not only promotes canonical SMAD2/3 transcriptional outputs but also feeds back on redox metabolism by inducing NOX isoforms and weakening antioxidant defenses, which lowers the threshold for repeated reactivation during subsequent IH cycles [18, 241]. This feed‐forward organization helps explain why treatment of upstream OSA physiology may normalize some signals yet incompletely reverse established remodeling once matrix stiffness, fibroblast memory, and local latent‐TGF‐β activation niches have consolidated. Accordingly, TGF‐β signaling in OSA is unlikely to behave as a simple downstream readout of hypoxemia. Once activated, it may acquire partial autonomy through ROS‐dependent feed‐forward reinforcement, matrix stiffening, and continued latent TGF‐β activation, thereby helping explain why profibrotic phenotypes can persist even when respiratory event frequency alone does not fully capture biological burden [241, 242].

#### 3.9.4 Mechanisms Linking TGF‐β, Fibrosis, and OSA

TGF‐β‐dependent and non‐TGF‐β pathways that culminate in fibrotic remodeling are sustained by self‐perpetuating feedback loops. A central trigger is redox imbalance: ROS can activate and induce TGF‐β1, thereby initiating and reinforcing a profibrogenic program that becomes progressively difficult to resolve [242]. In the context of OSA, TGF‐β1 is increasingly recognized as a mechanistic hub connecting IH to OS, ECM deposition, and downstream profibrotic signaling [19, 202].

A key molecular convergence point between hypoxic stress and TGF‐β signaling is the NOX system, particularly NOX4. TGF‐β1 upregulates NOX4, increasing ROS generation and promoting nuclear accumulation of HIF‐1α, which can facilitate hypoxia‐driven EMT and broader remodeling programs [243]. NOX4 is also required for fibroblast‐to‐myofibroblast differentiation in multiple organs: in cardiac fibroblasts, NOX4 is critical for TGF‐β1‐driven myofibroblast conversion [250], while in kidney fibroblasts, TGF‐β1 induces both NOX2 and NOX4, with NOX4 predominating, highlighting organ‐ and context‐specific utilization of NOX isoforms [251]. In OSA‐relevant “dual‐hit” paradigms, IH can exacerbate bleomycin‐induced pulmonary fibrosis via epigenetic remodeling and NOX4–ROS signaling, strengthening the plausibility of NOX4 as a convergent therapeutic target rather than a passive correlate of tissue injury [19]. Accordingly, crosstalk between IH and TGF‐β1 is best interpreted as a redox‐sensitive profibrotic amplifier in OSA [202].

At the cellular level, recurrent hypoxia–reoxygenation cycles generate ROS bursts that activate maladaptive stress programs. In fibroblast models relevant to OSA, these cycles are accompanied by engagement of the HIF‐1α/TGF‐β/Smad axis, increased α‐SMA and Collagen I expression, and enhanced ECM production—findings consistent with a feed‐forward ROS–HIF‐1α–TGF‐β1 circuit that promotes persistent remodeling [18, 202]. Human biomarker evidence aligns with this framework: increasing OSA severity associates with higher systemic oxidative burden and increased TGF‐β1 mRNA expression, supporting a clinically detectable redox–TGF‐β axis [236]. Collectively, these data support the concept that ROS–HIF‐1α–TGF‐β1 signaling can operate as a driver of fibrosis in OSA and represents a biologically plausible target for mechanism‐based antifibrotic intervention [18, 202, 236]:

Beyond its role in upstream signal amplification, TGF‐β1‐induced ROS contributes directly to ECM synthesis, a hallmark of fibrotic remodeling [252, 253]. In OSA‐relevant settings, IH/reoxygenation is sufficient to intensify inflammatory–oxidative signaling that primes fibroproliferative remodeling, and experimental interruption of IH at the onset of fibrosis attenuates lung collagen deposition and reduces disease severity [202, 219]. Under hypoxic conditions, TGF‐β and hypoxia can act synergistically to bias matrix turnover toward accumulation, for example, by suppressing matrix metalloproteinase‐1 (MMP‐1) while augmenting TIMP‐1 and collagen type I secretion in primary human lung fibroblasts, thereby facilitating excessive collagen deposition and ECM expansion [254]. In terms of clinical phenotype, the severity of hypoxemia in OSA correlates with systemic inflammatory and oxidative signatures across large cohorts, reinforcing the biological plausibility of downstream remodeling pathways, including fibrosis [255]. Canonical TGF‐β/Smad signaling is central to fibrogenesis and supports multiple routes to myofibroblast accumulation [256]. TGF‐β1/Smad3 signaling can promote macrophage‐to‐myofibroblast transition (MMT), thereby contributing to the expanding pool of ECM‐producing cells in chronic tissue injury [256]. Consistently, kidneys from CIH rat models exhibit macrophage infiltration and MMT features that appear to contribute to renal fibrosis [24]. In vascular tissues, chronic OSA models show that increases in Collagen I/III, TGF‐β1, and Smad3 expression are strongly associated with aortic fibrosis [257]. Mechanistically, NOX4 intersects with this pathway at the level of Smad activation: TGF‐β1‐induced phosphorylation/activation of Smad2/3 is critically dependent on NOX4, linking ROS generation to transcriptional profibrotic output and fibroblast‐to‐myofibroblast differentiation [250]. In line with this, IH‐driven myofibroblast differentiation and excessive ECM production in MRC5 cells are mediated by activation of the HIF‐1α–TGF‐β/Smad axis [18]. Human data further suggest that alterations in the Smad pathway are detectable in OSA, as Smad4 overexpression has been reported in OSA patients and is higher in severe than in mild disease [248]. Patients with severe OSA exhibit elevated Smad4 levels compared with those with mild OSA [248]. Functionally, Smad4 acts as a signal integrator, interacting with regulatory networks that include mTOR, HIF‐1α, and circadian genes, implying that Smad4 may couple hypoxia, metabolism, and timekeeping programs to fibrotic signaling in OSA‐related end‐organ injury [248]. Notably, Smad4 overexpression can enhance HIF‐1α activity, providing an additional potential synergy that amplifies hypoxia‐driven profibrotic pathways [248].

Recent evidence extends the redox–TGF‐β framework beyond intracellular signaling to systemic communication via extracellular vesicles (EVs). A systematic review reports that OSA alters EV number and cargo and links EV signaling to OS and inflammation—mechanistic contexts that can converge on profibrotic pathways, including TGF‐β/Smad programs [258]. In parallel, human biomarker studies indicate that OSA severity is associated with increased TGF‐β1‐related readouts (e.g., TGF‐β1 mRNA) alongside OS metrics, supporting systemic activation of redox–TGF‐β biology in clinical cohorts [236]. Multiomics approaches further suggest that EV cargo (proteins, lipids, miRNAs) captures endothelial injury signatures relevant to remodeling and may be modifiable with effective therapy. In a longitudinal cohort sampled before and after 12 months of adherent CPAP therapy (mean ≥6 h/night), plasma exosomes exhibited differential changes in protein/lipid/miRNA cargo that correlated with improved endothelial barrier function and tube formation in vitro [259]. Complementing these findings, a patient‐derived serum‐EV multiomics study identified fatty acid synthase‐enriched EVs that impaired endothelial viability and disrupted metabolic homeostasis, providing a plausible EV‐mediated link to vascular remodeling programs that can precede or accompany fibrotic remodeling [260]. However, not all proposed EV immune‐checkpoint signals generalize: PD‐L1 expression on circulating small EVs did not differ between controls and moderate‐to‐severe OSA and showed no correlation with AHI or hypoxemia, underscoring the need for cautious biomarker translation [261]. Across EV studies, methodological heterogeneity (e.g., isolation platforms, coisolated lipoproteins, and normalization strategies) remains a major limitation. Updated guidance from the ISEV MISEV2023 position statement provides reporting and quality‐control recommendations that are particularly relevant for blood EV analyses and cross‐cohort comparisons in OSA research [262]. In parallel with EV data, systemic readouts of Smad signaling are detectable in humans: soluble SMAD4 is elevated in OSA and associates with atherosclerosis risk, with IH and NLRP3 implicated mechanistically [248]. Finally, exosomal miRNA remodeling has been linked to cardiomyocyte stress–response pathways in uncomplicated severe OSA (exosomal miR‐320b–FOXM1 axis), supporting the broader concept that EV cargo can reflect stage‐dependent adaptive versus maladaptive responses to chronic IH [263]. In this regard, EV studies in OSA should still be considered mechanistically informative but not yet analytically mature. Indeed, preanalytical handling, isolation chemistry, lipoprotein carryover, and normalization remain major sources of bias; without MISEV2023‐level standardization, apparent disease‐specific cargo signatures risk conflating biology with platform effects [258, 262].

Overall, the TGF‐β–fibrosis axis in OSA is better characterized as a systems‐level amplifier rather than a single linear pathway. NOX4‐dependent ROS generation, NLRP3‐associated SMAD4 release, extracellular‐vesicle‐mediated transfer of regulatory cargo, and hypoxia‐sensitive epigenetic modifiers such as KDM6B can all reinforce a shared endpoint: stabilization of a matrix‐producing myofibroblast state that becomes progressively less dependent on the initiating hypoxic insult [19, 248, 260]. A significant translational implication is that antifibrotic interventions in OSA may require a staged approach, involving early disruption of upstream hypoxia and inflammation, followed by downstream interruption of redox–TGF‐β–epigenetic memory circuits in patients with established organ remodeling. Recent evidence further suggests that this systems‐level amplifier encompasses epigenetic and noncoding RNA layers that can stabilize a profibrotic state. In dual‐hit scenarios, IH enhances fibrosis through KDM6B–NOX4 coupling, while endothelial circular RNAs can promote EndMT and facilitate the conversion of vascular stress into mesenchymal transition and matrix deposition [19, 218].

#### 3.9.5 Inflammation

Chronic inflammation is increasingly recognized as a central pathophysiological feature of OSA morbidity [186, 264]. In this context, IH, the defining physiological disturbance of OSA, acts as a major upstream driver of inflammatory activation. Recurrent cycles of hypoxia and reoxygenation generate OS and redox imbalance, which in turn activate multiple inflammatory signaling pathways, promote the release of proinflammatory cytokines, and stimulate immune cell recruitment and activation. Through these interconnected mechanisms, IH sustains a persistent state of low‐grade systemic inflammation that contributes to the multiorgan morbidity associated with OSA [186, 264]. Recent immunology‐focused syntheses further highlight that the inflammatory consequences of IH are amplified by sleep fragmentation, with both stressors converging on key regulatory hubs, including HIF‐1α, NF‐κB, and inflammasome‐associated pathways. This integrated signaling landscape helps explain how OSA‐related inflammatory dysregulation extends beyond the respiratory system to influence cardiovascular, metabolic, and immune homeostasis [186].

A central upstream driver of IH‐associated inflammation is OS. The repetitive hypoxia–reoxygenation cycles characteristic of IH disrupt the balance between ROS generation and antioxidant defenses, promoting ROS overproduction and oxidative damage to DNA, lipids, and proteins [206, 255]. Beyond direct macromolecular injury, ROS amplify inflammatory signaling via redox‐sensitive transcriptional programs, most prominently NF‐κB, thereby reinforcing cytokine and chemokine production and perpetuating inflammation [186]. Clinical biomarker studies support a tight coupling between hypoxic burden and systemic inflammatory/oxidative signatures. In OSA cohorts, hematologic inflammatory indices and laboratory markers remain independently associated with nocturnal oxygen saturation metrics, supporting the concept that hypoxic burden measures can complement AHI in capturing systemic inflammatory load [255]. Mechanistically, OSA‐like intermittent desaturation–resaturation promotes repeated ROS bursts arising from mitochondrial sources and enzymatic systems such as NOX, and experimental blockade or deletion of NOX2 can blunt CIH‐associated redox‐driven physiological dysfunction in vivo [265]. These redox oscillations engage hypoxia‐ and redox‐sensitive pathways, including HIF‐1α, NF‐κB, and inflammasome signaling, leading to upregulation of adhesion and chemokine programs that promote recruitment and activation of innate immune cells, notably macrophages and neutrophils [186]. In vivo, long‐term IH increases neutrophil/monocyte infiltration and inflammatory pathway activation; CIH also increases macrophage accumulation and can skew macrophage polarization toward a proinflammatory (M1‐like) phenotype in end‐organ disease models [220, 266]. Consistently, human OSA cohorts display circulating signatures of neutrophil activation/degranulation, and neutrophil‐derived biomarkers (e.g., MPO, H_2_O_2_) correlate with oxygen desaturation metrics [267].

Over time, persistent inflammatory activation becomes tightly coupled to tissue remodeling and fibrosis. CIH‐associated chemokine programs and inflammatory infiltrates intersect with profibrotic signaling networks, promoting excessive ECM deposition (e.g., collagen) and progressive organ stiffening and dysfunction [18, 219, 266, 268]. In vitro models incorporating physiologic shear stress further support a direct vascular inflammatory effect of OSA‐like IH: an endothelial IH underflow platform revealed pronounced activation of inflammatory pathways and endothelial activation programs, providing a mechanistic bridge between IH and vascular dysfunction [269]. At the effector stage, IH can also directly promote myofibroblast differentiation and ECM production. In human lung fibroblasts (MRC5), IH increased α‐SMA and Collagen I and activated the HIF‐1α–TGF‐β–Smad2/3 axis; pharmacologic inhibition or HIF‐1α siRNA attenuated these profibrotic responses [18]. In parallel, experimental work indicates that interrupting IH exposure can attenuate fibrotic severity in vivo, with reduced collagen deposition and inflammatory cell accumulation in lung fibrosis models when CIH is stopped [219].

NF‐κB is widely viewed as a central transcriptional hub that integrates IH‐driven redox signals with inflammatory gene programs (cytokines, chemokines, and adhesion molecules), thereby linking OSA to vascular and cardiometabolic complications [186, 206]. Landmark evidence from pediatric human tissue supports local NF‐κB activation and a proinflammatory tonsillar milieu in OSA 269, reinforcing the notion that systemic signatures can reflect tissue‐level inflammatory activation. Emerging clinical immunophenotyping further suggests that treatment‐related immune shifts may track more closely with hypoxic burden (e.g., ODI) than with AHI alone. In a prospective cohort, 3 months of CPAP reduced LPS‐induced monocyte cytokine responsiveness, shifting monocytes toward an anti‐inflammatory CD163^+^CD206^+^ phenotype in proportion to baseline ODI, highlighting oxygen desaturation burden as an actionable driver of innate immune dysregulation [270]. In line with these observations, a meta‐analysis of randomized controlled trials indicates that CPAP—particularly when maintained for ≥3 months—can reduce circulating CRP and IL‐6, with tumor necrosis factor (TNF)‐α reductions more apparent with longer treatment duration and in severe OSA, although heterogeneity across trials remains substantial [271]. Collectively, inflammation and profibrotic remodeling in OSA are increasingly interpreted through a “hypoxic burden → immunometabolic stress → tissue remodeling” framework, in which IH‐driven oxidative and inflammatory signaling provide both priming and amplification for organ‐specific fibrotic programs [186, 206]. Beyond the lung, long‐term IH alone is sufficient to activate the hepatic inflammatory pathway and to induce molecular signatures overlapping with those of human steatohepatitis, supporting a causal contribution of IH to NAFLD/NASH‐related inflammation and downstream remodeling [220]. Experimental work also indicates that normalization of oxygenation can reprogram hepatic metabolism after IH exposure, providing a mechanistic rationale for targeting hypoxic burden in metabolic liver disease contexts [272]. At the organismal level, prolonged IH exposure in murine OSA models accelerates cardiovascular aging phenotypes, increasing mortality, in line with the concept that chronic IH drives a systemic proinflammatory/oxidative milieu that progressively undermines organ resilience [210].

A critical interpretation of the inflammatory literature is that IH‐associated inflammation is unlikely to be merely a parallel epiphenomenon of hypoxemia and may contribute to profibrotic cell‐state transitions. Macrophage polarization, neutrophil‐derived oxidants, monocyte reprogramming, and inflammasome activity shape the cytokine composition of the injured microenvironment, thereby influencing whether tissue repair resolves or progresses to chronic scar‐forming remodeling. The observation that CPAP‐related immune normalization tracks more closely with oxygen desaturation burden than with AHI reinforces the concept that the inflammatory dose relevant to fibrosis is encoded in the depth and temporal structure of hypoxemia rather than in event counts alone [270, 271]. These findings strengthen the rationale for integrating hypoxic‐burden metrics into mechanistically oriented OSA phenotyping, particularly in patients with fibro‐inflammatory comorbidities. They support inflammation as a potentially modifiable contributor rather than a passive correlate, but do not yet establish direct clinical causality for fibrosis in humans [186, 270].

#### 3.9.6 Endoplasmic Reticulum Stress

OSA‐associated IH destabilizes proteostasis through repetitive hypoxia–reoxygenation bursts of ROS, altered endoplasmic reticulum (ER)–mitochondrial Ca^2+^ handling, and transient nutrient/ATP limitation, thereby activating the unfolded protein response (UPR) via PERK, IRE1α, and ATF6 [186]. In a murine IH model (5–21% O_2_ oscillations every 90 s; 8 h/day for 4 weeks), lung Grp78/BiP and CHOP were upregulated, PERK–eIF2α–ATF4 signaling was selectively activated, and ER‐stress‐linked apoptosis (Caspase‐12, cleaved Caspase‐3, TUNEL) coincided with collagen accumulation and increased TGF‐β1 and thrombospondin‐1 (TSP‐1); administration of the chemical chaperone TUDCA attenuated UPR activation and reduced both apoptosis and fibrotic remodeling [273]. Complementing this pulmonary evidence, cross‐organ CIH models support a conserved PERK–ATF4–CHOP inflammatory module: in mice exposed to CIH for 4–12 weeks, white adipose tissue displayed increased SPHK2 expression and PERK–ATF4–CHOP activation with downstream OS, NLRP3 inflammasome accumulation, adipocyte apoptosis and dyslipidemia; SPHK2 inhibition or pharmacologic ER‐stress attenuation (4‐phenylbutyric acid) mitigated these effects [274].

ER stress/UPR signaling can amplify the classical profibrotic pathways activated by IH. In human lung fibroblasts (MRC5), IH increased Collagen I and α‐SMA and activated a HIF‐1α–TGF‐β–Smad2/3 axis, partially reversed by pharmacologic (2‐methoxyestradiol) or genetic (siHIF‐1α) HIF‐1α inhibition [18]. At the epithelial level, maladaptive UPR outputs—particularly IRE1α RNase‐mediated regulated IRE1‐dependent decay (RIDD)—can couple cellular stress to profibrotic epithelial plasticity: selective RIDD inhibition with the kinase modulator PAIR2 preserved adaptive XBP1 splicing, reduced AT2‐to‐damage‐associated transitional cell conversion, and protected mice from bleomycin‐induced pulmonary fibrosis while identifying Fgfr2 mRNA as a direct RIDD substrate [275]. These mechanisms support ER stress as a plausible convergence point for multiorgan fibrotic risk in OSA. In two murine NAFLD models, CIH significantly increased hepatic inflammation and fibrosis and shifted liver macrophages toward an M1 phenotype via upregulation of SPP1; macrophage SPP1 knockdown attenuated CIH‐driven inflammatory and fibrotic injury in hepatocytes [266].

Similarly, CIH (8 h/day for 4 weeks) induced renal injury with increased α‐SMA, Collagen I, and TGF‐β1, and pharmacologic intervention reduced fibrotic remodeling [238]. Consistent with the concept that timely OSA treatment may be disease‐modifying in fibrotic comorbidity, interruption of CIH exposure at the onset of bleomycin‐induced lung fibrosis reduced collagen deposition, fibrosis score, and macrophage density in fibrotic regions in male mice [219]. These interconnected mechanisms are integrated in Figure 2, which links IH‐driven oxidative and inflammatory stress to UPR activation, TGF‐β signaling, and downstream profibrotic remodeling [273, 274, 275].

ER stress may also function as a rheostat that determines whether cells exposed to IH remain adaptive or transition into apoptosis, senescence, or fibrosis‐prone states. Sustained PERK–ATF4–CHOP signaling suppresses proteostatic recovery, promotes mitochondrial dysfunction, and contributes to lipid peroxidation. In contrast, maladaptive IRE1α RNase signaling reshapes epithelial cell identity and alters secretory behavior, thereby activating fibroblasts and macrophages [274, 275]. Within a mechanistic framework, ER stress should therefore be viewed not as an isolated downstream lesion but as an integrative node that couples oscillatory hypoxia, redox damage, inflammatory signaling, and aberrant wound‐healing trajectories across organs. Conceptually, ER stress may serve as the critical checkpoint at which recurrent IH transitions from an adaptive oscillatory stimulus to a maladaptive remodeling program. In this context, current evidence implicates the PERK–ATF4–CHOP signaling branch and indicates that proteostatic failure sensitive to chemical chaperones may regulate the transition toward apoptosis, TSP‐1/TGF‐β1 activation, and collagen accumulation [273].

## 4 Complications

The clinical consequences of OSA can be categorized into two complementary evidence domains. The first domain encompasses established systemic complications, such as hypertension, cardiovascular disease, arrhythmias, stroke, and neurocognitive dysfunction, which are supported by robust epidemiological, mechanistic, and interventional evidence. The second domain involves organ‐specific structural remodeling, in which OSA‐related intermittent hypoxemia, sleep fragmentation, autonomic stress, and inflammatory‐redox signaling may exacerbate fibrotic or remodeling‐prone conditions. This distinction is critical because the strength of evidence varies across outcomes: while cardiovascular and cerebrovascular complications are substantiated by extensive clinical literature, fibrosis‐associated remodeling remains more heterogeneous and is primarily supported by mechanistic, associative, and organ‐specific studies. Established complication pathways should therefore be distinguished from emerging remodeling hypotheses, with physiological burden metrics, particularly nocturnal hypoxemia, interpreted as recurrent determinants of downstream risk

### 4.1 Established Systemic Complications

OSA is now widely recognized as a multisystem disorder in which repetitive upper‐airway obstruction drives intermittent hypoxemia, sleep fragmentation, and large negative intrathoracic pressure swings. These exposures engage convergent downstream programs—sympathetic and chemoreflex activation, renin–angiotensin–aldosterone system signaling, OS and inflammation, endothelial dysfunction, metabolic dysregulation, and prothrombotic hemostatic changes—that jointly promote target‐organ injury across the cardiovascular and cerebrovascular axes [5, 13].

OSA exhibits significant biological and clinical heterogeneity. Traditional severity classification based on the AHI does not fully capture the dose, duration, or hemodynamic consequences of nocturnal respiratory events. Event‐related physiological metrics, such as sleep apnea‐specific hypoxic burden and the acute heart‐rate response to events, frequently demonstrate stronger associations with incident cardiovascular outcomes than AHI alone and may assist in identifying subgroups most likely to benefit from OSA‐directed therapy [8, 10].

This section examines five systemic complications of OSA with the strongest evidence base: systemic hypertension and vascular remodeling, cardiovascular disease and all‐cause mortality, arrhythmias (with emphasis on atrial fibrillation [AF]), stroke and broader cerebrovascular injury, and neurocognitive impairment with dementia risk. The evidence pattern across these outcomes remains consistent. Observational and mechanistic studies support a causal relationship, whereas randomized trials of CPAP in secondary prevention cohorts frequently yield neutral results. These neutral outcomes are primarily due to modest adherence, infrequent endpoints, and the inclusion of participants with a lower physiological burden of OSA. Consequently, there has been a shift toward phenotype‐informed cardiovascular risk stratification in OSA [9, 10].

**FIGURE 3:**
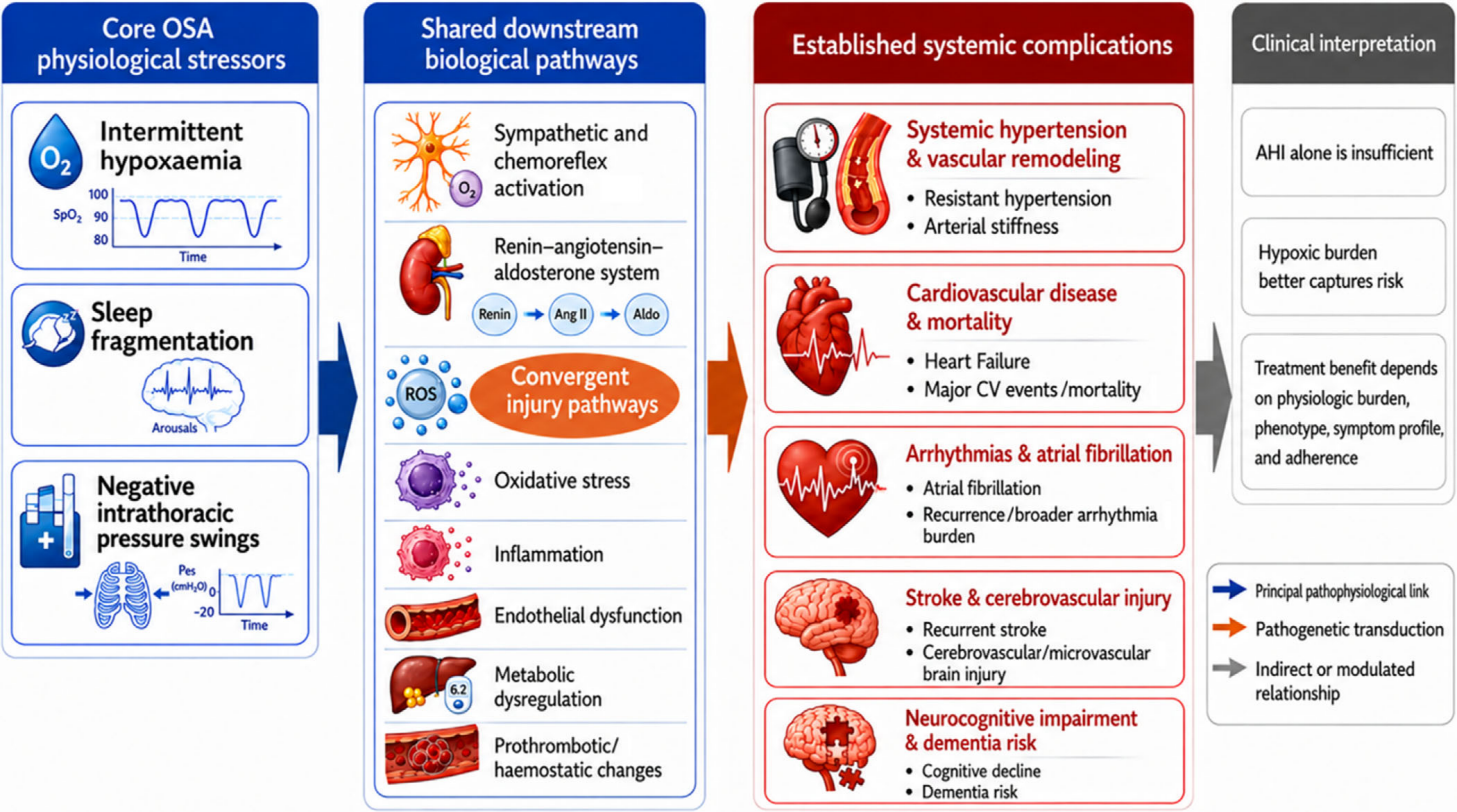
Shared pathophysiological pathways linking obstructive sleep apnea to established systemic complications. This figure summarizes the integrated pathophysiological cascade through which OSA contributes to established systemic complications. Core OSA‐related physiological stressors, including intermittent hypoxemia, sleep fragmentation, and negative intrathoracic pressure swings, activate shared downstream biological pathways, including sympathetic and chemoreflex activation, renin–angiotensin–aldosterone system activation, oxidative stress, inflammation, endothelial dysfunction, metabolic dysregulation, and prothrombotic/hemostatic changes. These convergent injury pathways contribute to five major complication domains with the strongest evidence base: systemic hypertension and vascular remodeling, cardiovascular disease and mortality, arrhythmias with particular emphasis on atrial fibrillation, stroke and broader cerebrovascular injury, and neurocognitive impairment with increased dementia risk. The right‐hand panel highlights the clinical implication that AHI alone incompletely captures risk, whereas hypoxic burden, broader physiological burden, phenotype‐informed interpretation, symptom profile, and treatment adherence may better identify patients at higher risk of complications and those more likely to benefit from treatment. Blue arrows indicate principal pathophysiological links; orange arrows indicate pathogenic transduction from shared biological pathways to downstream complications; gray arrows indicate indirect or modulated clinical relationships. *Abbreviations*: AHI, apnea–hypopnea index; Aldo, aldosterone; Ang II, angiotensin II; cmH_2_O, centimeters of water; CV, cardiovascular; O_2_, oxygen; OSA, obstructive sleep apnea; Pes, esophageal pressure; ROS, reactive oxygen species; SpO_2_, peripheral oxygen saturation.

Recent evidence further supports moving from a single “OSA severity” construct to a multidimensional complication‐risk model combining physiologic burden and symptom expression. In ERJ Open Research, symptom subtypes and hypoxic burden independently predicted distinct cardiovascular outcomes, indicating that symptoms do not fully proxy for physiological injury exposure and supporting trial enrichment strategies that incorporate both dimensions [11]. This shift aligns with emerging endotype‐informed frameworks, including upper‐airway collapsibility, ventilatory control instability, arousal threshold, and muscle responsiveness, that may help tailor interventions and anticipate organ‐specific complications [5]. These shared pathophysiological links and their clinical interpretation are summarized in Figure 3.

#### 4.1.1 Systemic Hypertension and Vascular Remodeling

OSA is strongly linked to prevalent and incident hypertension, with particularly high enrichment in nocturnal, masked, and treatment‐resistant phenotypes. Intermittent hypoxemia and arousal‐related autonomic instability acutely amplify beat‐to‐beat and night‐to‐night BP oscillations and may impair normal nocturnal “dipping,” creating a BP‐variability phenotype that is itself prognostically adverse [276]. These patterns provide a mechanistic bridge between nightly respiratory events and the long‐term development of sustained daytime hypertension.

Mechanistically, repetitive hypoxia–reoxygenation cycles increase tonic and reflex sympathetic activity and augment peripheral vasoconstrictor tone; in parallel, OSA promotes sodium and fluid retention through renin–angiotensin–aldosterone system activation and nocturnal rostral fluid shift, which can simultaneously worsen airway collapsibility and elevate BP [277]. The net result is a self‐reinforcing loop in which upper‐airway obstruction and BP dysregulation mutually potentiate each other, especially in obesity and resistant hypertension.

At the vascular level, OSA‐associated hemodynamic surges and oxidative–inflammatory signaling contribute to endothelial dysfunction and early vascular remodeling, including reduced flow‐mediated dilation (FMD), increased arterial stiffness, and accelerated vascular aging [5, 6]. These intermediate phenotypes help explain why hypertension risk in OSA can appear disproportionate to AHI‐defined severity in some individuals, and why indices that better reflect hypoxemic “dose” may show stronger associations with vascular injury.

Genetic approaches provide an additional layer of inference but also highlight confounding by shared risk factors. A recent bidirectional Mendelian randomization analysis suggested that the apparent causal signal from OSA to hypertension is attenuated after adjustment for BMI and related behavioral covariates, supporting the concept that obesity is both a major upstream driver and a key modifier of OSA‐related hypertension risk [278].

Therapeutically, CPAP typically lowers BP modestly at the population level, but the magnitude of benefit is greater for nocturnal BP and for patients with higher baseline BP and higher nightly treatment use. A large 2025 meta‐analysis of randomized trials reported the strongest effect on nocturnal systolic BP and a dose–response relationship with CPAP use ≥5 h/night [279]. These findings support prioritizing ambulatory BP monitoring and adherence optimization when the clinical goal is vascular risk reduction.

In patients with resistant hypertension, the BP response to OSA treatment can be clinically meaningful. The HIPARCO randomized trial demonstrated that CPAP improved 24‐h and nocturnal BP in patients with resistant hypertension (Martínez‐García et al., 2013). Complementary pharmacologic strategies that target aldosterone excess (e.g., mineralocorticoid receptor [MR] antagonists) may improve both BP control and OSA severity in selected patients, consistent with the RAAS/fluid‐shift model [277].

Finally, evidence is emerging that non‐CPAP therapies may achieve BP effects comparable to those of CPAP in selected populations. In the CRESCENT noninferiority trial, mandibular advancement therapy was noninferior to CPAP for reducing 24‐h mean arterial pressure in hypertensive patients with moderate‐to‐severe OSA, with particularly pronounced reductions in asleep BP [280]. Conversely, a sham‐controlled randomized crossover trial of HNS found no significant change in 24‐h systolic BP with HNS versus sham, underscoring the need for mechanism‐aligned patient selection and rigorous cardiovascular endpoint assessment in device trials [281].

Recent clinical characterization of OSA‐related hypertension has identified four key features: resistant hypertension, nocturnal hypertension, abnormal BP variability, and early vascular remodeling. These findings support the routine use of ambulatory BP monitoring and the explicit targeting of nocturnal BP in suspected OSA‐driven phenotypes [282, 283]. Mechanistically, IH can promote a feed‐forward loop through carotid body hypersensitivity and sustained sympathetic activation, integrating OS and HIF signaling with epigenetic remodeling in chemoreflex pathways [284]. These mechanistic insights refine the interpretation of CPAP BP trials: in a worldwide individual‐patient data meta‐analysis (≈9400 patients), BP reduction was primarily observed in patients with uncontrolled baseline systolic BP, while those with controlled BP experienced minimal additional benefit [285]. Furthermore, a comprehensive 2025 meta‐analysis demonstrated consistent improvement in endothelial function, as measured by FMD, and modest favorable changes in lipid and glucose profiles with CPAP, indicating biologically meaningful modification of intermediate vascular‐risk pathways [286].

#### 4.1.2 Cardiovascular Diseases and All‐Cause Mortality

Beyond hypertension, OSA is associated with a higher risk of incident coronary heart disease, heart failure, and composite major adverse cardiovascular and cerebrovascular events, as well as increased all‐cause mortality in longitudinal cohorts [5, 8]. However, the strength of association varies across age, sex, symptom profile, and comorbidity burden, reinforcing the concept that OSA is a heterogeneous exposure whose cardiovascular impact depends on which physiological burdens predominate.

In this context, physiologic severity metrics have particular value. Hypoxic burden, which integrates the depth and duration of event‐related desaturations, has consistently outperformed AHI in predicting cardiovascular outcomes in cohorts and is increasingly used for risk stratification in both observational and randomized‐trial reanalyses [8, 287]. Mechanistically, greater hypoxic burden plausibly captures a higher dose of OS and hypoxia‐inducible signaling, with downstream effects on endothelial dysfunction, vascular inflammation, and myocardial remodeling [5, 6].

Randomized secondary‐prevention trials of CPAP in established cardiovascular disease have often been neutral when analyzed in aggregate. The SAVE trial, for example, did not show a reduction in major cardiovascular events in intention‐to‐treat analyses, even with modest average CPAP use [9]. These “negative” results have motivated a shift from one‐size‐fits‐all trial designs to enrichment strategies that target high‐risk OSA phenotypes and maximize effective treatment exposure.

Recent analyses support this precision approach. A 2025 multitrial analysis reported that CPAP benefit on major adverse cardiovascular and cerebrovascular events was concentrated in patients with high‐risk OSA profiles defined by greater OSA‐related hypoxemia or heart‐rate acceleration, whereas low‐risk profiles showed little evidence of benefit [10]. Similarly, in a secondary analysis of the RICCADSA cohort, high hypoxic burden, but not an AHI threshold, identified individuals at greater MACCE risk, particularly among untreated or nonadherent participants [287].

Real‐world data also suggest clinically meaningful associations with outcomes when adherence is sustained. In a national Medicare cohort, PAP utilization was associated with lower all‐cause mortality and lower incidence of major adverse cardiovascular events, aligning with the hypothesis that treatment “dose” and long‐term persistence are key determinants of cardiovascular benefit [288]. A large 2025 systematic review and meta‐analysis that integrated randomized and confounder‐adjusted nonrandomized evidence similarly supported a potentially beneficial effect of PAP on all‐cause and cardiovascular mortality [289].

Finally, the therapeutic landscape is expanding beyond airway‐based interventions. In adults with obesity and moderate‐to‐severe OSA, tirzepatide produced large reductions in AHI and hypoxic burden alongside improvements in systolic BP and inflammatory markers, suggesting that disease‐modifying weight‐loss pharmacotherapy could indirectly attenuate downstream cardiovascular risk in appropriate patients [84]. These results reinforce the need to integrate OSA treatment within comprehensive cardiometabolic risk management rather than treating airway obstruction in isolation.

Across cardiovascular endpoints, accumulating data support that the magnitude of nocturnal hypoxic exposure, rather than AHI alone, helps identify those at greatest risk. In the RICCADSA cohort, high hypoxic burden predicted MACCEs, whereas an AHI ≥30/h did not [287]. Consistent with this, symptom subtype and hypoxic burden contributed independently to distinct cardiovascular outcomes, supporting dual‐axis phenotyping (symptoms + physiologic burden) for risk stratification and trial enrichment [11]. Real‐world adherence evidence further indicates that sustained PAP exposure is a key modifiable determinant of benefit [288, 289].

#### 4.1.3 Arrhythmias and AF

OSA is highly prevalent among patients with cardiac arrhythmias, and evidence supports a bidirectional relationship in which OSA promotes arrhythmogenesis. At the same time, rhythm disorders (and their therapies) can worsen sleep and ventilatory control. Contemporary AF guidelines explicitly emphasize evaluation for sleep‐disordered breathing as part of comprehensive risk‐factor modification [290, 291].

Several OSA‐specific mechanisms converge on an AF‐promoting substrate. Repetitive negative intrathoracic pressure swings increase transmural atrial pressure and stretch, promoting electrical and structural remodeling; intermittent hypoxemia and reoxygenation amplify OS and inflammation; and sympathetic surges during arousals increase atrial ectopy and trigger activity. Over time, these processes favor atrial fibrosis and conduction heterogeneity, increasing AF susceptibility and persistence [5, 13].

Clinical data consistently link OSA to incident AF and to AF recurrence after rhythm‐control interventions. In patients undergoing pulmonary vein isolation, a 2025 meta‐analysis reported a higher recurrence risk in OSA patients and suggested that CPAP use may mitigate recurrence, although heterogeneity and residual confounding remain important limitations [292].

The broader spectrum of arrhythmias is also relevant. OSA has been associated with nocturnal bradyarrhythmias, ventricular ectopy, and a potential increase in sudden cardiac death (SCD) risk; however, the evidence is heterogeneous and may depend strongly on treatment status and comorbidity context. A 2025 systematic review and meta‐analysis found insufficient evidence to establish a definitive overall association between OSA and SCD, while subgroup analyses suggested elevated risk in untreated OSA [293].

From a therapeutic standpoint, CPAP plausibly reduces arrhythmia burden by suppressing nocturnal hypoxemia and autonomic surges, but definitive randomized evidence for hard arrhythmia endpoints is limited. The current state of evidence therefore supports a pragmatic, risk‐based strategy: screen for and treat OSA in AF patients—particularly those with obesity, hypertension, or recurrent AF—while recognizing that treatment adherence and underlying OSA phenotype will likely determine the magnitude of rhythm benefit [291, 292].

Mechanistic randomized evidence strengthens causal inference in the OSA–AF link. In the SLEEP‐AF randomized trial, 6 months of CPAP in AF patients with at least moderate OSA improved invasive electrophysiologic substrate measures, consistent with partial reversal of arrhythmogenic atrial remodeling [294]. Complementing mechanistic findings, a large contemporary ablation cohort showed that long‐term CPAP adherence (>1 year, telemonitoring‐confirmed) was associated with reduced very late AF recurrence after catheter ablation in severe OSA [295].

#### 4.1.4 Stroke

OSA is common after stroke and transient ischemic attack (TIA) and is associated with poorer functional recovery and higher risk of recurrent vascular events in observational studies, consistent with shared mechanisms of endothelial dysfunction, impaired cerebral autoregulation, BP variability, and hypercoagulability [13, 276]. Beyond overt stroke, accumulating data suggest that OSA may contribute to subclinical cerebrovascular injury that lies on the pathway to cognitive decline.

Randomized evidence for poststroke PAP therapy has historically been constrained by feasibility and adherence barriers, but synthesis of available trials is increasingly supportive. A 2023 systematic review, meta‐analysis, and meta‐regression of randomized trials concluded that PAP therapy in poststroke sleep‐disordered breathing reduced recurrent vascular events and improved neurological deficit, cognition, and functional independence. However, optimal timing and minimum effective therapeutic dose remain to be defined [296].

Recent population‐based neuroimaging data further expand the concept of OSA‐related cerebrovascular injury. In a large cohort study with longitudinal follow‐up, moderate‐to‐severe OSA was associated with increased risk of incident cerebral microbleeds, an imaging marker linked to small‐vessel fragility and future hemorrhagic and ischemic events, supporting the hypothesis that OSA contributes to microvascular brain pathology beyond classical large‐vessel atherosclerotic pathways [297].

At the same time, null findings in some imaging cohorts remind us that cerebrovascular consequences may be context dependent. For example, a community‐based cohort of cognitively unimpaired older adults reported no association between OSA and progression of white matter hyperintensity volume over 3 years, suggesting that age, baseline vascular risk, and OSA phenotype may modify cerebrovascular vulnerability [298].

Clinically, these data justify systematic attention to sleep‐disordered breathing in cerebrovascular care pathways, particularly for patients with difficult‐to‐control BP, AF, or recurrent events. Future trials that combine early diagnosis, adherence support, and enrichment for high physiological burden are needed to determine whether targeted OSA treatment reduces recurrent stroke and slows progression of covert cerebrovascular disease [10, 296].

Longer‐term observational data help reconcile the mixed results from poststroke CPAP trials. Analysis of a nationwide Danish registry cohort of poststroke or TIA patients with sleep‐disordered breathing revealed that CPAP therapy was associated with improved long‐term survival for up to 14 years; however, no reduction in recurrent stroke or TIA was observed over 5 years [299]. These findings suggest that CPAP may reduce competing cardiovascular mortality but does not fully mitigate recurrence attributable to established cerebrovascular pathology, delayed initiation, or insufficient treatment dose. Future pragmatic trials should explicitly address these issues [296].

#### 4.1.5 Neurocognitive Impairment, Daytime Dysfunction, and Dementia Risk

Neurocognitive impairment is a frequent and clinically meaningful consequence of OSA, encompassing EDS, reduced vigilance and executive function, and impaired memory consolidation. Mechanistically, intermittent hypoxemia and sleep fragmentation can induce synaptic dysfunction and neuroinflammation, while OSA‐related cardiovascular and cerebrovascular injury provides an additional “heart–brain” pathway linking OSA to cognitive decline [5, 13].

Epidemiologic evidence indicates an association between OSA and increased dementia risk, although effect sizes differ depending on cohort characteristics, diagnostic approaches, and confounding variables. In a large UK general‐practice electronic health record cohort, OSA was linked to a higher risk of both all‐cause dementia and vascular dementia. However, individuals receiving treatment did not exhibit an increased dementia risk compared with matched controls. These findings underscore the importance of treatment patterns and illustrate the limitations of residual confounding in observational studies [300].

Biomarker and neuroimaging studies add biological plausibility by linking OSA to Alzheimer's disease (AD)‐related pathology and impaired brain clearance pathways. A 2023 systematic review and meta‐analysis reported that adults with OSA had lower cerebrospinal fluid Aβ42/Aβ40 and higher amyloid PET burden and t‐tau/Aβ42 ratio compared with controls, consistent with a shift toward AD‐like biomarker profiles in at least a subset of patients [301].

Recent longitudinal imaging studies indicate that OSA may affect neurovascular and glymphatic‐related biomarkers. In a 2025 neurology cohort, CPAP therapy was associated with altered trajectories of progression of enlarged perivascular spaces, findings consistent with the hypothesis that effective treatment can influence sleep‐dependent brain fluid clearance pathways [302].

Sleep microstructure may provide an additional layer of risk stratification. A 2024 sleep review emphasized that, beyond AHI, disrupted microarchitecture and arousal characteristics may better predict subsequent cognitive impairment, aligning with the broader move toward physiological phenotyping in OSA [303].

Taken together, the evidence supports a model in which OSA contributes to both functional daytime impairment and long‐term neurodegenerative risk through interacting hypoxic, sleep‐fragmentation, vascular, and clearance‐pathway mechanisms. Clinically, these data strengthen the rationale for proactive identification and effective treatment of OSA in patients with cognitive complaints or high dementia risk, while highlighting the need for trials enriched for high physiological burden and with cognition‐relevant outcomes [300, 303].

An earlier CSF/PET‐focused meta‐analysis found abnormal AD‐related CSF biomarkers and increased PET amyloid burden in OSA, reinforcing plausibility for accelerated preclinical neurodegenerative signatures in susceptible individuals [304]. At the population level, a systematic review/meta‐analysis (*N* > 1.3 million) reported increased risk of incident neurocognitive disorder and AD in sleep apnea, while associations with vascular dementia were less consistent [305]. Interventional evidence remains mixed: a pilot randomized crossover CPAP trial in OSA with mild cognitive impairment reported limited short‐term cognitive benefit, and a broader RCT meta‐analysis found insufficient evidence of cognitive improvement despite reduced sleepiness [306, 307].

### 4.2 Emerging Organ‐Specific Structural Remodeling Potentially Linked to Fibrosis

Four distinct evidence layers are relevant for organ‐specific remodeling: (i) cell and animal studies, which establish mechanistic plausibility; (ii) human cross‐sectional associations, which identify cosegregation but are susceptible to confounding and reverse causality; (iii) human longitudinal associations, which offer stronger temporal inference; and (iv) interventional evidence, which is necessary to substantiate disease‐modifying claims. For most organs addressed in this review, the evidence is most robust at the mechanistic and associative levels, while longitudinal and interventional data remain limited, heterogeneous, and inconsistent. Within this framework, the lung, liver, kidney, and heart are discussed as organ systems where fibrosis‐linked remodeling most directly supports the central thesis. In contrast, cystic fibrosis (CF) and non‐CF bronchiectasis are presented as related chronic airway remodeling conditions within a broader structural remodeling context. These conditions do not have fibrosis‐equivalent evidentiary status compared with IPF, MASLD/metabolic dysfunction‐associated steatohepatitis (MASH), kidney, or heart, but they illustrate how OSA‐related sleep‐disordered breathing and nocturnal hypoxemia may plausibly exacerbate pre‐existing airway remodeling, inflammatory burden, and maladaptive repair in structurally vulnerable airways remodeling, inflammatory burden, and maladaptive repair in structurally vulnerable airways.

#### 4.2.1 Lungs and IPF

OSA is highly prevalent among individuals with IPF. Recent meta‐analyses consistently report pooled OSA prevalences of approximately 70–76%, although substantial heterogeneity exists across studies and mild‐to‐moderate OSA predominates in many cohorts [103, 308]. In selected cohorts of patients with fibrotic diffuse parenchymal lung disease or IPF, prevalence estimates may be even higher, with one study reporting an OSA occurrence rate of 83.3% [309]. Despite this strong association, important uncertainties remain regarding the determinants of OSA in IPF and its long‐term implications [310].

In contemporary cohorts, clinical outcomes are influenced less by the presence of OSA, as defined by the AHI, and more by the extent of nocturnal hypoxemia. In a large single‐center cohort, OSA was identified in 64.7% of patients with IPF. However, mortality risk correlated more strongly with the duration of time spent below 90% oxygen saturation (T90%) than with the diagnosis of OSA itself [102]. Similarly, in progressive fibrotic ILD, prolonged nocturnal hypoxemia has been associated with accelerated symptom progression and increased short‐term mortality [311]. Observational cohorts further suggest that sleep‐related breathing disorders in IPF and ILD are associated with pulmonary vascular involvement and adverse clinical outcomes [312, 313].

Restrictive lung physiology in IPF may contribute directly to OSA susceptibility. Reduced lung volumes diminish caudal traction on the upper airway, thereby increasing airway collapsibility during sleep. In fibrotic ILD cohorts, TLC has emerged as an independent predictor of OSA risk, supporting a “lung‐volume” mechanism that operates alongside classical OSA risk factors such as age, male sex, and adiposity [312, 314]. Clinically, this framework helps explain why OSA may develop in patients with IPF even in the absence of severe obesity and reinforces the rationale for proactive screening strategies rather than relying solely on symptom‐based detection.

From a mechanistic standpoint, intermittent hypoxemia associated with OSA and the chronic hypoxemia characteristic of IPF are biologically poised to converge on shared pathways of lung injury and fibrotic repair. These include OS, epithelial cell damage, macrophage‐driven inflammation, ER stress, and activation of profibrotic signaling networks, including TGF‐β signaling, EMT, and EndMT. Experimental studies using bleomycin‐induced pulmonary fibrosis models demonstrate that superimposed IH—mimicking OSA—exacerbates lung inflammation, OS, and collagen deposition [20, 315]. Moreover, ER‐stress‐dependent pathways have been implicated in hypoxia‐amplified apoptosis and fibrotic remodeling [273]. Importantly, experimental interruption of IH attenuates fibrosis severity and reduces macrophage accumulation within fibrotic regions, supporting the concept that IH acts as a modifiable amplifier of pulmonary fibrogenesis [219].

Evidence from human imaging studies further suggests that sleep‐related hypoxia may contribute to early trajectories of ILD. Longitudinal computed tomography (CT) analyses in a large community‐based cohort from the MESA showed that hypoxic burden associated with obstructive respiratory events correlated with progressive increases in interstitial lung density and accelerated declines in lung volumes and forced vital capacity (FVC), independently of BMI and other anthropometric variables [316]. Conversely, population‐based analyses indicate that IPF itself may increase the subsequent risk of developing OSA, supporting the concept of bidirectional interactions between restrictive parenchymal remodeling and sleep‐disordered breathing [317].

Collectively, these findings support the systematic evaluation of sleep‐disordered breathing in patients with IPF and underscore the importance of quantifying nocturnal hypoxemia using metrics such as the oxygen desaturation index (ODI), T90%, and nadir SpO_2_, in addition to AHI, when stratifying risk and tailoring therapy. This approach aligns with guideline‐based recommendations that emphasize the identification and management of comorbidities, including OSA, as integral components of comprehensive IPF care, together with antifibrotic therapy and supportive interventions [318]. A recent position statement from the Thoracic Society of Australia and New Zealand similarly underscores the importance of comorbidity assessment and nonpharmacologic management strategies in the multidisciplinary treatment of IPF and progressive pulmonary fibrosis, identifying OSA and nocturnal hypoxemia as actionable clinical targets [319].

Therapeutic evidence regarding treatment of OSA in IPF remains largely observational but is gradually expanding. A prospective pilot study in newly diagnosed IPF patients initiating antifibrotic therapy demonstrated that CPAP and/or nocturnal oxygen therapy were feasible, generally well tolerated, and associated with high adherence and improved polysomnographic indices over 1 year. In addition, selected circulating biomarkers, including MMP‐1, CRP, and surfactant protein‐D, showed favorable changes in relevant subgroups, although lung function parameters did not clearly improve over the same interval [25]. Similarly, a PRISMA‐informed systematic review of ILD–OSA cohorts reported potential CPAP‐associated benefits on quality of life and exacerbation‐related outcomes, although the impact on mortality remains uncertain due to heterogeneity in diagnostic criteria, treatment adherence, and study design [320].

Earlier cohort studies have also described improvements in daytime sleepiness, fatigue, and sleep quality following CPAP therapy in patients with IPF, with adherence‐dependent signals suggesting a possible survival benefit. However, these findings remain susceptible to confounding and require confirmation in adequately powered randomized trials incorporating robust clinical endpoints such as exacerbation frequency, decline in FVC, or transplantation‐free survival [321, 322].

Emerging biomarker research further suggests the existence of physiology‐linked endotypes within IPF populations affected by OSA. Bronchoalveolar lavage concentrations of KL‐6, as well as circulating levels of endothelin‐1 (ET‐1) and S100A9, have been reported to correlate with indices of nocturnal oxygenation and desaturation burden in patients with IPF and coexisting OSA. These observations raise the possibility that biomarker‐based stratification could help identify individuals most likely to benefit from targeted interventions aimed at mitigating sleep‐related hypoxemia [323].

Despite these advances, important IPF‐specific uncertainties remain. Standardized strategies for screening for OSA and nocturnal hypoxemia in patients with IPF remain lacking, and prospective studies are needed to disentangle the relative contributions of AHI, hypoxemia burden, and sleep fragmentation to disease severity and progression. Likewise, interventional studies should determine whether treatment of sleep‐disordered breathing modifies fibrotic biology or clinical trajectory in IPF beyond improving symptoms, sleep quality, and daytime functioning. Overall, current evidence supports OSA as a plausible modifier of disease progression in IPF and other fibrotic ILDs, with nocturnal hypoxemia burden emerging as a more informative signal than AHI alone. However, causal inference remains limited by the predominance of observational data and substantial confounding from restrictive lung mechanics, baseline gas‐exchange abnormalities, and nocturnal desaturation intrinsic to fibrotic lung disease. Within this framework, OSA and nocturnal hypoxemia are clearly prevalent in IPF and fibrotic ILD. Hypoxemia‐focused metrics are increasingly recognized as having greater prognostic relevance than event counts alone. Furthermore, it is biologically and clinically plausible that OSA‐related hypoxemic stress may exacerbate pulmonary injury in certain patients. However, whether treating OSA alters fibrosis progression remains uncertain, as current interventional evidence is limited and does not yet support conclusions about disease modification.

#### 4.2.2 Liver

Steatotic liver disease (SLD) is the updated umbrella term encompassing fatty liver disorders, with MASLD and MASH replacing the previously used terms NAFLD and NASH in contemporary classification frameworks [324]. OSA, which is highly prevalent among individuals with cardiometabolic risk factors, is increasingly recognized as a potential codriver of MASLD/MASH progression through mechanisms including CIH, sleep fragmentation, and sustained sympathetic activation [325].

Clinical and population‐based studies support an association between OSA‐related nocturnal hypoxemia and liver injury or fibrotic risk. Early investigations reported correlations between sleep oxygen saturation indices and circulating markers of hepatic fibrosis in patients with OSA [326]. More recently, analyses of the NHANES 2017–2020 cohort showed that self‐reported sleep apnea was associated with increased liver stiffness and a higher prevalence of ultrasound‐defined liver fibrosis (14.0 vs. 7.3%), even after adjustment for metabolic confounders [327]. Across community and clinic‐based cohorts, indices reflecting the burden of nocturnal hypoxemia—such as time spent with oxygen saturation below 90% (T90/Tc90), ODI, mean or nadir SpO_2_, and composite hypoxic burden metrics—appear to correlate more consistently with hepatic injury and fibrosis risk than apnea–hypopnea frequency alone [328, 329].

Studies involving biopsy‐proven NAFLD populations further indicate that OSA and nocturnal hypoxemia are associated with greater histological liver damage and more advanced fibrosis, supporting biological plausibility beyond shared obesity‐related risk [330]. However, causal inference remains challenging. Mendelian randomization analyses have reported weak or absent direct genetic evidence for a causal relationship between OSA and NAFLD after accounting for metabolic traits, suggesting that the association may be mediated or confounded by adiposity and insulin resistance [331].

Epidemiologically, MASLD/NAFLD is common among individuals with OSA, with prevalence estimates frequently ranging between 44 and 50%, and even higher rates reported in obese populations at high risk of OSA [332, 333, 334]. This association is largely mediated by insulin resistance, a central metabolic disturbance in OSA. Insulin resistance enhances adipose tissue lipolysis and increases circulating free fatty acids, thereby augmenting hepatic lipid influx and facilitating progression from simple steatosis toward inflammatory injury and fibrotic remodeling [325, 334]. Controlled human exposure studies provide further mechanistic support for an IH–lipotoxicity axis: repeated nocturnal hypoxia increases sympathetic activity, stimulates adipose lipolysis, and reduces insulin‐mediated suppression of circulating free fatty acids [335].

Mechanistically, CIH can function as an independent “second hit” that accelerates hepatic steatosis and fibrogenesis. CIH promotes systemic and hepatic insulin resistance, increases adipose‐derived lipid flux, and induces mitochondrial ROS generation and ER stress, ultimately triggering hepatocyte injury and inflammatory signaling [325, 336]. Hypoxia‐responsive metabolic pathways also influence hepatic lipid handling. In murine models, IH altered hepatic regulation of the fatty acid transporter CD36, leading to patterns of triglyceride accumulation and inflammatory injury that differed between lean and diet‐induced obese states [337].

At the tissue level, prolonged IH alone has been shown to activate hepatic inflammatory programs resembling transcriptomic signatures observed in human steatohepatitis, including innate immune and TNF‐related signaling pathways [220]. Experimental work in diet‐induced obesity models further demonstrates that CIH promotes hepatic fibrogenesis by activating the TLR4/MyD88/MAPK/NF‐κB signaling cascade, thereby linking hypoxia‐induced inflammation with collagen deposition [338].

Fibrotic amplification likely reflects coordinated interactions among hepatocytes, Kupffer cells/macrophages, and hepatic stellate cells (HSCs). CIH can impair Kupffer cell autophagy via the CX3CL1/CX3CR1–CaMKIIδ/HDAC4/Rubicon signaling axis, promoting Kupffer‐cell apoptosis and inflammatory remodeling within the hepatic microenvironment [339]. Concurrently, hypoxia‐responsive signaling within HSCs may directly enhance fibrogenic activity. Activation of the HIF‐1α/IL‐6 axis in HSCs has been associated with a profibrotic environment characterized by IL‐17A signaling [340]. In patients with severe obesity and OSA, circulating lysyl oxidase—a hypoxia‐inducible enzyme involved in collagen cross‐linking—has been reported to be elevated and associated with markers of liver fibrosis, suggesting a potential hypoxia‐sensitive biomarker axis [341].

Recent experimental studies have also highlighted the contribution of regulated cell death and DNA damage responses in CIH‐related liver injury. In a pair‐feeding CIH model of NASH, IH triggered selective autophagy‐mediated degradation of the DNA repair enzyme EEPD1, resulting in increased hepatocyte DNA damage and fibrosis markers. Pharmacological stabilization of EEPD1 using retigabine mitigated these pathological changes [342]. Complementary evidence indicates that IH‐driven macrophage IL‐6 signaling can promote hepatocyte ferroptosis through ubiquitination‐mediated suppression of GPX4, providing another mechanistic pathway linking inflammation and fibrogenesis [343].

The gut–liver axis may represent an additional amplifier of hypoxemia‐related liver injury. OSA has been associated with alterations in the intestinal microbiome and biomarkers indicative of impaired intestinal barrier integrity. These changes may facilitate endotoxin translocation and enhance hepatic Toll‐like receptor signaling during chronic hypoxemia [344].

From a translational perspective, polysomnographic indices of nocturnal hypoxemia may help identify patients who require further evaluation for MASLD/NAFLD in OSA clinics. Prospective and elastography‐based studies suggest that metrics such as Tc90/T90 and ODI may serve as pragmatic triggers for transient elastography (FibroScan) screening of hepatic steatosis and fibrosis in patients with OSA [329, 345]. However, current evidence does not support CPAP as a stand‐alone antifibrotic therapy for MASLD in the context of OSA. In a large sham‐controlled randomized trial, 6 months of auto‐adjusting CPAP improved nocturnal respiratory indices but did not significantly improve hepatic steatosis or fibrosis compared with subtherapeutic CPAP [346]. Similarly, a recent systematic review found that CPAP therapy may yield modest, inconsistent reductions in aminotransferase levels and minor metabolic improvements. However, randomized trials have not demonstrated meaningful effects on liver stiffness or hepatic fat accumulation [26].

Consequently, current clinical practice favors an integrated approach in which evaluation and treatment of OSA are combined with aggressive management of obesity, dyslipidemia, and insulin resistance. Guideline‐aligned noninvasive fibrosis stratification strategies—such as initial assessment using the FIB‐4 score followed by elastography—are recommended to identify patients with advanced fibrosis and guide appropriate referral and management pathways [347]. Collectively, current evidence supports OSA as a plausible modifier of MASLD/MASH progression, with the strongest signal emerging in patients with substantial nocturnal hypoxemia and pre‐existing metabolic vulnerability. Nevertheless, interpretation remains complicated by major confounders and overlapping causal pathways, including obesity, visceral adiposity, insulin resistance, and Type 2 diabetes. In this context, it is well established that OSA frequently coexists with MASLD/MASH, and that hypoxemia‐related indices often correlate more closely with markers of hepatic injury and fibrosis risk than AHI alone. It is also biologically and clinically plausible that OSA‐related hypoxemic stress acts as a cofactor that exacerbates liver injury and fibrogenic susceptibility in metabolically vulnerable individuals. By contrast, whether OSA‐directed therapy alone can meaningfully reverse hepatic fibrosis remains uncertain, as current interventional evidence is heterogeneous and insufficient to support CPAP as a stand‐alone antifibrotic strategy. Clinically, these data support the use of OSA‐related hypoxemia as a cue to intensify liver risk stratification and integrated cardiometabolic care, rather than as evidence that airway therapy alone will reverse hepatic fibrosis.

#### 4.2.3 Kidneys

Direct epidemiological estimates of OSA specifically in cohorts with biopsy‐proven or imaging‐defined renal fibrosis remain limited. Nevertheless, across the broader spectrum of CKD, OSA is common and may contribute to accelerated renal injury and fibrogenic remodeling. A recent systematic review and meta‐analysis including both nondialysis and dialysis CKD populations reported that OSA affects more than one‐third of individuals with CKD and is associated with increased mortality in these patients [100]. In a large sleep‐clinic cohort (*n* = 1295), moderate‐to‐severe OSA was associated with approximately 2.6–3.0‐fold higher odds of belonging to the moderate‐to‐very‐high KDIGO risk categories for CKD progression—based on combined estimated glomerular filtration rate (eGFR) and albuminuria criteria—even after adjustment for major CKD risk factors [348, 349]. Population‐based analyses further support this association, showing that individuals at high risk of OSA exhibit higher prevalence of albuminuria and CKD, with particularly strong associations observed in men [350].

Beyond the traditional apnea–hypopnea frequency metrics, nocturnal hypoxemic burden appears to play a particularly important role in renal outcomes. In hypertensive patients undergoing polysomnographic evaluation, both OSA severity and hypoxemia‐related indices—such as the ODI and the time spent with oxygen saturation below 90% (T90)—were independently associated with clinically significant proteinuria [351]. Similarly, a prospective cohort study involving individuals with Type 2 diabetes demonstrated that elevated T90 values identified patients more likely to progress to very‐high‐risk CKD categories during follow‐up [352]. Supporting the concept that nocturnal hypoxemia may induce early tubular stress, studies have also reported increased urinary concentrations of kidney injury molecule‐1 in patients with OSA. Importantly, this biomarker may increase before detectable abnormalities in conventional markers such as eGFR decline or albuminuria [353].

From a mechanistic standpoint, IH induces repetitive ischemia–reoxygenation injury in the kidney, an organ inherently susceptible to hypoxia because of its distinctive microvascular structure and substantial metabolic requirements. These fluctuations in oxygen levels intensify OS, promote endothelial dysfunction, and activate inflammatory signaling pathways, collectively driving profibrotic molecular responses. TGF‐β1 is recognized as a key regulator of renal fibrogenesis, directing myofibroblast activation and ECM deposition via both canonical and noncanonical signaling mechanisms [354].

Recent experimental studies provide direct evidence linking CIH to fibrotic remodeling in the kidney. CIH exposure has been shown to promote renal macrophage infiltration and induce MMT, a process strongly associated with ECM accumulation and fibrosis. MR activation appears to play a key mechanistic role in this pathway, as pharmacological MR blockade with eplerenone significantly attenuated CIH‐induced fibrotic changes in experimental models [24]. In parallel, CIH can activate inflammatory cell death pathways in renal tubular epithelial cells. Experimental data demonstrate that CIH induces tubular pyroptosis through activation of the NLRP3 inflammasome, whereas pharmacological inhibition of NLRP3 mitigates renal injury in vivo [355].

Hemodynamic alterations associated with OSA may further exacerbate hypoxia‐driven renal injury. Experimental models show that CIH can promote glomerular hyperfiltration and intensify hypoxia‐induced reductions in renal perfusion and tissue oxygen tension, thereby worsening intrarenal hypoxic stress [356]. These hemodynamic and inflammatory mechanisms together create a microenvironment conducive to progressive renal fibrosis.

From a therapeutic perspective, whether treatment of OSA with CPAP confers meaningful renoprotective benefits remains an open question. In a 52‐week randomized clinical trial involving patients with diabetic kidney disease and coexisting OSA, CPAP therapy did not produce a statistically significant overall reduction in albuminuria. However, patients with higher adherence to CPAP therapy exhibited greater long‐term reductions in albuminuria compared with usual care [27]. Similarly, a recent systematic review and meta‐analysis concluded that current evidence, characterized by low‐to‐moderate certainty, suggests that CPAP therapy has little or no significant effect on slowing the decline of renal function. These findings highlight the need for larger randomized controlled trials with longer follow‐up durations and improved phenotyping based on nocturnal hypoxemic burden and baseline CKD risk [357].

Given the emerging mechanistic links between CIH, MR activation, and inflammasome‐driven tubular injury, future therapeutic strategies may need to adopt a combined approach. Potential strategies include reducing nocturnal hypoxemia through optimized CPAP adherence or alternative OSA therapies, in conjunction with targeted pharmacological modulation of profibrotic pathways, such as MR antagonists or anti‐inflammatory interventions [24, 355].

Current clinical evidence demonstrates an association between OSA, particularly its nocturnal hypoxemia component, and markers of kidney injury, such as albuminuria, proteinuria, increased risk of CKD progression, and early tubular injury biomarkers. Preclinical studies further support biologically plausible mechanisms linking IH to renal fibrogenesis via MR‐dependent MMT, inflammasome activation, and renal hypoperfusion. However, direct human evidence connecting OSA with quantitatively measured renal fibrotic burden, including histological fibrosis, validated imaging surrogates, or fibrosis‐specific biomarker panels, remains limited. Future research should prioritize these endpoints to establish causality and identify patient subgroups most likely to benefit from antifibrotic and OSA‐targeted therapeutic strategies [100, 354]. Collectively, current renal evidence supports a biologically plausible link between OSA and kidney injury, with stronger support for renal risk enrichment and early injury signals than for direct demonstration of OSA‐driven renal fibrosis in humans. Causal interpretation is limited by substantial confounding, including the bidirectional relationship between OSA and CKD, as well as comorbidities such as hypertension, diabetes, obesity, fluid‐retention states, and pre‐existing renal impairment. Within this context, it is well established that OSA, particularly when characterized by significant nocturnal hypoxemia, is associated with albuminuria, proteinuria, increased risk of CKD, and early tubular stress biomarkers. IH is also biologically plausible as a promoter of renal fibro‐inflammatory remodeling through oxidative, inflammatory, hemodynamic, and mineralocorticoid‐related mechanisms. In contrast, the extent to which OSA directly induces quantifiable renal fibrosis in humans, and whether OSA‐directed therapy alone can slow fibrotic renal remodeling, remains uncertain due to limited direct fibrosis‐specific evidence in humans. Clinically, nocturnal hypoxemia in OSA should be interpreted as an indication to intensify CKD risk stratification and renal monitoring, rather than as evidence that airway‐directed therapy alone will alter renal fibrotic progression.

#### 4.2.4 Heart

OSA is increasingly recognized as a driver of adverse cardiac remodeling, and myocardial fibrosis represents a plausible structural intermediate linking nocturnal respiratory events to heart failure and malignant arrhythmias [12, 358]. In hypertrophic obstructive cardiomyopathy and other scar‐prone cardiac phenotypes, myocardial fibrosis, as measured by late gadolinium enhancement percentage (LGE%), increases in parallel with OSA severity, indicating a significant association between sleep‐disordered breathing and structural myocardial remodeling [359]. However, contemporary cardiovascular magnetic resonance (CMR) analyses highlight considerable heterogeneity across OSA phenotypes and comorbidity profiles. These studies recommend combining LGE with native T1 and extracellular volume (ECV) mapping to capture both replacement fibrosis and diffuse interstitial remodeling [360].

Evidence from AF cohorts further supports the role of sleep‐related hypoxemia in cardiac structural remodeling. In patients with AF, greater IH severity is associated with a larger left‐atrial low‐voltage area, an electroanatomical substrate closely linked to atrial fibrosis, even before overt left‐atrial enlargement becomes evident [361]. Conversely, studies examining relatively “pure” OSA populations without major cardiometabolic disease have produced different findings. In a comprehensive contrast‐enhanced CMR study of such patients, LGE was absent, and the ECV fraction was not elevated, suggesting that early OSA‐related myocardial remodeling may initially manifest as cellular hypertrophy and functional impairment rather than as established fibrotic replacement [362].

IH, a hallmark of OSA, has been strongly implicated in structural and functional cardiac remodeling. Experimental and clinical data indicate that IH promotes biventricular hypertrophy, myocardial fibrosis, chamber dilation, and reduced stroke volume, thereby providing mechanistic insight into the development of heart failure in patients with OSA [363]. More recent epidemiological work has emphasized the importance of hypoxemia depth–duration metrics, such as hypoxic burden, which predict incident cardiovascular disease and mortality in large community cohorts, including MESA and MrOS. These metrics outperform traditional frequency‐based indices such as the AHI in cardiovascular risk stratification, reinforcing the concept that nocturnal hypoxemia is a major driver of cardiac remodeling and heart failure progression [8]. Complementary perspectives argue that hypoxic burden captures clinically meaningful aspects of nocturnal hypoxemia that are not fully represented by AHI or ODI, thereby providing a more mechanism‐aligned framework linking OSA severity with OS, inflammation, and structural tissue remodeling [203].

At the molecular level, IH activates key transcriptional regulators, including HIF‐1α and NF‐κB, triggering systemic inflammation and cardiovascular stress. These signaling pathways promote the release of proinflammatory mediators, including TNF‐α, IL‐6, and CRP. The resulting inflammatory milieu contributes to endothelial dysfunction, vascular injury, and atherosclerosis, thereby exacerbating myocardial damage and fibrotic remodeling [364]. Across experimental and translational syntheses, recurrent hypoxia–reoxygenation cycles are increasingly conceptualized as a form of chronic cardiometabolic “stress testing,” amplifying sympathetic activation, renin–angiotensin–aldosterone system signaling, and redox–inflammatory interactions. These interconnected networks converge on canonical profibrotic pathways, including TGF‐β/SMAD signaling, myofibroblast differentiation, and ECM accumulation [12, 206].

Mechanical stress represents an additional pathophysiological driver of cardiac remodeling in OSA. Repeated inspiratory efforts against an occluded upper airway generate large negative intrathoracic pressures, reaching approximately −65 mmHg. These pressure swings impair diastolic function, increase left ventricular wall stress, and compromise cardiac filling, all of which contribute to fibrotic remodeling [365]. Importantly, these mechanical forces act synergistically with hypoxemia and hypercapnia to promote atrial stretch, increase ventricular afterload, and activate mechano‐transduction pathways in fibroblasts and endothelial cells. This combination lowers the threshold for maladaptive cardiac remodeling, particularly in hypertensive heart disease, where OSA functions as a potent “second hit” on myocardial structure and function [12, 366].

Experimental models provide further evidence supporting the fibrotic impact of OSA. Chronic exposure to recurrent apneas has been shown to induce atrial fibrosis in animal models, with increased collagen deposition and structural remodeling observed in the atria of rats exposed to simulated OSA conditions [367]. More recent mechanistic work has expanded these findings by identifying interorgan regulatory pathways. For example, short‐term chronic IH induces senescent remodeling in epididymal white adipose tissue, coupled with cardiac interstitial fibrosis, highlighting a potential adipose–heart crosstalk mechanism in OSA‐associated fibrogenesis [211]. In murine models of pressure overload, CIH accelerates systolic dysfunction and increases myocardial fibrosis by promoting HIF‐1α‐dependent EndMT. Notably, upregulation of the HIF‐1α inhibitor PHD3 attenuates EndMT and collagen deposition, providing causal evidence for hypoxia‐responsive vascular–fibroblast signaling in cardiac remodeling [368].

At the cellular level, IH can directly promote myofibroblast differentiation and excessive ECM production by activating the HIF‐1α–TGF‐β/SMAD signaling axis. This pathway provides a direct molecular bridge between hypoxemia‐driven signaling and matrix deposition in the myocardium [18]. In addition, mitochondrial dysfunction and impaired mitophagy have emerged as important contributors to CIH‐driven myocardial injury. Experimental studies have demonstrated that berberine preserves mitochondrial biogenesis and PINK1–Parkin‐dependent mitophagy through SIRT6–AMPK–FOXO3a signaling, thereby reducing OS and attenuating cardiac remodeling in CIH models [369].

The temporal dimension of IH exposure also appears to influence cardiac remodeling trajectories. Long‐term IH exposure across an aging timescale has been shown to accelerate cardiovascular aging, impair both systolic and diastolic function, and increase mortality in experimental models. These findings suggest that cumulative hypoxemic stress may progressively shift early hypertrophic adaptation toward overt cardiac dysfunction and fibrosis [210].

Beyond ventricular remodeling, atrial fibrosis represents a key intermediate phenotype linking OSA with AF. Clinical–experimental studies demonstrate that increasing OSA severity correlates with atrial structural remodeling and inflammatory activation in patients. Correspondingly, lean mice exposed to IH develop atrial fibrosis accompanied by increased expression of TGF‐β and collagen genes, reinforcing the causal role of CIH independent of obesity [221]. These mechanistic insights align with contemporary concepts recognizing sleep‐disordered breathing as a modifiable contributor to arrhythmia risk through structural and electrophysiological remodeling [221].

From a clinical perspective, the phenotype‐dependent nature of myocardial remodeling suggests that therapeutic benefits are most likely when OSA treatment is initiated early and maintained with high adherence, particularly in patients with elevated hypoxic burden and coexisting hypertension or structural heart disease. Evidence syntheses indicate that CPAP therapy improves biventricular myocardial mechanics across echocardiographic studies, supporting partial reversibility of subclinical cardiac dysfunction [370]. An updated systematic review and meta‐analysis (10 studies including 385 CPAP‐treated patients) demonstrated that CPAP therapy significantly improved left ventricular and right ventricular global longitudinal strain (LV‐GLS and RV‐GLS), reduced pulmonary artery systolic pressure, and decreased right atrial volume index. However, no significant changes were observed in conventional measures such as left ventricular ejection fraction, tricuspid annular plane systolic excursion, left ventricular mass, or *E*/*e*′ ratio, indicating that myocardial strain represents a more sensitive marker of CPAP‐responsive remodeling [371].

Advanced imaging and biomarker studies further suggest that obesity modifies the cardiac response to PAP therapy, highlighting the need for individualized risk–benefit assessment and careful phenotyping in clinical trials [372]. In line with these findings, recent cardiovascular society guidance recommends systematic assessment and management of sleep‐disordered breathing in patients with cardiovascular disease, with particular attention to symptom burden, nocturnal hypoxemia, and treatment adherence [373].

Current evidence indicates that OSA‐related myocardial fibrosis is a heterogeneous process influenced by the interaction of hypoxemia burden, mechanical stress, and underlying cardiometabolic comorbidities. Future research that incorporates refined OSA severity metrics, such as hypoxic and ventilatory burden, alongside tissue‐based endpoints, such as LGE and T1/ECV mapping, may more effectively identify patient subgroups in whom OSA treatment can attenuate fibrotic cardiac remodeling. The literature consistently supports a biologically plausible role for OSA in adverse cardiac remodeling; however, the available human studies are too heterogeneous to confirm a single, uniform fibrosis pathway across the entire OSA spectrum. Confounding factors, including obesity, hypertension, AF burden, pre‐existing structural heart disease, and the complex interplay between hypoxemic load and negative intrathoracic pressure stress, limit causal inference. It is well established that OSA is associated with adverse myocardial and atrial remodeling and the development of an arrhythmogenic substrate. Hypoxemia‐related metrics frequently provide more informative structural risk assessment than AHI alone. It is also plausible that OSA amplifies myocardial and atrial fibrotic vulnerability in susceptible phenotypes, particularly when intermittent hypoxemia, mechanical stress, and underlying cardiovascular disease are present. However, whether this results in a uniform OSA‐driven cardiac fibrosis pathway in the general population, or whether OSA treatment can reverse established myocardial fibrosis, remains uncertain due to the heterogeneity and phenotype‐dependence of current evidence. Clinically, these findings support moving beyond AHI‐only characterization in patients with OSA and cardiovascular disease, emphasizing hypoxemia burden, remodeling‐prone phenotypes, and timely, adherent treatment for those at the highest structural risk.

#### 4.2.5 Related Chronic Airway Remodeling Settings: CF and Non‐CF Bronchiectasis

CF and non‐CF bronchiectasis are related chronic airway remodeling settings rather than core fibrosis‐dominant organ systems. Their inclusion is intended to broaden the structural‐remodeling framework of OSA, rather than to suggest that the evidence linking OSA to fibrosis‐specific airway remodeling is comparable to that available for the lung, liver, kidney, or heart. A high prevalence of OSA has been reported in children and adolescents with CF, regardless of age or baseline lung function, underscoring the importance of systematically assessing sleep‐disordered breathing during clinical evaluation, even in patients with apparently preserved pulmonary function [374]. In a systematic review and meta‐analysis of seven observational PSG‐based studies, most of which were cross‐sectional, the pooled prevalence of OSA was approximately 65% when defined as an AHI greater than 1 event per hour and 52% when defined as an AHI greater than 2 events per hour. However, these studies were limited by small sample sizes and insufficient control for confounding variables, highlighting the need for more robust prospective PSG‐based cohorts to generate more reliable prevalence estimates and improve risk stratification [374].

Recent pediatric‐focused reviews emphasize that sleep disturbances in CF are multifactorial and arise from a combination of factors, including chronic cough, pain, airway infection and inflammation, gas‐exchange abnormalities, and upper‐airway disease. Because symptoms such as snoring or daytime sleepiness may be insensitive indicators of sleep‐disordered breathing, these findings support maintaining a low threshold for objective sleep assessment through PSG in CF populations [375, 376]. In the era of highly effective CFTR modulators, emerging clinical observations suggest potential shifts in sleep‐breathing phenotypes. For example, a single‐center retrospective PSG series including 49 children with CF (2012–2023) reported OSA in 57% of patients and identified higher odds of OSA among those receiving highly effective modulator therapy (odds ratio 4.3; 95% CI 1.2–14.9). Nevertheless, confounding factors such as age differences and referral bias remain plausible explanations for this association [377].

In adults with CF, in‐laboratory PSG studies also reveal a substantial burden of sleep‐disordered breathing and nocturnal hypoxemia that is not reliably predicted by routine clinical indicators. In one open‐access cohort of 52 adults with CF, the average AHI was 4.5 ± 4.0 events per hour, yet 40% of participants met diagnostic criteria for OSA (AHI > 5/h) and 25% exhibited clinically significant nocturnal hypoxemia. Importantly, commonly used clinical predictors, including BMI, lung function parameters, age, and Epworth Sleepiness Scale scores, showed limited ability to discriminate individuals with OSA [378]. Similar findings emerged from a registry‐linked PSG analysis conducted in Toronto that included 42 adults with CF. In this cohort, OSA represented the most frequent sleep breathing disorder (64.3%), accompanied by sustained nocturnal hypoxemia (16.7%) and sleep‐related hypoventilation (9.5%). Notably, only 41% of individuals with elevated AHI received PAP therapy, and corticosteroid use was the only factor significantly associated with the presence of sleep breathing disorders (OR 5.0), highlighting a potential gap in clinical management [379].

CFTR modulator therapy may also influence sleep‐disordered breathing patterns. In a prospective pre–post PSG study in adults with CF, initiation of the triple combination therapy elexacaftor/tezacaftor/ivacaftor resulted in reductions in AHI and oxygen desaturation indices—including during REM sleep—without significant alterations in overall sleep architecture. These findings suggest that improvements in airway physiology and gas exchange mediated by CFTR modulation may translate into measurable benefits in sleep‐related respiratory parameters [380].

Upper‐airway disease is a significant contributor to sleep disturbances in CF. Chronic rhinosinusitis (CRS) is highly prevalent in individuals with CF and can lead to nasal obstruction and sleep fragmentation. However, the severity of structural CRS does not necessarily correlate with OSA severity. In a cohort of adults with CF who underwent both PSG and sinus CT, radiologic evidence of CRS was present in all participants, and 48% demonstrated an AHI of 5 or greater per hour. Despite this, CT‐based CRS severity scores did not correlate with PSG‐derived sleep parameters, indicating that PSG screening should be considered for all individuals with CF, regardless of CRS burden [381, 382].

At the same time, the introduction of highly effective CFTR modulator therapies has been associated with a nutritional transition in CF populations, particularly in adults. Registry‐based and prospective studies indicate that BMI frequently increases following initiation of elexacaftor/tezacaftor/ivacaftor therapy, with weight gain largely driven by increases in fat mass rather than fat‐free mass. This shift in body composition may increase upper‐airway collapsibility and ventilatory load during sleep, thereby potentially increasing susceptibility to OSA [383, 384].

From a mechanistic perspective, both CF and bronchiectasis are characterized as chronic airway‐remodeling disorders rather than fibrosis‐dominant phenotypes [385]. Their inclusion lies in the broader concept that sleep‐disordered breathing and intermittent hypoxemia can exacerbate pre‐existing airway injury, mucus stasis, infection‐driven inflammation, and maladaptive repair, thereby worsening an airway environment already prone to remodeling [385]. In this context, sleep‐disordered breathing may develop through several interacting mechanisms, such as persistent airway infection and inflammation, mucus hypersecretion and airway obstruction, nocturnal cough and pain, ventilatory impairment of either obstructive or restrictive type, upper‐airway involvement, and the presence of evolving comorbidities [385]. In this setting, sleep‐disordered breathing may emerge through multiple interacting pathways, including persistent airway infection and inflammation, mucus hypersecretion and airway plugging, nocturnal cough and pain, obstructive or restrictive ventilatory impairment, upper‐airway involvement, and evolving comorbidities [375, 376].

IH associated with sleep‐disordered breathing may further intensify oxidative and inflammatory signaling, thereby contributing to airway remodeling and disease progression, although biomarker‐driven mechanistic studies specifically examining these processes in CF remain limited [385].

In non‐CF bronchiectasis (NCFB), PSG‐based studies similarly report a high prevalence of OSA. In patients with clinically stable NCFB, OSA was identified in approximately 40.8% of individuals, and infection with *Pseudomonas aeruginosa* was associated with higher AHI values, more severe oxygen desaturation indices, and lower nadir oxygen saturation during sleep [386]. Another cohort study of adults with NCFB reported an OSA prevalence of 55.8%, with most cases classified as mild, and an increasing frequency observed with advancing age [387].

Direct comparative studies between CF‐associated bronchiectasis and NCFB also suggest broadly similar prevalence of sleep‐disordered breathing. In a cohort study including 35 adults with CF bronchiectasis and 35 with NCFB, OSA was detected in 53% of all participants, with comparable prevalence between CF and NCFB groups (54 vs. 51%). Male sex and longer disease duration emerged as independent risk factors for OSA, while CF patients demonstrated lower mean and nadir nocturnal oxygen saturation compared with individuals with NCFB [388].

**FIGURE 4:**
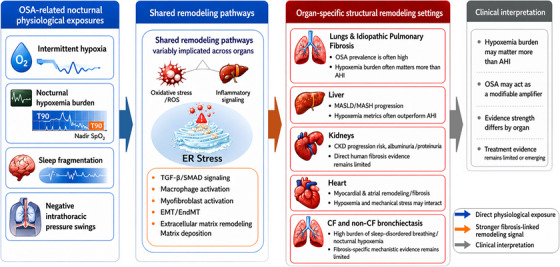
Emerging fibrosis‐linked structural remodeling in obstructive sleep apnea: shared pathways, organ‐specific contexts, and clinical interpretation. This figure summarizes an organ‐oriented framework through which OSA‐related nocturnal physiological exposures may contribute to emerging fibrosis‐linked structural remodeling beyond established systemic complications. Intermittent hypoxia, cumulative nocturnal hypoxemia burden, sleep fragmentation, and negative intrathoracic pressure swings converge on shared remodeling pathways that are variably implicated across organs, including oxidative stress/ROS, inflammatory signaling, ER stress, TGF‐β/SMAD signaling, macrophage activation, myofibroblast activation, EMT/EndMT, and extracellular matrix remodeling and deposition. These maladaptive repair programs may be expressed across organ‐specific contexts, including lungs and idiopathic pulmonary fibrosis, liver MASLD/MASH progression, CKD‐associated kidney remodeling, and myocardial or atrial remodeling/fibrosis. CF and non‐CF bronchiectasis are included as boundary chronic airway‐remodeling phenotypes within the broader structural‐remodeling framework, not as settings with established fibrosis‐specific OSA causality. The figure emphasizes four interpretive principles: hypoxemia burden may be more informative than AHI alone; OSA may act primarily as a modifiable amplifier of fibrotic vulnerability rather than as an isolated primary cause; the strength of mechanistic and clinical evidence differs across organs; and treatment evidence for organ‐level remodeling remains limited or emerging. Blue arrows indicate OSA‐related physiological exposure; orange arrows indicate fibrosis‐linked remodeling signals; gray arrows indicate clinical interpretation. *Abbreviations*: AHI, apnea–hypopnea index; CF, cystic fibrosis; CKD, chronic kidney disease; EMT, epithelial‐to‐mesenchymal transition; EndMT, endothelial‐to‐mesenchymal transition; ER, endoplasmic reticulum; IPF, idiopathic pulmonary fibrosis; MASLD, metabolic dysfunction‐associated steatotic liver disease; MASH, metabolic dysfunction‐associated steatohepatitis; OSA, obstructive sleep apnea; ROS, reactive oxygen species; SMAD, small mothers against decapentaplegic; SpO_2_, peripheral oxygen saturation; T90, percentage of sleep time spent with oxygen saturation below 90%; TGF‐β, transforming growth factor beta.

Taken as a whole, current evidence is strongest for a substantial burden of sleep‐disordered breathing and nocturnal hypoxemia in CF and non‐CF bronchiectasis. On the other hand, direct evidence linking OSA to fibrosis‐specific airway remodeling remains limited. Major confounders, including chronic infection, airway inflammation, cough burden, corticosteroid exposure, CRS, altered body composition, and the evolving effects of CFTR modulator therapy, further complicate interpretation. What is well established is that routine clinical predictors may underestimate clinically relevant sleep‐related respiratory burden in these populations. What is biologically and clinically plausible is that OSA‐related intermittent hypoxemia may exacerbate airway stress, inflammatory burden, and overall clinical burden in selected patients with chronic airway‐remodeling disorders. By contrast, a direct fibrosis‐specific role of OSA in long‐term airway structural remodeling, as well as any structural airway benefit from OSA treatment, remains speculative. From a clinical perspective, these conditions are most appropriately considered chronic airway remodeling contexts that warrant a low threshold for objective sleep assessment, rather than fibrosis‐dominant organ models such as IPF, MASLD/MASH, kidney, or heart. Figure 4 presents the organ‐specific fibrosis‐associated remodeling contexts and their unified clinical interpretation. Table 3 presents a concise, organ‐specific synthesis of the current evidence base, including the predominant evidence type, the most informative OSA exposure metrics, major confounders, overall strength of inference, and organ‐level benefit associated with OSA therapy.

**TABLE 3:** Organ‐specific evidence grading across fibrosis‐linked and related structural remodeling settings in obstructive sleep apnea.

Organ system	Dominant evidence type	Main OSA exposure metric used	Major confounders	Overall strength of evidence	Organ‐level benefit shown with CPAP/other OSA therapy?
Lung/IPF‐fibrotic ILD	Mechanistic and human cross‐sectional/retrospective evidence predominate [103, 308]. Prospective data remain limited [320]. Interventional data remain limited [320].	T90, ODI, and nadir SpO_2_ capture clinically relevant nocturnal hypoxemia more directly than AHI [102]. Broader nocturnal hypoxemia burden metrics may therefore be more informative than AHI alone in IPF‐fibrotic ILD settings [311, 312, 313].	Restrictive physiology and baseline gas‐exchange impairment complicate inference [20, 219, 312, 314, 315]. Pulmonary hypertension, age, and adiposity add further confounding [310].	Association‐level evidence is moderate [103, 308]. Causal inference remains low [103, 308]. Interventional disease‐modifying evidence remains low [320].	No definitive organ‐level benefit has been shown [320]. Current signals relate mainly to symptoms, adherence, and selected quality‐of‐life or exacerbation outcomes [321, 322].
Liver/MASLD–MASH	Mechanistic and cross‐sectional data predominate [326, 328, 329, 330]. Biopsy‐based associations have been reported [326, 328, 329, 330]. Longitudinal data remain limited [346]. Randomized interventional data remain heterogeneous [26].	T90/Tc90 and ODI are often more informative than AHI alone [328, 329]. Mean or nadir SpO_2_ and composite hypoxic‐burden metrics may also capture risk more directly than event counts alone [328, 329].	Obesity and visceral adiposity are major confounders [331]. Insulin resistance and Type 2 diabetes also act as major confounders or mediators [325, 336, 338, 339, 340].	Association‐level evidence is moderate [331]. Causal inference remains low‐to‐moderate [346]. Interventional evidence for fibrosis regression remains low [26].	CPAP alone has not shown consistent fibrosis regression [346]. Interventional findings remain heterogeneous and insufficient for disease‐modifying claims [26].
Kidney/CKD context	Preclinical mechanistic evidence is strong [100, 354]. Human data are stronger for kidney injury than for direct fibrosis‐specific endpoints [100, 354].	T90 and ODI are informative clinical exposure metrics [352]. Broader nocturnal hypoxemia measures may also outperform event counts alone in CKD settings [351].	Hypertension, diabetes, and obesity complicate inference [100, 354]. Fluid retention and the bidirectional CKD–OSA relationship add further confounding [100, 354].	Human evidence for fibrosis‐specific inference remains low to moderate [100, 354].	Organ‐level renoprotective benefit remains uncertain [357]. Adherence‐dependent signals have been reported [27]. Robust disease modification remains unproven [357].
Heart/myocardial and atrial remodeling	Mechanistic and imaging/electrophysiologic association studies support plausibility [12, 358, 359, 361]. Human phenotype data remain heterogeneous [12, 358, 359, 361].	Hypoxic burden, ODI, and nocturnal hypoxemia appear more informative than AHI [7, 8, 203]. LGE/T1/ECV phenotyping may refine cardiac fibrosis assessment [360].	Obesity, hypertension, AF burden, and pre‐existing structural heart disease complicate inference [372]. The relative contributions of hypoxemic versus pressure stress remain variable across phenotypes [373].	Mechanistic plausibility is moderate [12, 358, 359, 360, 361]. Clinical fibrosis‐specific inference remains only low‐to‐moderate [12, 358, 359, 360, 361].	No definitive fibrosis reversal has been shown [370, 371, 373]. Some subclinical remodeling and strain/function measures may improve with early, adherent treatment [370, 371, 373].
CF/non‐CF bronchiectasis	Evidence is strongest for a high burden of sleep‐disordered breathing [374, 378]. Fibrosis‐specific inference remains weak [386, 388].	AHI should be interpreted alongside nocturnal hypoxemia indices [386, 388]. Clinically relevant desaturation burden may occur even when OSA is not severe, as assessed by AHI alone [378, 381, 382, 386].	Chronic infection/inflammation and cough burden complicate inference [375, 376]. CRS, corticosteroid exposure, and CFTR modulators add further confounding [383, 384]. Altered body composition may also influence observed associations [381, 382].	Overall evidence for fibrosis‐specific OSA inference remains low [385].	No proven structural airway benefit has been shown with OSA therapy [378, 381, 382, 386]. A low threshold for objective sleep assessment remains appropriate in symptomatic or high‐risk patients [378, 381, 382, 386].

This table summarizes the current evidence linking obstructive sleep apnea (OSA) to organ‐specific structural remodeling settings potentially involving fibrotic pathways. For each organ context, it identifies the dominant type of evidence, the OSA exposure metric that appears most informative, the major confounders, the overall current strength of evidence, and whether organ‐level benefit has been demonstrated with CPAP or other OSA‐directed therapy. The “overall strength of evidence” column provides a narrative synthesis of mechanistic, cross‐sectional, longitudinal, and interventional data rather than a formal guideline‐based grade. The lung, liver, kidney, and heart are presented as the organ systems most closely aligned with the fibrosis‐linked thesis of this review. In contrast, cystic fibrosis (CF) and non‐CF bronchiectasis are included as related chronic airway remodeling settings for contextual comparison, not as fibrosis‐dominant organ models with equivalent evidentiary status.

*Abbreviations*: AF, atrial fibrillation; AHI, apnea–hypopnea index; CF, cystic fibrosis; CKD, chronic kidney disease; CPAP, continuous positive airway pressure; ECV, extracellular volume; ILD, interstitial lung disease; IPF, idiopathic pulmonary fibrosis; LGE, late gadolinium enhancement; MASLD, metabolic dysfunction‐associated steatotic liver disease; MASH, metabolic dysfunction‐associated steatohepatitis; non‐CF, non‐cystic fibrosis; ODI, oxygen desaturation index; OSA, obstructive sleep apnea; PSG, polysomnography; SpO_2_, peripheral oxygen saturation; T1, native T1 mapping; T90, percentage of sleep time spent with oxygen saturation <90%; Tc90, cumulative time spent with oxygen saturation <90%.

## 5 Diagnosis and Management

Mechanistic and complication evidence related to OSA informs practical diagnostic and therapeutic decisions. Diagnosis begins with clinical suspicion and objective sleep testing, and may include assessment of physiological burden, phenotypic heterogeneity, and targeted anatomical or endotype‐based evaluations. OSA management can be organized by clinical maturity: established therapies such as PAP, MADs, weight management, positional therapy, and selected surgical or device‐based interventions; phenotype‐guided adjunctive treatments; investigational pharmacological options; and careful review of fibrosis‐related findings. This framework differentiates routine sleep medicine from specialized or investigational care, emphasizing the need to integrate OSA treatment with cardiometabolic and organ‐specific risk management, rather than focusing solely on AHI reduction.

### 5.1 Diagnosis

#### 5.1.1 Diagnostic Principles

**FIGURE 5:**
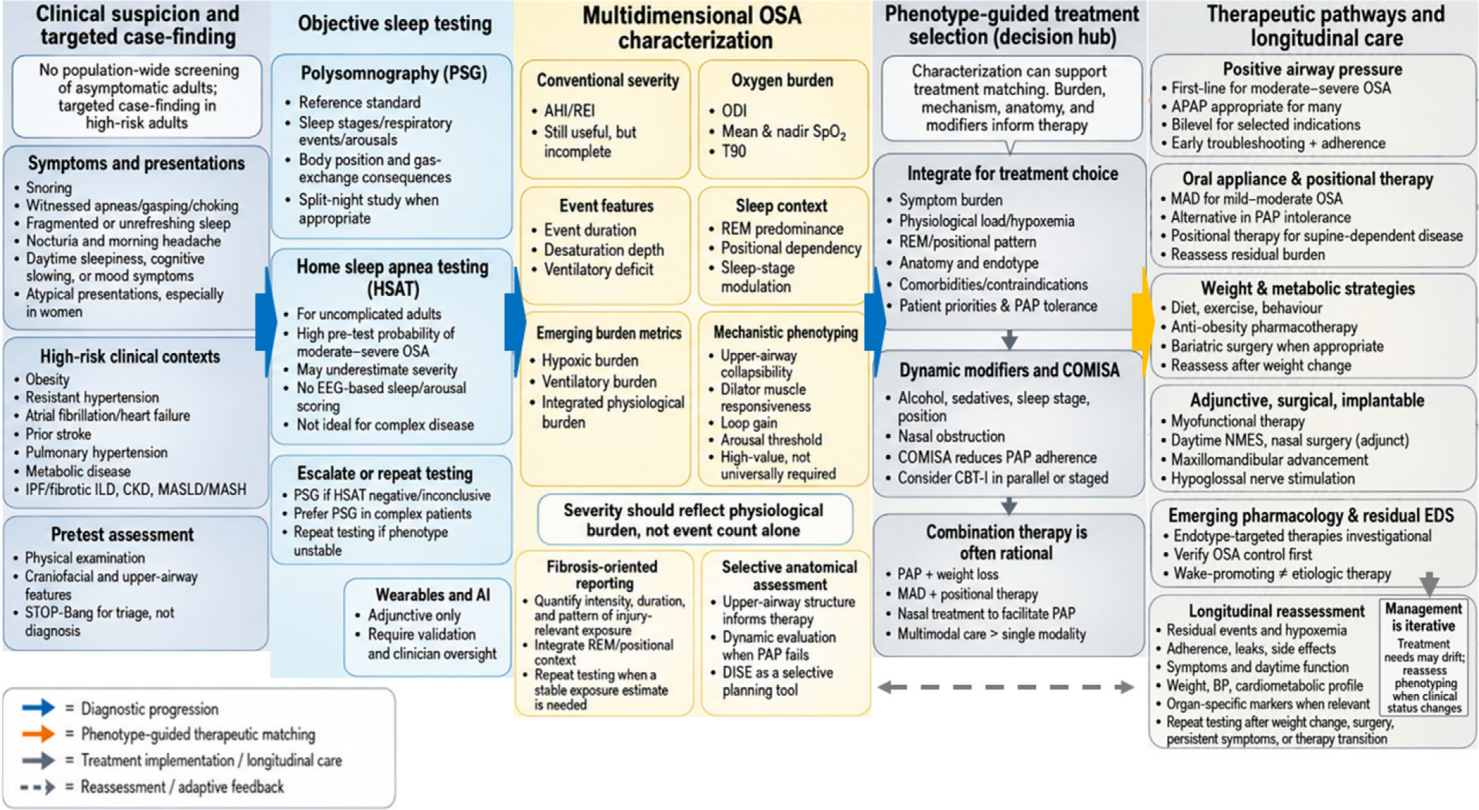
Phenotype‐guided diagnosis and management of obstructive sleep apnea: a multidimensional and adaptive framework. This figure outlines a contemporary phenotype‐guided approach to OSA, moving beyond event‐frequency definitions toward multidimensional and adaptive care. Evaluation starts with clinical suspicion and case‐finding, followed by objective sleep testing with polysomnography (PSG) or home sleep apnea testing (HSAT), according to clinical context. OSA characterization integrates conventional event‐frequency metrics with oxygen burden, event features, sleep‐stage and positional context, emerging burden metrics, fibrosis‐oriented exposure reporting, and selective anatomical or mechanistic phenotyping. These dimensions may refine treatment selection, although routine care still relies mainly on established clinical assessment, objective testing, and treatment pathways. Management extends beyond PAP)to include oral appliance and positional therapy, weight and metabolic strategies, adjunctive, surgical, or implantable interventions, and, when indicated, investigational pharmacological approaches or wake‐promoting therapy for residual excessive daytime sleepiness after adequate OSA control has been confirmed. Dynamic modifiers, including alcohol, sedatives, sleep stage, body position, nasal obstruction, and comorbid insomnia and sleep apnea (COMISA), may influence disease expression, tolerance, and adherence, often supporting combination therapy. Longitudinal reassessment of events, hypoxemia, adherence, symptoms, daytime function, cardiometabolic profile, and organ‐specific markers enables iterative treatment refinement. Effective OSA treatment may attenuate upstream hypoxemia, sleep fragmentation, and pressure stress, but established fibrotic remodeling may require organ‐specific management. In high‐risk phenotypes, OSA control should be integrated with respiratory, cardiometabolic, and, when relevant, antifibrotic or anti‐inflammatory care. Blue arrows indicate diagnostic progression; orange arrows indicate phenotype‐guided therapeutic matching; gray arrows indicate treatment implementation and longitudinal care; dashed gray arrows indicate reassessment and adaptive feedback. *Abbreviations*: HSAT, home sleep apnea testing; MAD, mandibular advancement device; OSA, obstructive sleep apnea; PAP, positive airway pressure; T90, percentage of sleep time spent with oxygen saturation below 90%.

OSA is highly prevalent, with global burden estimates approaching one billion affected adults [1]. Nevertheless, clinically meaningful disease remains substantially underdiagnosed because symptom burden, physiological burden, and comorbidity profiles are heterogeneous across affected populations [60, 389]. Diagnosis should therefore not be conceptualized merely as confirmation of an AHI threshold, but rather as a staged process integrating clinical suspicion and objective sleep testing, with a multidimensional appraisal of nocturnal physiological burden serving as a complementary layer that may refine interpretation in selected contexts rather than replacing routine diagnostic pathways (Figure 5) [390, 391].

This broader diagnostic framing has important implications for case identification. In adults without recognized symptoms, current evidence remains insufficient to support population‐wide screening [389]. By contrast, targeted case‐finding is particularly relevant in patients with resistant hypertension, AF, heart failure, pulmonary hypertension, or prior stroke, among whom OSA prevalence is high, and under‐recognition remains common [13]. Thus, contemporary diagnosis should be understood as a process of identifying clinically relevant disease beyond respiratory event frequency alone, while recognizing that full biologically enriched phenotyping is not yet uniformly embedded in routine decision pathways.

#### 5.1.2 Clinical Evaluation and Pretest Probability

A structured clinical evaluation should document habitual snoring, witnessed apneas, gasping or choking, fragmented or unrefreshing sleep, nocturia, morning headache, and daytime sequelae such as sleepiness, cognitive slowing, or mood disturbance [390]. However, classical male‐pattern presentations do not capture the full phenotypic spectrum. Women are often symptomatic at lower AHI values and may report sleep fragmentation, poor sleep quality, fatigue, insomnia‐like complaints, or mood symptoms more prominently than overt witnessed apneas or marked sleepiness. REM‐predominant OSA is also more common in women, which may reduce recognition when pretest evaluation relies too heavily on traditional symptom profiles [60].

Pretest probability also rises substantially in the presence of obesity, resistant hypertension, AF, heart failure, prior stroke, and metabolic dysfunction [13, 390]. Physical examination remains clinically informative because BMI, neck circumference, nasal obstruction, tonsillar size, tongue position, and oropharyngeal crowding help contextualize both diagnostic probability and downstream treatment options [390, 392]. Screening tools such as STOP‐Bang retain value for triage rather than diagnosis. In a meta‐analysis of clinical cohorts, pooled STOP‐Bang sensitivity in sleep‐clinic populations reached 92, 95, and 96% for AHI thresholds of ≥5, ≥15, and ≥30 events/h, respectively, whereas specificity remained at only 35, 27, and 28%, respectively [393]. Similar perioperative data support the same overall pattern of high sensitivity but modest specificity [394]. Accordingly, questionnaires and prediction algorithms should be regarded as tools to support case‐finding, not as substitutes for PSG or HSAT [390].

#### 5.1.3 Objective Testing

In‐laboratory PSG remains the reference standard for diagnosis because it simultaneously quantifies sleep stages, respiratory events, arousals, body position, and gas‐exchange consequences. When technically feasible and clinically appropriate, split‐night protocols may also expedite the transition from diagnosis to treatment.

By contrast, HSAT is evidence‐based only for uncomplicated adults with a high pretest probability of moderate‐to‐severe OSA [391]. Because most HSAT systems estimate respiratory event frequency over monitoring time rather than true sleep time, disease severity may be underestimated, particularly when sleep efficiency is low or when arousal‐linked events materially contribute to disease expression [395, 396]. Simplified testing is also less appropriate when central sleep apnea, sleep‐related hypoventilation, parasomnias, or severe insomnia are part of the differential diagnosis [390].

Current guidance recommends PSG if a single HSAT is negative, inconclusive, or technically inadequate despite ongoing clinical suspicion. PSG is also preferred for patients with significant cardiorespiratory disease, suspected hypoventilation, neuromuscular weakness, chronic opioid use, prior stroke, or severe insomnia [390]. Diagnostic classification also depends on the hypopnea scoring rule used, as the AASM‐recommended ≥3% desaturation and/or arousal definition identifies a broader range of disease than the more restrictive ≥4% desaturation‐only rule [396].

Diagnostic timing is also important. In medically hospitalized adults at increased risk for OSA, testing should ideally be undertaken after medical stabilization or after discharge rather than during acute unstable illness [397]. Repeat testing can also be justified in selected cases, as recent data demonstrate substantial night‐to‐night variability not only in AHI but also in hypoxic burden, flow limitation, and derived physiological endotypes [117, 390].

#### 5.1.4 Beyond the AHI

Although the AHI is widely used in clinical practice and trial design, it reduces diverse respiratory events to a single frequency metric and does not fully account for event duration, desaturation depth, cumulative airflow reduction, or the sleep‐stage and positional context of events [391]. This limitation is clinically relevant because patients with similar AHI values may have markedly different exposure to IH and therefore substantially different downstream cardiovascular risk profiles [8, 118].

For this reason, contemporary interpretation should extend beyond the AHI alone and report ODI, mean and nadir oxygen saturation, time spent below 90% saturation (T90), event duration, REM predominance, and positional dependency whenever available [391, 398]. Among emerging alternatives, hypoxic burden integrates the depth and duration of respiratory event‐related desaturation and has shown stronger prognostic associations than AHI for cardiovascular outcomes [118, 399]. In a large cohort analysis, sleep apnea‐specific hypoxic burden predicted incident heart failure in men even when AHI did not [400].

Ventilatory burden provides complementary information by quantifying the cumulative magnitude of airflow reduction across the night and has independently predicted both all‐cause and cardiovascular mortality [7]. In population‐based analyses, hypoxic and ventilatory burdens predicted incident cardiovascular disease and mortality, whereas arousal burden did not, underscoring that not all PSG‐derived summary metrics carry equivalent prognostic value [8]. Taken together, these measures better approximate the biological dose of nocturnal injury than event counts alone [118].

#### 5.1.5 Physiologic Phenotyping and Anatomical Assessment

OSA is mechanistically heterogeneous, arising from variable contributions of upper‐airway collapsibility, impaired pharyngeal dilator‐muscle compensation, unstable ventilatory control with high loop gain, and arousal‐threshold characteristics [112]. This heterogeneity has intensified interest in PSG‐derived or model‐based endotyping as a framework for matching therapy to dominant mechanisms rather than relying exclusively on anatomy‐severity labels [112].

However, recent ATS guidance emphasizes that translation of endotyping into routine large‐scale practice still requires technical standardization, external validation, reproducibility data, clinically meaningful cut‐offs, and prospective impact studies [401]. Accordingly, endotyping is currently best viewed as a high‐value phenotyping strategy rather than a universally required diagnostic step [112, 401].

Anatomical assessment becomes particularly relevant when PAP is poorly tolerated or when surgery or HNS is being considered [402, 403]. In this context, DISE remains a valuable dynamic tool for identifying the level and pattern of collapse and for tailoring upper‐airway intervention [402]. Nevertheless, current outcome data remain mixed, and DISE should therefore be used selectively as a planning tool rather than as a universal diagnostic requirement for all adults with OSA [403, 404].

#### 5.1.6 Portable Monitors, Wearables, and AI‐enabled Diagnostics

Efforts to expand diagnostic capacity have accelerated the development of multisensor portable systems and wearables that incorporate airflow, respiratory effort, oximetry, PAT, photoplethysmography, acoustic signals, and algorithmic analysis [395, 405]. Importantly, adding more physiological parameters to a portable monitor does not necessarily improve diagnostic performance, and devices using apparently similar signals may still vary substantially because of differences in sensor quality, signal processing, event definitions, and proprietary algorithms [395].

From 2019 to 2023, nine novel wearables were cleared by the US FDA for home diagnosis of adult OSA, spanning photoplethysmography/PAT‐based, acoustic, and respiratory‐effort‐based platforms [405]. These systems may also support the development of non‐AHI metrics and allow for repeated longitudinal assessments beyond a single laboratory night. However, these features should be considered promising additions rather than replacements for established clinician‐supervised diagnostic pathways [395].

Current evidence does not yet support the routine stand‐alone clinical use of wearable or AI‐enabled diagnostic systems. These approaches should be considered adjunctive, center‐dependent, or research‐focused rather than standard care. Meta‐analytic data are encouraging, with pooled sensitivity and specificity for sleep apnea detection at 0.938 and 0.752, respectively. However, the pooled accuracy for severity classification remains lower at 0.651 [406]. Important limitations include the need for independent validation, cross‐device comparability, artifact handling, and robust performance in central sleep apnea, hypoventilation, or multimorbid populations [405, 406]. Clinician‐supervised interpretation and escalation to PSG remain therefore essential whenever simplified test results are discordant with the clinical picture or when the patient is medically complex [390].

#### 5.1.7 Fibrosis‐Oriented Diagnostic Perspectives

Within a fibrosis‐oriented or structural‐remodeling framework, the established clinical priority remains the diagnosis and characterization of OSA through standard sleep‐medicine pathways. At the same time, additional quantification of the intensity, duration, and pattern of nocturnal exposure most plausibly linked to chronic tissue injury may be especially valuable in translational studies, specialized centers, and selected high‐risk clinical contexts [8, 118]. At present, however, these complementary metrics should be regarded as adjunctive rather than defining, and should not be interpreted as constituting a routine fibrosis‐oriented or endotype‐based diagnostic standard of care across general sleep‐medicine practice [8, 118]. AHI or REI alone often provides an incomplete representation of nocturnal physiological burden because it does not adequately capture hypoxia–reoxygenation cycling or cumulative ventilatory stress [7, 400].

Metrics such as T90, mean and nadir oxygen saturation, hypoxic burden, and ventilatory burden may more accurately reflect the physiological aspects of nocturnal injury most relevant to OS, inflammatory activation, endothelial dysfunction, and end‐organ remodeling [118, 398]. Accordingly, in translational, organ‐oriented, or specialized‐center settings, sleep reports should consider pairing conventional severity categories with a multidimensional description of physiological burden, sleep‐stage dependency, and, when available, positional context. This approach represents an enhanced reporting strategy rather than a universal requirement [8, 391]. Repeat testing may be appropriate when phenotypic stability is uncertain or when mechanistic inference requires a stable estimate of nocturnal exposure [117, 401].

Table 4 presents a clinically and mechanistically oriented diagnostic framework that emphasizes the shift from an exclusively AHI‐based classification toward multidimensional assessment of physiological burden and heterogeneity.

**TABLE 4:** Diagnostic framework of obstructive sleep apnea: integrating AHI, physiological burden, and phenotypic heterogeneity.

Diagnostic domain	Core synthesis	Practical implications for diagnosis/reporting
Diagnostic principles and case identification	Diagnosis should be framed as a staged process, not a simple AHI‐threshold confirmation [390, 391]. Symptom burden, physiological burden, and comorbidity patterns are heterogeneous across patients [1].	Routine screening of asymptomatic adults is not supported [60, 389]. Targeted case‐finding is more appropriate in high‐risk cardiovascular/metabolic settings [13].
Clinical evaluation and pretest probability	History should capture snoring, witnessed apneas, gasping/choking, nonrestorative sleep, nocturia, morning headache, daytime sleepiness, cognitive symptoms, and mood disturbance [390]. Women may present with lower‐AHI, insomnia‐like, or REM‐predominant phenotypes [60].	STOP‐Bang is useful for triage, not diagnosis [393]. Physical examination and comorbidity profiling remain important for estimating probability and anticipating therapy [390].
Objective testing: PSG, HSAT, scoring rules, and repeat studies	PSG remains the reference standard [390]. HSAT is best reserved for uncomplicated adults with high pretest probability of moderate‐to‐severe OSA and can underestimate severity when sleep time is not directly measured [395, 396].	Reports should specify the scoring rule used, and PSG should be preferred when HSAT is negative or inconclusive, but suspicion persists or when relevant comorbidities are present [390]. Repeat testing is justified when night‐to‐night variability is clinically important [117, 390].
Beyond the AHI: multidimensional physiological burden	AHI alone does not capture event duration, desaturation depth, cumulative airflow reduction, or REM/positional context [8, 118, 391]. ODI, T90, oxygenation metrics, hypoxic burden, and ventilatory burden better approximate nocturnal injury dose [118, 391, 398, 399].	When available, non‐AHI burden metrics can complement standard reporting, because similar AHI values can mask very different cardiovascular and remodeling risk profiles; their use may be especially informative in translational studies and in selected high‐risk clinical contexts [7].
Physiologic phenotyping and anatomical assessment	OSA reflects variable contributions from collapsibility, dilator‐muscle compensation, loop gain, and arousal threshold [112]. Endotyping and anatomical assessment can refine mechanism‐based treatment selection, but at present, these approaches are considered as selective or center‐specific tools rather than uniformly implemented components of routine diagnosis [112, 401].	Endotyping is promising but not yet a universally required clinical step [112, 401]. DISE should be used selectively when surgery or HNS is being considered [402, 403, 404].
Portable monitors, wearables, and AI‐enabled diagnostics	Portable multisensor systems and wearables may expand access to home testing, but performance varies with sensors, signal processing, event definitions, and proprietary algorithms [395, 405].	AI‐assisted wearables remain clinically supportive rather than stand‐alone tools [406]. Clinician‐supervised interpretation and escalation to PSG remain essential in discordant or complex cases [390].
Fibrosis‐oriented and remodeling‐oriented diagnostic perspective	For mechanistic and remodeling studies, diagnosis should quantify the intensity, duration, and pattern of nocturnal exposure most plausibly linked to chronic tissue injury [7, 8, 118, 400]. AHI and REI alone are often insufficient [118, 398].	In translational studies and selected high‐risk clinical contexts, reports may usefully pair conventional severity categories with T90, oxygenation metrics, hypoxic burden, ventilatory burden, and positional or REM context [8, 390].

This table summarizes the current diagnostic framework for obstructive sleep apnea (OSA), integrating clinical suspicion, screening tools, objective sleep testing, physiological burden, phenotypic heterogeneity, and emerging digital and artificial intelligence (AI)‐assisted approaches. It highlights the limitations of relying solely on the apnea–hypopnea index (AHI) and emphasizes the complementary value of multidimensional metrics that better reflect disease complexity, biological burden, and clinical relevance.

*Abbreviations*: OSA, obstructive sleep apnea; AHI, apnea–hypopnea index; PSG, polysomnography; HSAT, home sleep apnea testing; HNS, hypoglossal nerve stimulation; STOP‐Bang, snoring, tiredness, observed apnea, high blood pressure, body mass index, age, neck circumference, and gender; ODI, oxygen desaturation index; T90, percentage of sleep time spent with oxygen saturation <90%; REM, rapid eye movement; REI, respiratory event index; DISE, drug‐induced sleep endoscopy; AI, artificial intelligence.

### 5.2 Management

#### 5.2.1 Treatment Principles

The management of OSA should be contextualized within a clearly defined hierarchy of evidence and clinical maturity [13, 407]. Established clinical practice encompasses therapies with recognized roles in routine care, including PAP‐based treatment, oral appliance therapy for appropriate candidates, weight‐management strategies, positional therapy for selected phenotypes, and selected surgical or device‐based interventions when clinically indicated [407, 408, 409, 410, 411, 412, 413, 414]. The next tier consists of phenotype‐guided adjunctive strategies, which are increasingly valuable when tailored to positional dependence, obesity, craniofacial anatomy, COMISA, PAP tolerance, or dominant mechanistic traits; however, their benefit remains highly dependent on appropriate patient selection [411, 413, 414, 415]. A further tier includes investigational or mechanism‐based approaches that are not yet routine and should not be regarded as equivalent to standard care [158, 416, 417, 418]. Fibrosis‐oriented implications are clinically relevant but remain hypothesis generating. Although suppression of nocturnal hypoxemia and related physiological stress is biologically important, disease‐modifying effects on established organ fibrosis have not been demonstrated [25, 419, 420]. In clinical practice, treatment success should be evaluated primarily against established clinical targets, such as symptom improvement, objective control of respiratory events and hypoxemia, adherence, and cardiometabolic risk reduction. Interpretation of fibrosis‐oriented outcomes should remain cautious and context specific [13, 408, 415, 421].

IH and arousal‐related sympathetic activation are mechanistically associated with OS, inflammatory signaling, endothelial dysfunction, and the activation of profibrotic pathways. Nevertheless, OSA therapy should not currently be considered an established antifibrotic intervention, as direct evidence demonstrating that OSA treatment alone reverses established fibrosis remains limited. The most defensible interpretation is that early and effective management of sleep‐disordered breathing may mitigate ongoing biological injury, but should be combined with organ‐specific, disease‐modifying therapies in high‐risk populations such as those with IPF and MASLD [322, 420]. Supporting this perspective, pilot studies in IPF suggest that CPAP, with or without supplemental nocturnal oxygen, is feasible and generally well tolerated; however, current studies are insufficiently powered to determine whether these interventions affect lung function decline or fibrotic remodeling. Similarly, hepatic improvements observed in MASLD/NAFLD should be considered adjunctive and hypothesis generating, rather than independently disease‐modifying [25, 419, 420].

Treatment selection should therefore be individualized within established clinical pathways, while mechanistically informed phenotyping should be considered as a valuable adjunct rather than a universally available prerequisite for care. Integrating overall disease severity and physiological load, including AHI or REI, oxygen desaturation burden, time spent below 90% oxygen saturation, and REM‐ or supine‐predominance, with symptom phenotype, patient priorities, anatomical and nonanatomical traits such as pharyngeal collapsibility, dilator muscle responsiveness, loop gain, and arousal threshold, as well as comorbidities and contraindications, is essential [13, 407]. Dynamic modifiers such as alcohol, sedatives, sleep stage, body position, nasal obstruction, and coexisting insomnia should also be considered, as they can significantly alter both disease expression and treatment tolerability. Consequently, phenotype‐ and endotype‐informed approaches have become increasingly influential in contemporary OSA frameworks, although bedside implementation remains inconsistent and often center specific [415, 421]. Coexisting insomnia warrants explicit attention, as COMISA is common, reduces PAP acceptance and adherence, and may require parallel or staged cognitive behavioral therapy for insomnia to optimize overall therapeutic effectiveness [422, 423].

For most adults with moderate‐to‐severe OSA, PAP remains first‐line therapy. However, non‐PAP approaches may be appropriate when the disease is mild, strongly positional, craniofacially driven, obesity‐related, or marked by persistent PAP intolerance. Because OSA is rarely the consequence of a single biological driver, combination therapy is increasingly rational, for example, PAP plus weight loss, mandibular advancement plus positional therapy, or nasal intervention to facilitate PAP use [408, 411]. This multimodal logic is supported by recent evidence syntheses indicating that cointerventions may improve physiological control or adherence when a single modality does not sufficiently address all relevant mechanisms, even though PAP still provides the greatest average reduction in respiratory events overall [415, 424].

#### 5.2.2 PAP Therapy

Among established standard therapies, PAP remains the reference treatment for most adults with moderate‐to‐severe OSA because it pneumatically splints the upper airway and can normalize breathing and oxygenation across sleep stages and body positions. Current AASM guidance supports treatment initiation through either in‐laboratory titration or home‐based strategies, including auto‐adjusting PAP (APAP), coupled with early follow‐up and objective monitoring of residual events, mask leak, and adherence [408]. APAP is therefore an appropriate frontline delivery strategy for many patients. On the other hand, bilevel PAP is generally reserved for specific situations such as pressure intolerance, unusually high pressure requirements, or coexisting hypoventilation, rather than being viewed as intrinsically superior in otherwise uncomplicated cases [408, 421].

In practice, however, the effectiveness of PAP depends less on physiological efficacy than on sustained adherence. Common barriers include mask discomfort, leak, nasal obstruction or dryness, pressure intolerance, insomnia, aerophagia, and claustrophobia. Evidence‐based mitigation strategies include interface optimization, heated humidification, treatment of nasal disease, behavioral coaching, telemonitoring, and management of comorbid insomnia [408]. Early troubleshooting is particularly important. Telemedicine‐based follow‐up modestly but consistently improves nightly PAP use, and interface choice is clinically relevant: compared with nasal masks, oronasal masks tend to require higher pressures, leave higher residual AHI, and are associated with poorer adherence. Nasal surgery should therefore be framed primarily as an adherence‐facilitating adjunct in appropriately selected patients rather than as a stand‐alone cure for OSA [425, 426, 427]. Recent implementation frameworks further emphasize that remote CPAP monitoring is best embedded within phenotype‐sensitive pathways rather than used as a fully stand‐alone strategy, because patients with persistent symptoms, COMISA, major mask‐related problems, or evolving comorbidity often require in‐person multidisciplinary reassessment. Notably, adherence trajectories appear to stabilize early, with high use in the first month strongly predicting continued use over 2 years, underscoring the importance of intensive troubleshooting immediately after treatment initiation [428, 429].

PAP consistently improves daytime sleepiness and quality of life, and its blood‐pressure‐lowering effect is most evident in patients with resistant or uncontrolled hypertension [13, 285]. Importantly, the field has moved beyond a simplistic interpretation of cardiovascular trials as merely positive or negative. Intention‐to‐treat benefit is often diluted by suboptimal use. In contrast, adherence‐sensitive analyses indicate lower risks of recurrent cardiovascular events, all‐cause mortality, and cardiovascular mortality when PAP is used adequately [289, 430].

In blood‐pressure terms, the average effect is usually modest rather than dramatic. Nevertheless, it remains clinically meaningful in high‐risk phenotypes, particularly in patients with higher baseline BP and more consistent nightly use [13, 285].

#### 5.2.3 Established Non‐PAP Alternatives and Metabolic Therapies

Custom, titratable MADs represent an established alternative for treating mild‐to‐moderate OSA and serve as a reasonable rescue strategy for selected patients with more severe disease who are unable to tolerate PAP. Meta‐analytic evidence demonstrates significant reductions in AHI across mild, moderate, and severe OSA, although average physiological control remains inferior to CPAP [408, 409]. From a patient‐centered perspective, oral appliances should not be considered merely as a secondary therapy. Indeed, for individuals with a strong aversion to PAP or poor adherence, real‐world effectiveness may be substantial due to improved nightly use [409, 415]. Comparative outcome data support this interpretation. In a randomized noninferiority trial involving hypertensive patients at elevated cardiovascular risk, MAD was noninferior to CPAP in reducing 24‐h mean arterial pressure, despite weaker average control of respiratory events. This finding highlights that adherence can partially offset the efficacy gap in selected patients [280, 409].

MADs tend to perform best in phenotypes with lower anatomical collapsibility, lower BMI, smaller neck circumference, and positional or REM‐predominant disease. Because long‐term dental and skeletal changes can occur, structured dental surveillance remains mandatory during chronic use [431]. Combination with positional therapy may be especially rational when the device adequately controls events during lateral sleep but residual supine disease persists; importantly, this should be reassessed by follow‐up sleep testing rather than inferred from symptoms alone [409, 431].

A significant proportion of adult OSA cases are positional, with event frequency substantially higher in the supine posture. Positional therapy should be considered a phenotype‐guided adjunctive strategy rather than a universal alternative to first‐line PAP therapy. Nevertheless, it may serve as a stand‐alone option in carefully selected positional phenotypes [411, 412, 414]. This approach is most appropriate for patients with clearly positional OSA, characterized by low nonsupine event rates and mild‐to‐moderate disease severity [175, 411]. In these cases, positional therapy may be used either as a stand‐alone intervention or as part of combination therapy to reduce residual biological burden and facilitate adherence to other treatment modalities [175, 424]. Evidence synthesis demonstrates that positional therapy improves the AHI compared with no treatment and is often better tolerated than CPAP, although CPAP remains superior for overall physiological control [411, 412]. The clinically relevant consideration is not whether positional therapy outperforms PAP on average, but whether a patient's residual nonsupine disease is sufficiently mild for supine avoidance to reduce biological burden without compromising adherence [411, 415]. The most suitable candidates are those with clearly positional OSA, low nonsupine event rates, and mild‐to‐moderate disease. Recent crossover studies support the use of positional therapy as a valuable alternative or adjunct in such patients, particularly when the objective is to reduce PAP requirements or enhance oral appliance effectiveness, rather than to replace PAP entirely in more severe phenotypes [175, 411].

#### 5.2.4 Weight and Metabolic Strategies

Because excess adiposity remains the strongest modifiable risk factor for adult OSA, weight management is a central component of care. Weight loss reduces pharyngeal soft‐tissue load, improves lung‐volume mechanics, and frequently lowers OSA severity and hypoxemia; accordingly, structured dietary, behavioral, and physical activity interventions should form part of routine management in overweight or obese patients [407, 410]. Exercise deserves particular emphasis, even when body weight changes are modest. Recent meta‐analytic evidence supports both aerobic and resistance training as beneficial adjuncts across OSA severities [432]. A separate dose–response meta‐analysis suggests that supervised aerobic exercise totaling roughly 70–100 min per week for at least 12 weeks can yield clinically meaningful improvements in AHI, indicating that exercise should be considered not merely as a general health recommendation but as a mechanistically relevant adjunct even in patients already receiving PAP [432, 433]. Umbrella‐level synthesis further suggests that physical activity may provide some of the largest average improvements in quality of life among nondevice interventions, even when reductions in AHI are more modest than those seen with CPAP [432, 434].

In severe obesity, bariatric or metabolic surgery produces the largest and most durable weight reduction and often markedly improves OSA. However, complete remission is not guaranteed and postoperative reassessment remains essential [410, 435]. Such an aspect is particularly relevant in fibrosis‐prone or cardiometabolic phenotypes, in which residual OSA may continue to drive hypoxic and sympathetic stress despite major metabolic improvement [420, 435]. Pharmacological obesity treatment has also emerged as a major adjunct in obesity‐associated OSA care, although its role should be framed primarily within metabolic and weight‐management pathways rather than as direct endotype‐targeted treatment of OSA itself. In SURMOUNT‐OSA, tirzepatide significantly reduced AHI, body weight, and sleep apnea‐specific hypoxic burden over 52 weeks [84]. In the United States, this was followed by US FDA approval on December 20, 2024, of tirzepatide (Zepbound) for the treatment of moderate‐to‐severe OSA in adults with obesity, to be used in combination with a reduced‐calorie diet and increased physical activity; regulatory status should be specified by jurisdiction if discussed in a global review (US FDA 2024). Subsequent prespecified analyses demonstrated improvement in multiple cardiometabolic risk markers, with mediation analyses suggesting that both weight loss and improvement in OSA metrics contribute to benefit, thereby strengthening the case for treating obesity and sleep‐disordered breathing as interlocking therapeutic targets rather than separate problems [84, 436].

#### 5.2.5 Phenotype‐Guided Adjunctive and Anatomy‐Directed Strategies

Phenotype‐guided, adjunctive, and anatomy‐directed strategies are selected when specific anatomical or physiological drivers, such as PAP intolerance, craniofacial restriction, discrete surgically correctable obstruction, positional dependence, or a favorable upper‐airway phenotype, dominate the clinical picture.

Orofacial myofunctional therapy (OMT) and daytime neuromuscular electrical stimulation (NMES) are regarded as adjunctive options for selected, motivated patients, typically milder phenotypes or primary snoring, rather than as established replacements for PAP or custom MADs. By using structured exercises that target the tongue, soft palate, and facial muscles, OMT aims to improve upper‐airway muscle coordination and endurance. Recent meta‐analytic evidence indicates modest reductions in AHI, along with improvements in sleepiness and snoring. However, these effect sizes are smaller than those observed with PAP and remain highly dependent on patient adherence. Consequently, OMT is most appropriately considered as an adjunct therapy or as an option for motivated patients with mild disease [437]. A 2025 overview and reanalysis of systematic reviews reached a similar conclusion, emphasizing that much of the evidence base remains of low or critically low certainty [437, 438]. A subsequent umbrella review also identified concurrent exercise and OMT as the most consistently beneficial active conservative strategies. However, the overall certainty was again rated as very low to low, supporting selective adjunctive use rather than overstating the efficacy of these interventions as stand‐alone treatments [438, 439]. Daytime NMES of the tongue is attractive because treatment is delivered while awake rather than during sleep. Prospective studies and a randomized sham‐controlled trial suggest good adherence and modest benefit in mild OSA and/or primary snoring, but long‐term comparative data remain limited. Current evidence therefore supports its use mainly in selected mild phenotypes rather than as a substitute for PAP in moderate‐to‐severe disease [440, 441].

Surgical and implantable interventions play a significant role in carefully selected patients. Surgery is particularly indicated for individuals who are intolerant of or unwilling to use CPAP, those with surgically correctable anatomical obstructions, or those seeking a durable non‐PAP strategy as part of a multimodal treatment plan. Current guidelines recommend considering referral to a sleep surgeon for adults with OSA and a BMI below 40 kg/m^2^ when PAP therapy cannot be used effectively [413]. Nasal surgery occupies a distinct but often misunderstood role; guideline‐level evidence supports its benefits in improving symptoms, reducing nasal resistance, and facilitating PAP use in selected patients, but does not support its use as a universally effective stand‐alone treatment for moderate‐to‐severe OSA [413, 427]. The European Respiratory Society guideline on non‐CPAP therapies is particularly valuable, as it formalizes a structured evidence base for custom MADs, positional therapy, myofunctional therapy, carbonic anhydrase inhibitors, bariatric surgery, HNS, and maxillomandibular osteotomy. The guideline emphasizes that non‐CPAP therapies should be selected based on the predominant pathophysiological drivers of disease, rather than being offered as a generic alternative following CPAP failure [414].

In this context, DISE and comprehensive upper‐airway phenotyping can enhance the selection of appropriate procedures and minimize the mismatch between anatomical features and interventions. However, current evidence does not support overly simplistic interpretations, as systematic reviews indicate mixed effects of DISE on overall surgical outcomes. Despite this, DISE remains valuable for identifying specific collapse patterns that inform procedure selection and determine candidacy for HNS [404, 413]. This aspect is particularly important for HNS, where complete concentric palatal collapse is a significant unfavorable pattern. Recent evidence further demonstrates that BMI alone is insufficiently precise to predict or exclude this anatomical phenotype, indicating that candidacy should not be determined solely by anthropometric measures [132, 442].

Maxillomandibular advancement (MMA) remains one of the most effective surgical procedures for OSA because it enlarges the upper airway's skeletal framework and reduces multilevel collapse. Although invasive and requiring careful patient selection, landmark meta‐analytic evidence shows large and durable reductions in AHI together with meaningful symptomatic improvement [443]. More recent evidence suggests that obesity alone should not automatically preclude consideration of MMA, because favorable objective outcomes may still be achieved in selected patients with higher BMI when craniofacial anatomy is a major contributor to airway collapse [443, 444].

HNS represents a different therapeutic logic. As an implantable, nonanatomical therapy that stabilizes the pharyngeal airway through inspiratory tongue protrusion, it has shown clinically important and durable reductions in AHI together with improvements in sleepiness and quality of life in appropriately selected adults [445, 446]. Recent meta‐analyses confirm these benefits in current clinical practice. However, a recent sham‐controlled randomized trial demonstrated no significant short‐term improvement in BP or vascular endpoints, highlighting that robust physiological efficacy may not immediately result in cardiovascular benefit [145, 281].

#### 5.2.6 Emerging Pharmacology and Residual Symptoms

Investigational strategies should be separated explicitly from established care. Beyond antiobesity pharmacotherapy used in obesity‐associated OSA, endotype‐targeted drug development remains investigational. Short‐term randomized studies of atomoxetine/oxybutynin, reboxetine/oxybutynin, and carbonic‐anhydrase inhibition provide proof‐of‐concept that mechanism‐based pharmacotherapy can reduce OSA severity in selected phenotypes; however, these approaches remain investigational, and durable efficacy, long‐term safety, regulatory maturation, and clinically scalable phenotyping remain insufficient for routine clinical use [416, 417]. These data are best interpreted as phenotype‐enrichment signals rather than as justification for broad empirical prescribing, because average treatment effects conceal substantial between‐patient variability in airway collapsibility, loop gain, and arousal characteristics [416, 418]. Carbonic anhydrase inhibition is another promising approach, particularly in patients with high loop gain. In the FLOW Phase 2 trial, once‐daily sultiame improved AHI and oxygenation in moderate‐to‐severe OSA [158]. For now, however, nonobesity pharmacotherapy remains investigational and should not be presented as part of standard OSA management; any future translation into routine practice will require evidence of durable efficacy, acceptable long‐term safety, regulatory clarity, and better bedside phenotyping so that drug selection is driven by mechanism rather than trial‐and‐error prescribing [158, 418].

Residual EDS should not automatically be interpreted as treatment failure. Before symptom‐targeted medication is prescribed, clinicians should verify adequate control of OSA, sufficient nightly treatment exposure, and exclusion of alternative contributors such as insufficient sleep, circadian misalignment, sedating medications, mood disorders, and other hypersomnolence disorders [13, 447]. Network meta‐analysis supports clinically meaningful wakefulness benefits for solriamfetol, pitolisant, and modafinil‐class agents, while pooled patient‐level data support pitolisant efficacy in PAP‐adherent patients with persistent sleepiness. Nonetheless, these agents should be considered tools for symptom control that complement, rather than replace, etiological treatment of OSA [447, 448, 449]. A more recent network meta‐analysis broadly confirms clinically meaningful wake‐promoting effects across approved agents but also highlights relevant trade‐offs in adverse‐event profiles, reinforcing the need for individualized drug selection rather than decisions based on wakefulness efficacy alone [448, 450].

#### 5.2.7 Follow‐Up and Fibrosis‐Oriented Perspectives

Regardless of the initial treatment modality, follow‐up should evaluate objective control of residual respiratory events and nocturnal hypoxemia, adherence, symptom response, and the trajectory of cardiometabolic comorbidity. In routine practice, this means moving beyond device‐reported residual AHI to include oxygenation metrics, weight trajectory, BP, daytime sleepiness, and sleep quality. Organ‐specific markers may be incorporated when clinically justified by the underlying organ context, but fibrosis‐oriented surveillance should not be indiscriminately applied to unselected patients with OSA [13, 408]. Longitudinal follow‐up is especially important after therapy changes because the adequacy of any modality can drift over time with weight change, aging, nasal disease, medication exposure, or progression of the underlying organ disorder [415, 421]. Fibrosis‐oriented implications are biologically plausible and potentially clinically relevant in selected high‐risk phenotypes, but they have not yet been proven to be disease‐modifying and should not redefine routine OSA management pathways. In phenotypes such as IPF or MASLD, OSA treatment should be integrated with organ‐specific management and multidisciplinary follow‐up. Current evidence supports attenuation of ongoing hypoxic and sympathoadrenergic injury, but not definitive reversal of established fibrosis with OSA therapy alone [25, 419, 420].

A final consideration is that current therapies for OSA primarily address the upstream mechanical factors, thereby reducing intermittent hypoxemia, sleep fragmentation, and intrathoracic stress. However, these interventions do not directly suppress downstream fibrotic processes once they become self‐sustaining. For patients with established end‐organ remodeling, combining OSA therapy with organ‐specific antifibrotic, anti‐inflammatory, or metabolic treatments may be justified based on general disease‐management principles. Nevertheless, evidence supporting the ability of such integrated strategies to specifically reverse OSA‐associated fibrosis remains insufficient. Furthermore, umbrella‐level analyses indicate that comparative data on safety, long‐term adherence, and combination strategies are limited across the OSA field. These limitations support the need for pragmatic clinical trials that incorporate physiological, symptomatic, and organ‐specific endpoints rather than relying solely on single outcome measures [434]. In this regard, the feed‐forward interaction between ROS and TGF‐β signaling provides a useful conceptual framework, as it may amplify tissue remodeling even after the initiating insult has been attenuated [242]. Future trials investigating OSA‐associated fibrosis should prioritize the inclusion of biologically susceptible phenotypes and the use of organ‐specific endpoints, such as quantitative imaging, liver stiffness, circulating ECM markers, lung function trajectories, or vascular remodeling indices. Moreover, reliance solely on AHI reduction as a surrogate marker of benefit should be avoided [25, 418, 420].

The management of OSA has evolved from a purely event‐based approach to a more individualized, phenotype‐informed strategy that integrates symptom burden, physiological load, upper‐airway traits, and comorbidity profile, although the degree of phenotypic implementation varies across practice settings. In this context, Table 5 provides a phenotype‐guided overview of the current therapeutic landscape of OSA, summarizing the rationale, evidence‐informed benefits, and major limitations of the principal treatment strategies, with particular attention to fibrosis‐oriented clinical implications and the need for multimodal, individualized care.

**TABLE 5:** Management of obstructive sleep apnea: Established standard therapies, phenotype‐guided adjunctive strategies, investigational approaches, and fibrosis‐oriented clinical caveats.

Therapeutic approach	Core therapeutic logic/ideal phenotype	Main benefits/evidence‐informed value	Key caveats/fibrosis‐oriented notes
Phenotype‐guided principles	Patient‐centered selection integrating AHI/REI with hypoxic burden, TST < 90%, REM/supine pattern, symptom profile, anatomy/endotype, comorbidities, and dynamic modifiers; COMISA should be addressed explicitly [13, 407].	Moves treatment beyond event counts alone and supports clinically meaningful targets: symptom relief, control of nocturnal hypoxemia, cardiometabolic risk reduction, and rational multimodal combinations [415, 421].	Current evidence supports attenuation of ongoing injury rather than proven reversal of established fibrosis [25, 419, 420]. In IPF or MASLD, OSA therapy should be integrated with organ‐specific disease‐modifying care [415, 424].
PAP (CPAP/APAP; bilevel in selected cases)	First‐line for most adults with moderate‐to‐severe OSA. APAP is appropriate for many; bilevel is mainly reserved for pressure intolerance, very high pressure requirements, or coexisting hypoventilation [408].	Provides the greatest average reduction in respiratory events and nocturnal hypoxemia; improves daytime sleepiness and quality of life [408, 421]. Blood‐pressure benefit is clearest in resistant or uncontrolled hypertension [13, 285]. The benefits of all‐cause and recurrent cardiovascular outcomes appear most evident when adherence is adequate [289, 430].	Early troubleshooting of interface issues, nasal symptoms, insomnia, aerophagia, and claustrophobia is essential; telemonitoring can support implementation [425, 426, 427]. Long‐term use patterns still require phenotype‐sensitive reassessment and digitally supported follow‐up pathways [428, 429].
Mandibular advancement devices (MADs)	Established alternative for mild‐to‐moderate OSA and rescue option for selected patients with more severe disease who cannot tolerate PAP; best suited to lower collapsibility, lower BMI, smaller neck circumference, and positional or REM‐predominant phenotypes [408, 409].	Meaningful AHI reduction and potentially strong real‐world effectiveness when better nightly use offsets part of the efficacy gap versus CPAP; randomized evidence supports noninferiority to CPAP for 24‐h mean arterial pressure in selected hypertensive patients [280, 409].	Average physiological control remains inferior to CPAP; custom titration and long‐term dental/skeletal surveillance are required; reassess objectively if combined with positional therapy [431].
Positional therapy	Best suited to clearly positional OSA with low nonsupine event rates and mild‐to‐moderate disease; useful as stand‐alone therapy in selected cases or as an adjunct to reduce PAP requirements or enhance oral‐appliance effectiveness [411, 412].	Improves AHI versus no treatment and is often better tolerated than CPAP [175, 411].	CPAP remains superior for overall physiological control; utility depends on whether residual nonsupine disease is sufficiently mild to lower biological burden without sacrificing adherence.
Weight loss, exercise, and behavioral metabolic care	Core treatment pillar for overweight or obesity‐related OSA, targeting pharyngeal soft‐tissue load and lung‐volume mechanics; exercise is a mechanistically relevant adjunct even when weight loss is modest [407, 410].	Can reduce OSA severity and hypoxemia; aerobic and resistance exercise improve AHI and quality of life; dose–response evidence suggests clinically meaningful benefit with supervised aerobic exercise of roughly 70–100 min/week for at least 12 weeks [432, 433].	Usually adjunctive rather than fully substitutive in higher‐burden OSA; objective reassessment remains necessary because symptom improvement can underestimate persistent residual disease [432, 434].
Bariatric/metabolic surgery and antiobesity pharmacotherapy	Appropriate for severe obesity or clearly obesity‐driven OSA; supports an integrated strategy that treats obesity and sleep‐disordered breathing as interlocking targets.	Bariatric surgery yields the largest and most durable weight reduction [410, 435]. Tirzepatide reduced AHI, body weight, and hypoxic burden, with parallel improvement in cardiometabolic risk markers [84].	Complete OSA remission is not guaranteed; postoperative or postpharmacotherapy sleep reassessment is essential, particularly in cardiometabolic or fibrosis‐prone phenotypes where residual OSA may remain biologically important [84, 436].
OMT and daytime NMES	Conservative adjunctive options for motivated patients, especially mild OSA or primary snoring phenotypes; NMES is attractive because therapy is delivered while awake.	OMT can modestly reduce AHI and improve sleepiness/snoring [437]. NMES may offer good adherence and modest benefit in selected mild phenotypes [440, 441].	Effects are smaller than those achieved with PAP and remain adherence‐dependent; certainty of evidence is generally low; NMES lacks strong long‐term comparative data and is not a substitute for PAP in moderate‐to‐severe disease [438, 439].
Nasal surgery and sleep‐surgery referral	Relevant when PAP cannot be used effectively and when surgically correctable anatomical obstruction is present; particularly useful as an adherence‐facilitating adjunct [413, 427].	Improves nasal resistance and symptoms and can facilitate PAP tolerance/use in selected patients.	Should not be framed as a universally effective stand‐alone treatment for moderate‐to‐severe OSA; referral to non‐PAP surgery should be guided by dominant disease drivers rather than offered generically after CPAP failure [414].
DISE‐guided phenotyping	Use drug‐induced sleep endoscopy and upper‐airway phenotyping to refine procedure selection and identify collapse patterns relevant to surgical planning and HGNS candidacy [404, 413].	Can reduce anatomy‐intervention mismatch and improve selection of anatomically coherent procedures.	Systematic review evidence shows mixed effects on aggregate surgical outcomes; BMI alone is too imprecise to infer or exclude complete concentric palatal collapse [132, 442].
Maxillomandibular advancement (MMA)	Most rational when craniofacial anatomy is a major driver of multilevel airway collapse [443].	Among the most effective surgical procedures for OSA are those that achieve large, durable reductions in AHI and meaningful symptomatic improvement [443, 444].	Invasive and requires careful selection; obesity alone should not automatically preclude consideration, but the anatomical contribution to collapse should be substantial.
Hypoglossal nerve stimulation (HNS)	Implantable, nonanatomical option for appropriately selected adults with PAP intolerance and a favorable airway phenotype.	Durable reductions in AHI together with improved sleepiness and quality of life [445, 446].	Complete concentric palatal collapse is an unfavorable pattern; physiological efficacy does not necessarily translate into early blood pressure or vascular benefits [145, 281].
Emerging endotype‐targeted pharmacology	Investigational mechanism‐based therapy aimed at upper‐airway muscle tone, loop gain, or arousal traits; examples include atomoxetine/oxybutynin, reboxetine/oxybutynin, and sultiame.	Provides proof of concept that selected drugs can reduce OSA severity [416, 417]. Carbonic anhydrase inhibition improved AHI and oxygenation in Phase 2 testing [158].	Average effects conceal marked between‐patient heterogeneity; durable efficacy, long‐term safety, and better bedside phenotyping remain prerequisites for routine use [158, 418].
Residual EDS management	Consider only after confirming adequate OSA control, sufficient treatment exposure, and exclusion of competing causes of sleepiness [13, 447].	Wake‐promoting agents such as solriamfetol, pitolisant, and modafinil‐class drugs can improve wakefulness in carefully selected patients with persistent EDS despite optimized primary therapy [447, 448, 449].	This is a symptomatic management, not etiological OSA treatment; drug choice should be individualized based on the adverse‐effect profile and clinical context [448, 450].
Follow‐up and fibrosis‐oriented multimodal care	Follow‐up should track residual respiratory events, oxygen metrics, adherence, weight/BP trajectory, symptoms, and disease‐specific organ markers; repeat sleep testing after major clinical or therapeutic changes [13, 408].	Allows therapy recalibration as adequacy drifts over time [434]. Supports multidisciplinary integration in phenotypes such as IPF or MASLD [25, 419, 420].	Current OSA therapies primarily address upstream mechanical insults and reduce intermittent hypoxemia, sleep fragmentation, and intrathoracic stress, but they should not be assumed to extinguish self‐sustaining downstream fibrotic programs. In high‐risk phenotypes, combination with organ‐specific antifibrotic, anti‐inflammatory, or metabolic therapy may be reasonable on general disease‐management grounds, but evidence that such integrated strategies specifically reverse OSA‐linked fibrosis remains insufficient [25, 418, 420].

The table organizes the main therapeutic options according to core therapeutic rationale, ideal phenotype, evidence‐informed benefits, and major clinical caveats. Particular emphasis is placed on a phenotype‐guided approach, multimodal treatment integration, and fibrosis‐oriented considerations, highlighting that current OSA therapies primarily target the upstream mechanical insult, whilst downstream organ remodeling and fibrotic sequelae may require additional disease‐specific strategies.

*Abbreviations*: AHI, apnea–hypopnea index; APAP, auto‐adjusting positive airway pressure; BMI, body mass index; BP, blood pressure; COMISA, comorbid insomnia and sleep apnea; CPAP, continuous positive airway pressure; DISE, drug‐induced sleep endoscopy; EDS, excessive daytime sleepiness; HGNS, hypoglossal nerve stimulation; IPF, idiopathic pulmonary fibrosis; MADs, mandibular advancement devices; MASLD, metabolic dysfunction‐associated steatotic liver disease; MMA, maxillomandibular advancement; NMES, neuromuscular electrical stimulation; OMT, orofacial myofunctional therapy; OSA, obstructive sleep apnea; PAP, positive airway pressure; REI, respiratory event index; REM, rapid eye movement; TST < 90%, time spent with oxygen saturation below 90%; UAS, upper‐airway stimulation.

### 5.3 Preclinical and Clinical‐Trial Evidence for Mechanism‐Based and Phenotype‐Guided OSA Interventions

Recent research on mechanism‐based interventions for OSA primarily focuses on animal models of IH‐driven remodeling, pharmacological trials targeting upper‐airway neuromuscular control or ventilatory instability, and the development of metabolic drugs for obesity‐associated OSA. Within these domains, the most clinically informative variables include trial phase, enrollment status, mechanistic target, study objective, respiratory and cardiometabolic endpoints, and the presence or absence of remodeling‐specific outcomes.

Recent animal model evidence is more limited compared with the breadth of clinical trials. The principal preclinical advance in 2026 is a more precise characterization of CIH/IH as a time‐ and dose‐dependent stimulus for vascular remodeling, rather than as a direct model for fibrosis reversal. A 2026 systematic review and meta‐analysis of rodent IH studies reported consistent increases in vascular OS, inflammatory markers, leukocyte infiltration, and apoptosis, along with reduced eNOS expression or activity [451]. A complementary 2026 mouse study demonstrated that IH induces tissue‐specific DNA methylation changes, accelerates epigenetic aging, increases p16Ink4a‐linked senescence, elevates BP, and impairs endothelial and vascular function [452]. These findings enhance the biological plausibility of IH‐driven vascular and organ remodeling, although they do not provide evidence for clinical reversal of established fibrosis.

The most advanced recent clinical programs focus on mechanism‐based pharmacotherapy. AD109, a once‐nightly combination of aroxybutynin and atomoxetine intended to enhance upper‐airway neuromuscular control, has progressed from proof‐of‐concept to Phase 3 trials. The SynAIRgy trial (NCT05813275), published in 2026, demonstrated significant reductions in AHI, ODI, and hypoxic burden among adults with OSA who declined or could not tolerate PAP [453]. LunAIRo (NCT05811247) has been completed with positive topline results, and an open‐label continuation protocol (NCT06566820) is ongoing [453, 454]. Sultiame/sulthiame, an oral carbonic anhydrase inhibitor targeting ventilatory instability and upper‐airway stability, completed the FLOW Phase 2 dose‐finding program (NCT05236842) with dose‐dependent improvements in OSA severity and nocturnal oxygenation [455]. These programs directly address OSA pathophysiology rather than focusing solely on symptom control. However, neither program has yet demonstrated reductions in cardiovascular events, neurocognitive protection, or modification of fibrosis as clinical outcomes.

Metabolic pharmacotherapy has emerged as a major focus of active clinical trials for obesity‐associated OSA. Tirzepatide set the Phase 3 benchmark in the SURMOUNT‐OSA trial by reducing AHI, body weight, hypoxic burden, inflammatory markers, and BP, along with additional patient‐reported benefits [84, 456]. The current metabolic‐development pipeline includes registered Phase 3 OSA programs evaluating eloralintide (NCT07369011), maridebart cafraglutide in the MARITIME‐OSA program (NCT07225686 and NCT07226765), XW003/ecnoglutide (NCT07434050 and NCT07387094), and NNC0487‐0111/zenagamtide in the AMAZE 3/4 program (NCT07571005 and NCT07571109). These trials are designed to test whether pharmacological weight loss reduces OSA severity in participants with overweight or obesity, with trial designs stratified by PAP use, where applicable. At present, no peer‐reviewed publications on OSA outcomes are available for these registered programs.

Additional ongoing or recently reported trials are evaluating phenotype‐guided or pathway‐specific adjunctive therapies. Lorundrostat (NCT06785454) is being studied as an aldosterone synthase inhibitor in OSA patients with hypertension, targeting mineralocorticoid activity, fluid retention, and nocturnal BP regulation. NCT07332442 investigates whether pharmacologically increasing the arousal threshold with eszopiclone can improve CPAP adherence and neurocognitive outcomes, thereby operationalizing an arousal‐threshold endotype. At present, lorundrostat has only company‐reported topline data, whereas the eszopiclone arousal‐threshold trial remains registered but has no published outcome data.

Recent trials and analyses also support more targeted, phenotype‐specific interventions, including wearable‐supported PAP adherence [457], exercise or resistance‐training programs [458, 459], telemedicine‐delivered myofunctional therapy [460], mandibular advancement or DISE‐guided oral‐appliance selection [461, 462], HNS outcomes [463, 464], and symptom‐directed wake‐promoting therapy with solriamfetol for OSA‐related EDS [465]. While these studies advance phenotype‐aware care, most remain limited by small sample sizes, endpoint specificity, or adjunctive design, and do not establish systemic remodeling or antifibrotic disease modification.

Taken together, the available evidence distinguishes mechanism‐based OSA management from therapies directed at fibrosis reversal. Animal studies support IH as a biologically active stimulus for remodeling, while clinical trials increasingly focus on neuromuscular control, ventilatory instability, metabolic load, aldosterone pathways, arousal threshold, and treatment adherence. No recent clinical trial has demonstrated reversal of established fibrosis in the lung, liver, kidney, or heart as a primary outcome. Trials aiming to assess remodeling modification require enrichment for high hypoxic burden or organ vulnerability and must incorporate quantitative endpoints such as lung CT progression, liver stiffness, albuminuria or tubular injury markers, cardiac T1/ECV mapping, ECM biomarkers, and longitudinal organ function trajectories. Table 6 summarizes representative preclinical mechanisms, clinical trial programs, registry identifiers, preliminary findings, and current levels of clinical validation.

**TABLE 6:** Recent preclinical animal and clinical trial evidence for mechanism‐based OSA interventions.

Evidence/pathway	Mechanism of action or pathway targeted	Preclinical animal evidence	Clinical trial evidence: NCT No./status/phase/objective	Available findings	Clinical interpretation
Rodent IH vascular‐remodeling evidence	CIH/IH‐driven vascular oxidative stress, inflammatory activation, leukocyte infiltration, apoptosis, endothelial NO impairment, and exposure‐dose effects.	A 2026 systematic review/meta‐analysis of 52 rodent IH studies reported increased vascular oxidative stress, inflammation, leukocyte infiltration, apoptosis, and reduced eNOS expression/activity [451].	Not applicable. Preclinical evidence only; no NCT, phase, or clinical objective.	Supports IH as a biologically active stimulus for vascular remodeling, with no treatment‐reversal or fibrosis‐specific clinical endpoint.	Relevant as pathway validation, not as clinical proof of antifibrotic benefit.
IH‐induced epigenetic aging and senescence	Tissue‐specific DNA methylation remodeling, epigenetic age acceleration, p16Ink4a‐mediated senescence, endothelial dysfunction, and blood‐pressure elevation.	In 2026, a mouse IH study using C57BL/6J, p16‐reporter, and targeted senescence‐ablation models linked IH to vascular dysfunction and tissue‐specific epigenetic remodeling [452].	Not applicable. Preclinical animal study/preprint; no NCT, phase, or clinical objective.	Early evidence connects IH with senescence and epigenetic remodeling. Findings require peer‐reviewed consolidation and organ‐fibrosis validation.	Strengthens biological plausibility for chronic remodeling but does not establish reversibility after OSA therapy.
AD109: aroxybutynin/atomoxetine	Once‐nightly noradrenergic–antimuscarinic therapy targeting upper‐airway neuromuscular dysfunction and airway collapsibility during sleep.	The mechanistic rationale derives from endotype‐targeted pharmacology, showing that upper‐airway muscle responsiveness can be pharmacologically modified.	SynAIRgy, NCT05813275: completed Phase 3, randomized, double‐blind, placebo‐controlled, 6‐month trial; objective: AD109 vs. placebo in adults with mild‐to‐severe OSA who refused or failed PAP. LunAIRo, NCT05811247: completed Phase 3, 1‐year trial; objective: longer efficacy/safety. Open‐label continuation, NCT06566820: ongoing Phase 3 extension; objective: longer‐term safety/efficacy after SynAIRgy/LunAIRo [453, 454].	SynAIRgy showed significant reductions in AHI, ODI, and hypoxic burden; LunAIRo topline results were positive. NDA submitted to US FDA in 2026.	Most advanced recent mechanism‐based oral pharmacotherapy program for OSA. Not yet evidence for fibrosis, hard cardiovascular endpoints, or neurocognitive disease modification.
Sultiame/sulthiame	Carbonic‐anhydrase inhibition aimed at improving ventilatory stability, lowering loop gain, and supporting upper‐airway stability.	The mechanistic rationale is endotype based, with an expected benefit in patients whose OSA is partly driven by ventilatory control instability.	FLOW, NCT05236842: completed Phase 2 multicenter, randomized, double‐blind, placebo‐controlled dose‐finding trial; objective: compare three once‐daily bedtime doses with placebo in untreated moderate‐to‐severe OSA [455].	FLOW reported dose‐dependent reductions in AHI and improved nocturnal oxygenation with a tolerability profile consistent with carbonic‐anhydrase inhibition.	Promising pharmacological signal, but still investigational. No organ‐remodeling or fibrosis endpoints.
Metabolic/weight‐loss pharmacotherapy	Reduction of adiposity‐related airway loading, tongue/neck fat burden, lung‐volume disadvantage, systemic inflammation, and cardiometabolic risk.	Animal evidence is indirect for OSA‐specific fibrosis. Mechanistic logic is strongest where obesity amplifies OSA and organ vulnerability.	SURMOUNT‐OSA, NCT05412004: completed/published Phase 3 tirzepatide benchmark [84, 456]. Active Phase 3 programs include eloralintide ENLIGHTEN‐3, NCT07369011: recruiting, objective: efficacy and safety in moderate‐to‐severe OSA with overweight or obesity; maridebart cafraglutide MARITIME‐OSA‐1/2, NCT07225686 and NCT07226765: recruiting, objective: efficacy and safety over 52 weeks in OSA with overweight or obesity, respectively with and without PAP; XW003/ecnoglutide, NCT07434050 and NCT07387094: recruiting Phase 3 trials, objective: efficacy and safety in obese participants with OSA, respectively receiving and not receiving PAP; and NNC0487‐0111/zenagamtide AMAZE 3/4, NCT07571005 and NCT07571109: Phase 3 trials, objective: efficacy and safety in excess body weight with OSA, respectively without and with PAP.	Tirzepatide has Phase 3 efficacy data. The newer eloralintide, maridebart cafraglutide, XW003/ecnoglutide, and NNC0487‐0111 programs have no definitive OSA efficacy data available yet.	Major active development axis for obesity‐associated OSA. Expected endpoints are AHI/hypoxic burden, weight, safety, and cardiometabolic markers, not fibrosis reversal.
Aldosterone/mineralocorticoid axis	Aldosterone synthase inhibition, with potential effects on nocturnal hypertension, fluid retention, and upper‐airway obstruction in hypertensive OSA.	Preclinical rationale derives from mineralocorticoid/fluid‐retention models and CIH‐related renal/cardiovascular injury; direct 2026 OSA fibrosis animal data remain limited.	Lorundrostat, NCT06785454: Phase 2 randomized, double‐blind, placebo‐controlled crossover trial; status: enrollment completed with results anticipated/reported in 2026; objective: safety/efficacy of lorundrostat in moderate‐to‐severe OSA with hypertension, including AHI and nocturnal blood‐pressure assessment.	No outcome results available.	Mechanistically relevant for OSA‐hypertension and possible cardiorenal vulnerability. Not yet evidence for remodeling or fibrosis modification.
Arousal‐threshold and CPAP‐response endotyping	Pharmacological elevation of arousal threshold to test whether arousability modifies CPAP adherence and neurocognitive response.	The mechanistic rationale is endotype based: a low arousal threshold may fragment sleep and impair stable respiratory control, while baseline arousability may predict treatment response.	NCT07332442: recruiting randomized controlled trial; objective: determine whether raising arousal threshold with eszopiclone improves CPAP response, adherence, and executive‐function outcomes in OSA.	No results available.	Clinically important test of actionable endotyping. It is not a fibrosis‐directed intervention.
Adherence‐support and digital implementation	Telemedicine, wearable feedback, and behavioral reinforcement to increase effective nightly PAP exposure.	No direct animal component. Mechanistic relevance depends on sustained suppression of nocturnal hypoxemia and arousal/pressure stress.	Recent wearable‐augmented PAP pilot RCT; NCT not reported in the available citation record [457]. Related telemedicine evidence includes a 12‐month CPAP adherence RCT [466]. Objective: improve PAP use in patients with adherence difficulty.	Wearable feedback increased PAP use by approximately 1.5 h/night compared with the waitlist in pilot data.	Clinically relevant because treatment dose conditions the downstream benefit. No fibrosis or organ‐remodeling endpoints.
Exercise, resistance training, and myofunctional therapy	Improvement of cardiometabolic risk, respiratory muscle conditioning, fluid distribution, sleep quality, and upper‐airway neuromuscular coordination.	No direct animal component for OSA fibrosis. Mechanistic plausibility is phenotype‐dependent and indirect.	Recent RCTs reported combined effects of exercise/sleep hygiene and resistance training on OSA severity; NCTs were not available in the citation record [458, 459]. Telemedicine‐delivered myofunctional therapy reported upper‐airway/tongue parameter changes [460]. Objective: reduce OSA severity and improve symptoms or upper‐airway function.	Signals are favorable but small‐sample and phenotype dependent.	Useful adjunctive evidence, not disease‐modifying proof and not fibrosis‐directed.
Mandibular advancement and anatomy‐guided oral appliance selection	Mechanical mandibular protrusion and phenotype/anatomy‐guided airway stabilization.	No direct animal component. The mechanistic rationale is strongest for craniofacial restriction, lower collapsibility, positional disease, and favorable DISE patterns.	2026 severe‐OSA MAD substudy and DISE‐guided MAD selection studies; NCT not available in the citation record [461, 462]. Objectives: compare the effects of MAD vs. CPAP and improve oral‐appliance selection using DISE.	MAD showed better adherence and a greater asleep BP benefit in selected severe OSA, but less AHI suppression than CPAP; DISE‐guided selection improved success rates.	Phenotype‐guided non‐PAP evidence. Respiratory or BP benefits do not support extrapolation to fibrosis reversal.
Hypoglossal nerve stimulation	Implantable stimulation of hypoglossal motor output to improve tongue protrusion and pharyngeal patency.	No direct animal fibrosis component. The mechanism targets upper‐airway neuromuscular compensation rather than systemic remodeling pathways.	CARDIOSA‐12, NCT03359096: completed randomized sham‐controlled cardiovascular trial; objective: 24‐h BP and vascular/cardiovascular outcomes with active vs. sham HGNS [281]. The 2026 secondary analysis assessed sleep‐related function [463]. DREAM, NCT03868618: completed pivotal bilateral HGNS study; objective: respiratory and patient‐reported outcomes in PAP‐intolerant OSA [464].	CARDIOSA‐12 did not show a robust short‐term BP benefit, although patient‐reported sleep‐related outcomes improved in secondary analysis. DREAM reported clinically meaningful AHI/ODI and quality‐of‐life improvements.	Device‐based option for selected PAP‐intolerant phenotypes. Evidence for cardiovascular and fibrosis‐related outcomes remains insufficient.
Symptom‐directed wake‐promoting therapy	Dopamine/norepinephrine reuptake inhibition to reduce residual excessive daytime sleepiness; does not correct airway obstruction.	No direct animal or remodeling rationale for OSA pathophysiology.	Solriamfetol, NCT06103825: completed Phase 3 multicenter, double‐blind, placebo‐controlled trial in Chinese patients with OSA‐related excessive daytime sleepiness; objective: improve Maintenance of Wakefulness Test latency and Epworth Sleepiness Scale scores [465].	Improved wakefulness and subjective sleepiness.	Patient‐centered adjunct only; it does not represent control of OSA pathophysiology or antifibrotic therapy.

Recent preclinical animal and clinical trial evidence for mechanism‐based OSA interventions. Evidence is grouped by pathway, mechanistic target, animal evidence, clinical trial identifier/status/phase/objective, available findings, and clinical interpretation. Clinical trial identifiers are hyperlinked to the corresponding registry entries when available.

*Abbreviations*: AD109, aroxybutynin/atomoxetine; AHI, apnea–hypopnea index; BP, blood pressure; CIH, chronic intermittent hypoxia; CPAP, continuous positive airway pressure; DISE, drug‐induced sleep endoscopy; ECV, extracellular volume; eNOS, endothelial nitric oxide synthase; HGNS, hypoglossal nerve stimulation; IH, intermittent hypoxia; MAD, mandibular advancement device; NCT, National Clinical Trial identifier; ODI, oxygen desaturation index; OSA, obstructive sleep apnea; PAP, positive airway pressure; T1/ECV, native T1 and extracellular‐volume cardiac magnetic resonance mapping.

## 6 Knowledge Gaps and Future Directions

A first major gap across organ systems is the lack of phenotypic resolution rather than simple event counting: the fibrosis literature in OSA still relies heavily on the AHI, even though AHI alone incompletely captures the biological intensity of nocturnal injury [114, 118]. Contemporary OSA research increasingly shows that hypoxic burden, symptom phenotypes, and physiologic endotypes better reflect downstream risk heterogeneity than event frequency alone [6, 112, 118]. Related work also suggests that metrics such as T90 capture clinically relevant nocturnal exposure not reflected by AHI alone [118, 398]. In contrast, most studies linking OSA to fibrotic remodeling in the lung, liver, kidney, and heart remain cross‐sectional and predominantly AHI‐centered, making it difficult to identify which dimension of sleep‐disordered breathing is most relevant to fibro‐inflammatory progression [26, 100, 310, 360]. Future studies should therefore move beyond reliance on AHI alone and adopt integrated physiological profiling that incorporates hypoxemia burden, T90, event duration, sleep fragmentation, sleep‐stage distribution, autonomic response, and endotype structure, and should test whether these measures outperform AHI in predicting organ‐specific fibrotic outcomes [112, 114, 118].

A second major gap is causal inference. Across organ systems, it remains unresolved whether OSA acts primarily as a causal driver of fibrotic remodeling, a disease modifier that amplifies pre‐existing injury, or both. In humans, OSA rarely occurs in biological isolation, and the same patients who carry the highest burden of nocturnal hypoxemia often also have obesity, insulin resistance, hypertension, systemic inflammation, chronic lung disease, or CKD [2, 100]. This overlap is clinically important, but it also creates major confounding when trying to determine whether OSA is an independent driver of fibrosis or a potent amplifier of pre‐existing organ vulnerability. The problem is especially evident in IPF and other fibrotic ILDs, where OSA is common, but nocturnal hypoxemia may arise from both upper‐airway obstruction and restrictive parenchymal disease [22, 310]. Similar complexity exists in MASLD, where shared metabolic risk factors make it difficult to disentangle direct OSA effects from obesity‐driven liver injury, and in CKD, where bidirectional pathophysiology further blurs causal inference [23, 26, 100]. Addressing this question will require prospective, deeply phenotyped cohorts in which standardized sleep testing is paired with organ‐specific biomarkers, imaging, and clinically meaningful fibrosis endpoints, such as serial high‐resolution CT and lung‐function decline in fibrotic ILD, elastography or MRI‐based liver stiffness in MASLD, albuminuria plus renal fibrosis biomarkers in CKD, and cardiac MRI markers such as T1 mapping, ECV, and late gadolinium enhancement in myocardial remodeling studies [26, 310, 360].

A third gap concerns mechanistic resolution, biomarker validation, and translational stratification. Although IH, OS, inflammation, ER stress, mitochondrial dysfunction, and TGF‐beta/HIF‐1α signaling are now established as core organizing pathways [186, 241], no fibrosis‐oriented biomarker panel is currently validated for routine OSA risk stratification or treatment monitoring [201, 258]. In particular, the relative contributions of EMT, EndMT, macrophage polarization, extracellular‐vesicle signaling, and epigenetic or multiomic remodeling have not yet been mapped with sufficient cell‐type and tissue specificity in OSA‐relevant human samples [186, 191, 258, 259]. Single‐cell and spatial transcriptomics, quantitative proteomics, extracellular‐vesicle profiling, and longitudinal blood‐based multiomics should therefore be integrated with sleep phenotyping and treatment‐response data to distinguish transient hypoxia‐induced stress responses from durable profibrotic cell‐state transitions and to support biomarker‐guided interventional studies [114, 191, 258]. Such designs should also be paired with organ‐oriented readouts—including transient elastography, quantitative chest imaging, renal injury biomarkers, and advanced cardiac structural phenotyping—rather than relying on blood biomarkers alone [259].

A fourth gap concerns treatment and interventional design. CPAP remains the cornerstone of OSA therapy [414], but available evidence does not yet support the assumption that correcting airway obstruction is sufficient to reverse established fibrotic remodeling [25, 26, 320]. The therapeutic signal appears to be heterogeneous and may depend on timing, adherence, physiological burden, and the biological stage of the target organ [10, 114]. This limitation is particularly clear in MASLD, where PAP has shown, at best, modest biochemical improvement without consistent effects on imaging‐based steatosis or fibrosis endpoints [26]. More broadly, post hoc analyses suggest that treatment benefit may be concentrated in patients with higher baseline hypoxic burden rather than in unselected AHI‐defined populations [10]. A similar precision‐treatment signal was reported in the ISAACC post hoc analysis, in which a higher hypoxic burden identified patients more likely to derive cardiovascular benefit from CPAP [467]. Future interventional studies should therefore enrich for biologically relevant phenotypes, require objective adherence assessment, and use fibrosis‐relevant endpoints rather than relying solely on conventional sleep outcomes [10, 114]. Combination strategies will likely be especially important, including integration of OSA treatment with weight‐loss pharmacotherapy, cardiometabolic risk reduction, endotype‐targeted interventions, and, where appropriate, organ‐directed anti‐inflammatory or antifibrotic therapies [84, 114].

A fifth and final gap concerns implementation and longitudinal monitoring. Wearable and contactless technologies, especially when combined with artificial intelligence, are rapidly improving and may help scale repeated assessment of hypoxemia, sleep fragmentation, and treatment response [405, 406]. More recent syntheses likewise indicate continued expansion of wearable/contactless and AI‐enabled approaches for OSA detection and follow‐up [468, 469]. However, these tools are not yet a substitute for rigorous phenotyping, particularly in multimorbid patients with fibrotic lung disease, cardiometabolic disease, or CKD, where signal interpretation is more complex. Their most immediate value may be to complement rather than replace PSG, enabling repeated out‐of‐laboratory monitoring and more dynamic risk stratification [405, 406]. Future work should define OSA‐associated fibrosis not as a generic complication of sleep‐disordered breathing, but as a precision‐medicine problem in which the relevant exposure, vulnerable organ, dominant endotype, and modifiable pathway are identified before therapy is selected.

## 7 Conclusion

The collected data suggest that OSA should be viewed as a systemic disorder with potential fibro‐inflammatory implications in certain high‐risk phenotypes, while recognizing that evidence for causality and treatment effectiveness varies across organ systems [26, 100, 310, 360]. Across organs, IH, OS, immune activation, ER stress, and TGF‐beta/HIF‐1α‐centered signaling provide a biologically coherent framework through which OSA may amplify maladaptive tissue repair and ECM remodeling in susceptible settings [186, 241]. However, current evidence does not support fibrosis as a uniform or general consequence of OSA across all organs and clinical contexts [26, 100, 310, 360]. Instead, the most consistent clinical message is that OSA may act as a modifiable amplifier of pre‐existing vulnerability, particularly when nocturnal hypoxemic burden is high and interacts with organ‐specific susceptibility and comorbidity load [10, 22, 26, 118].

Accordingly, the clinical and translational future of this field lies in moving beyond AHI‐centered thinking toward a more precision‐medicine‐oriented framework [112, 114, 118]. Better risk stratification will likely require integrating physiological burden metrics, symptom phenotypes, endotypes, organ‐specific biomarkers, and remodeling endpoints tailored to the tissue under study [112, 191, 360]. Therapeutic progress will probably depend on combination strategies that pair OSA treatment with broader cardiometabolic and organ‐protective interventions rather than assuming CPAP alone will modify downstream remodeling [10, 84, 114]. Within this framework, OSA may become a clinically relevant component of broader organ‐protective strategies in susceptible patients, but whether OSA‐directed therapy can prevent or slow established fibrotic remodeling remains to be demonstrated by dedicated longitudinal and interventional studies [10, 84, 114].

## Author Contributions

G.P. conceived the manuscript. N.H.T.T. and T.H.G.P. prepared the initial draft and contributed equally to this work. A.H.E. critically revised the manuscript. P.M. and S.C. contributed to figure preparation and visualization. A.M.P. and R.G. critically revised the manuscript and contributed to figure preparation and visualization. G.P. supervised the work, coordinated manuscript development, and critically revised the final version. All authors have read and approved the final manuscript.

## Ethics Statement

This article is a narrative review and did not involve human participants, animal experiments, or newly generated clinical or experimental data.

## Conflicts of Interest

The authors declare no conflicts of interest.

## Data Availability

The authors have nothing to report.
